# Projections of temperature-attributable premature deaths in 209 U.S. cities using a cluster-based Poisson approach

**DOI:** 10.1186/s12940-015-0071-2

**Published:** 2015-11-04

**Authors:** Joel D. Schwartz, Mihye Lee, Patrick L. Kinney, Suijia Yang, David Mills, Marcus C. Sarofim, Russell Jones, Richard Streeter, Alexis St. Juliana, Jennifer Peers, Radley M. Horton

**Affiliations:** Department of Environmental Health, Harvard School of Public Health, Boston, MA USA; Department of Epidemiology, Harvard University, Boston, MA USA; Columbia Climate and Health Program, Mailman School of Public Health at Columbia University, New York, NY USA; Abt Associates, 1881 Ninth Street, Suite 201, Boulder, CO 80302 USA; Climate Change Division, U.S. Environmental Protection Agency, Washington, DC USA; Center for Climate Systems Research, Columbia University, New York, NY USA

**Keywords:** Temperature-attributable premature mortality, United States, Climate change

## Abstract

**Background:**

A warming climate will affect future temperature-attributable premature deaths. This analysis is the first to project these deaths at a near national scale for the United States using city and month-specific temperature-mortality relationships.

**Methods:**

We used Poisson regressions to model temperature-attributable premature mortality as a function of daily average temperature in 209 U.S. cities by month. We used climate data to group cities into clusters and applied an Empirical Bayes adjustment to improve model stability and calculate cluster-based month-specific temperature-mortality functions. Using data from two climate models, we calculated future daily average temperatures in each city under Representative Concentration Pathway 6.0. Holding population constant at 2010 levels, we combined the temperature data and cluster-based temperature-mortality functions to project city-specific temperature-attributable premature deaths for multiple future years which correspond to a single reporting year. Results within the reporting periods are then averaged to account for potential climate variability and reported as a change from a 1990 baseline in the future reporting years of 2030, 2050 and 2100.

**Results:**

We found temperature-mortality relationships that vary by location and time of year. In general, the largest mortality response during hotter months (April – September) was in July in cities with cooler average conditions. The largest mortality response during colder months (October–March) was at the beginning (October) and end (March) of the period. Using data from two global climate models, we projected a net increase in premature deaths, aggregated across all 209 cities, in all future periods compared to 1990. However, the magnitude and sign of the change varied by cluster and city.

**Conclusions:**

We found increasing future premature deaths across the 209 modeled U.S. cities using two climate model projections, based on constant temperature-mortality relationships from 1997 to 2006 without any future adaptation. However, results varied by location, with some locations showing net reductions in premature temperature-attributable deaths with climate change.

**Electronic supplementary material:**

The online version of this article (doi:10.1186/s12940-015-0071-2) contains supplementary material, which is available to authorized users.

## Background

Climate change is projected to lead to increased temperatures in the United States over the coming decades. Temperature and mortality are known to be linked, with notable events such as the 2003 European heat wave resulting in thousands of deaths [1], but there is also evidence for mortality effects at temperatures that are not extreme [2, 3]. Therefore, there is interest in the impacts of these future temperature changes on human health. Policymakers within the United States are particularly interested in domestic impacts.

Generally, future projections of heat mortality in the United States rely on historically developed temperature-mortality relationships based on epidemiological studies. Studies have examined the impact of extreme temperature events (e.g., [4, 5]) as well as the nature of the relationships over longer time periods in multiple locations for both cold and hot temperatures [2, 6–9].

However, there have only been a handful of studies that have provided mortality projections for a large fraction of the population within the United States [9–12]. Even fewer have considered the implications of temperature excursions in both the hot and cold directions [6, 11–13]. Some of these studies only considered impacts above temperature thresholds; others accounted for changes across all temperatures. All of the studies that projected future heat mortality found large expected increases in mortality. Of the studies that projected both heat and cold mortality, three of the four found net mortality increases mortality, with one showing a net nationwide decrease in mortality due to climate change [13].

We undertook this effort because of the paucity of work addressing temperature-mortality relationships for the U.S. urban population as a whole addressing mortality effects in winter as well as summer, or addressing mortality for non-extreme temperatures. In addition, this study incorporates temperature-mortality relationships that vary by time of year as well as location and stabilizes city-specific estimates by combining strength across many cities with similar weather patterns, both of which should improve projections relative to prior work. In particular, the development and use of month-specific mortality functions is a relatively new approach.

In order to develop future projections, first, we developed city and climate region-specific temperature-mortality relationships for each month of the year by analyzing 34 years of weather and mortality data from 209 cities. We then combined these relationships with climate model outputs to project the daily mortality response to future climate change based on conditions in each city. Results were then aggregated to support comparisons and draw general conclusions.

## Methods

### Data

We obtained daily death record data with information on the county and cause of death from 1973 through 2006 from the National Center for Health Statistics. We defined cities as groupings of one or more counties in the urban area. City definitions were consistent with those defined in a previous study by a subset of the authors [2] (for details of the groupings see Additional file 1: Table S1). A daily death count record for 209 U.S. cities in this period was developed by assigning counties to specific cities for reporting. Where a city was contained in a single county the daily death count reflected deaths in that county. Where a city incorporates multiple counties, we combined daily mortality totals from each county to produce the city total. All listed causes of death were included in daily death totals except for deaths attributed to external causes (i.e., International Classification of Diseases (ICD)-10 codes V01-Y98 and ICD-9 codes > 800) [14].

We used airport station meteorological data downloaded from the National Oceanic and Atmospheric Administration [15] to create a daily record of average temperature for each city from 1976 to 2005. Average daily temperature was calculated from the data as the mean of the daily maximum and minimum temperature. The same weather stations used in prior research incorporating these cities were used when possible [2]. If an original monitor was missing data, we used data from the next nearest weather station within 60 km to complete the daily record. In constructing the historical record, we screened the minimum and maximum values to identify and address implausible values.

We developed projections of future daily average temperature for each city using a 1°, Bias-Corrected Constructed Analogues dataset (BCCA; [16]) from the World Climate Research Programme’s Coupled Model Intercomparison Project phase 5 (CMIP5). We ultimately selected data from the Geophysical Fluid Dynamic Laboratory—Coupled Physical Model 3 (GFDL-CM3) and the Model for Interdisciplinary Research on Climate (MIROC5) with a Representative Concentration Pathway (RCP) value of 6.0 W/m^2^ [17] based on prior experience with earlier versions of these models which suggested they could project relatively different future climates in the United States [12]. We selected the 6.0 W/m^2^ RCP from available options as part of a coordinated climate change and human health research modeling effort [18].

**Fig. 1:**
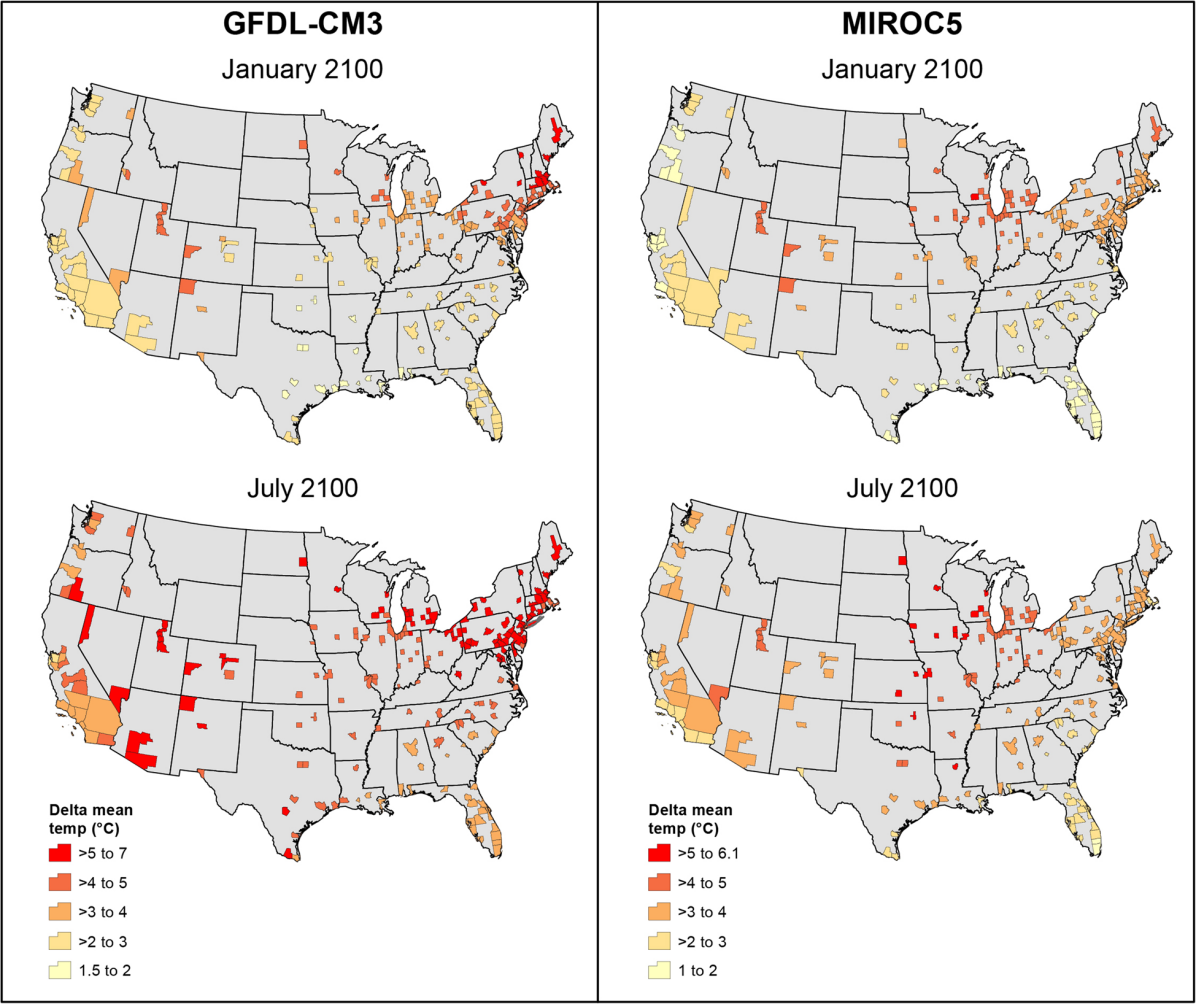
Projected temperature differences by model from 1990 baseline to 2100 in January and July. *Legend:* This figure shows projected temperature differences between the 1976–2005 model baseline, reported as 1990 and 2086–2100, reported as 2100, for January and July by city for the GFDL-CM3 and MIROC5 climate models

We developed climate projections from the models for four time slices to provide supporting data for an estimate of annual impacts in a baseline and three designated future reporting years. The baseline and future reporting years, with the associated time slices shown in parenthesis, were respectively: 1990 (1976–2005), 2030 (2016–2045), 2050 (2036–2065) and 2100 (2086–2100). We used thirty year slices roughly centered on the reporting year when possible, the exception being the time slice for 2100, since projections after 2100 are unavailable. With daily average temperature values from the models’ outputs we calculated a difference in daily average temperature subtracting the mean of the average temperature from 1976 to 2005 for a calendar day from the modeled average temperature for the same day in a future year. We then added this difference to the calculated mean of the average temperature value for that city’s weather station for that day, based on the actual temperature observations from 1976 to 2005. We repeated this process for each city, for each calendar day and for each year within a time slice for a future reporting year. For example, to calculate the projected average temperature on January 17, 2054 in Boston, MA from the MIROC5 model, the model’s projected average temperature for the day minus the mean of the average temperature for that day from MIROC’s modeling of the period 1976–2005 was added to the average temperature for Boston on January 17 based on actual weather station observations for the period 1976–2005. Using this method, Fig. 1 shows the average of the projected daily changes in each day’s average temperature for each study city in January and July 2086–2100 associated with the GFDL-CM3 and MIROC5 models compared to the 1990 baseline.

To calculate temperature-attributable mortality, we required a measure of the exposed, all-age population in each city and the associated daily mortality rate. The 2010 population for each city was extracted from the Integrated Climate and Land Use (ICLUS) A1 population scenario using features within BenMAP [19, 20]. We also used BenMAP to develop city-specific all-age, all-cause mortality rates for 2010 [20].

### Modeling the temperature-mortality relationship

**Fig. 2:**
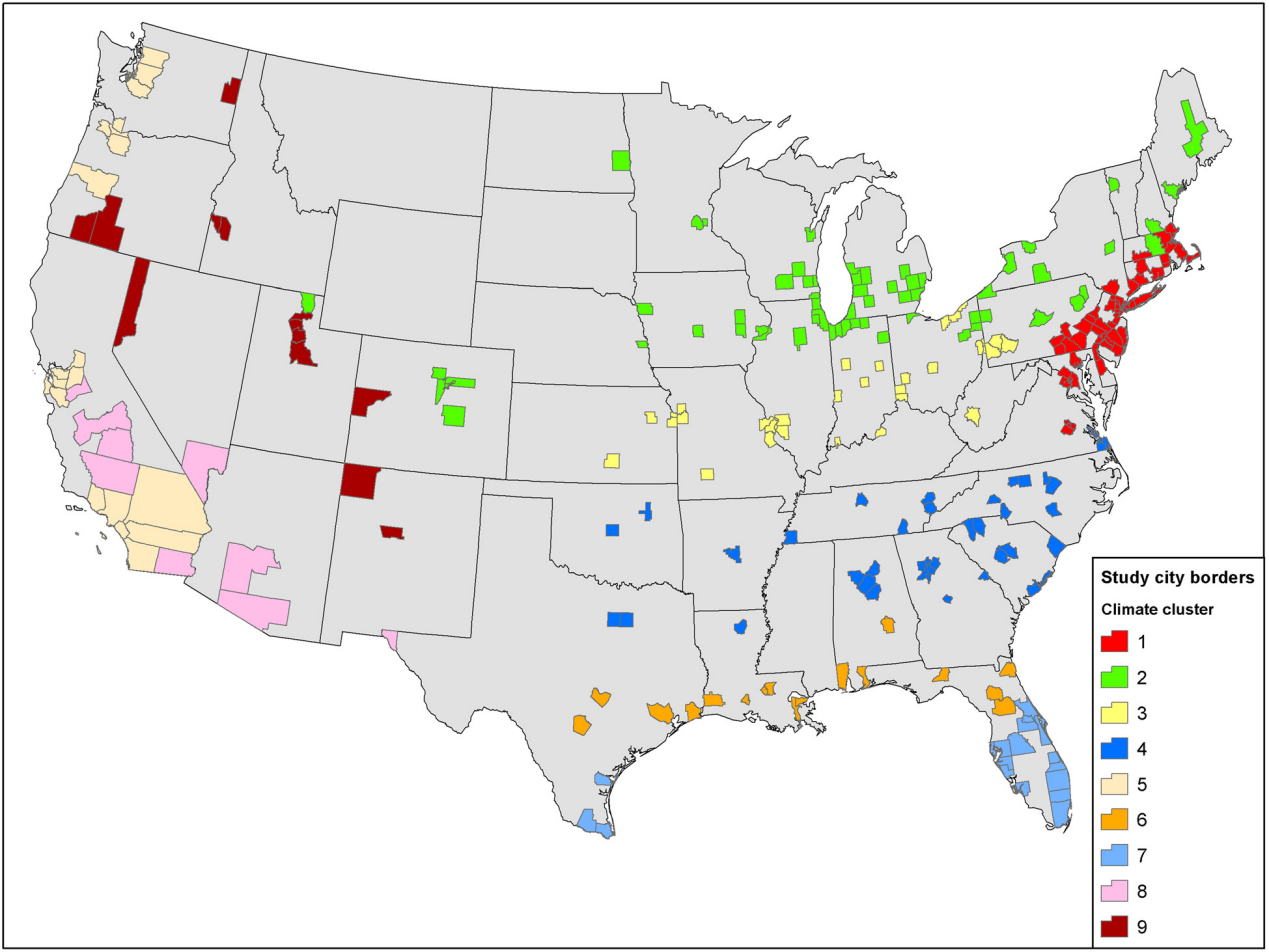
County borders for the 209 study cities in the nine cluster groups. *Legend:* This figure identifies the borders for the 209 cities considered in the study and the assignment of cities to climate-based cluster groups

Because many of the 209 cities were small, with low daily mortality counts, we sought to improve the statistical precision of our effect estimates by pooling within clusters, and using the pooled effect estimates to shrink the variation in individual city results, as described below. Clustering is also consistent with previous results that have observed regional differences in effect estimates in multi-city studies (e.g., [2, 3, 7]). Accordingly, we defined nine climate clusters using an agglomerative hierarchical approach that took as input city-specific seasonal temperatures, humidity and within-season standard deviations of temperature and humidity. This method starts by defining each data point to be a cluster, and then combines existing clusters at each step through the single linkage method using PROC CLUSTER with Ward’s minimum-variance method in SAS 9.3 (Copyright © 2012 SAS Institute Inc., SAS Campus Drive, Cary, North Carolina 27,513, USA). Examining the spatial distribution of the initial 8 clusters, we then divided one cluster because it consisted of two geographically separate city groups (final clusters 1 and 9). Fig. 2 presents the county borders that define the cities in our analysis, color-coded by their assignment into the 9 final cluster groups. Table 1 provides cluster-specific descriptive weather and mortality statistics for the period 1973–2006. Collectively, the 209 study cities accounted for approximately 189 million residents, or 60 % of the 314 million residents of the contiguous United States in the 2010 ICLUS A1 scenario.

**Table 1:** Average cluster weather and mortality characteristics (1973–2006)

Cluster	Mean daily average temperature °C (S.D.)	Mean daily mortality (S.D.)
1	12.0 (9.6)	23 (34)
2	9.4 (10.8)	12 (23)
3	12.6 (10.4)	13 (14)
4	16.6 (8.8)	10 (9.1)
5	14.6 (5.8)	29 (34)
6	20.2 (7.2)	10 (11)
7	23.2 (5.2)	15 (12)
8	19.6 (8.6)	13 (13)
9	11.2 (9.6)	4.2 (3.7)

Research has indicated temperature associations with daily deaths over the course of a year are nonlinear and are often U- or J-shaped, reflecting an increased mortality effect at both relatively cold and hot temperatures [2, 3, 6, 7]. The mortality response to temperature also varies by location and time [2, 7, 8, 21]. As a result, our mortality modeling framework accounted for these factors using a computational framework that balanced flexibility with relative ease of implementation for developing premature mortality projections.

Our model development and mortality projections were completed in stages. First, we evaluated the relationship between daily deaths and average daily temperatures by month in each city. We captured potential non-linearity in this relationship by fitting a Poisson regression to the daily death counts with a piecewise linear spline of average temperature with the spline knot at the median temperature for the month in the cluster (*Tm*_*ij*_). We did this for the same day average temperature (lag 0) and the mean of average temperature over the five days preceding the death (*Tm15*_*ij*_, lag 1–5) as prior studies indicate that two temperature terms, one to capture the immediate effects and one the delayed effects, are needed in this type of mortality study [22]. In summary, we fitted the following generalized linear spline model to *each city in each month*:

$$ \begin{array}{l}Ln\left(E(Y)\right)={\beta}_0 + {\beta}_1Cen\_ Temp + {\beta}_2{\left(Cen\_ Temp-T{m}_{ij}\right)}_{+} + {\beta}_3Cen\_ Temp15 + \\ {}\kern5em {\beta}_4{\left(Cen\_ Temp15- Tm{15}_{ij}\right)}_{+} + {\beta}_5 Year5+{\beta}_6 Time + {\beta}_7DOW + \\ {}\kern5em {\beta}_8Cen\_ Temp\times Year5 + {\beta}_9{\left(Cen\_ Temp-T{m}_{ij}\right)}_{+}\times Year5 + \\ {}\kern5em {\beta}_{10}Cen\_ Temp15\times Year5 + {\beta}_{11}{\left(Cen\_ Temp15- Tm{15}_{ij}\right)}_{+}\times Year5,\end{array} $$

Where ()_+_, is a truncated line function, such that *a-b*_*+*_ is equal to *a-b* when *a* > *b* and is equal to zero when *a* < *b. Temperature*_*k*_ is the temperature in city k on the day of death, and Temperature15k is the average temperature between lag1 and lag5 previous to the day of death. *Tm*_*ij*_ is the median temperature for cluster i in month j on the day of death. *Tm15*_*ij*_ is the median temperature for cluster *i* in month *j* for the moving average between lag1 and lag5.

We also centered the average temperature variable to the mean of the cluster and month by subtracting temperature from the mean temperature of cluster/month. Therefore, *Cen_Temp*_*k*_ is the centered temperature on the same day of death. *Cen_Temp15k* was also centered using the mean of temperature lag1 - lag5 for the corresponding cluster and month. The temperature variables were centered to ensure consistency of the interaction with time periods. *Year5* is a categorical variable for each five-year interval between 1973 and 2006. *DOW* is the day of the week.

After we acquired the coefficients from each city from the first stage modeling, we performed the following meta-regression in a second stage to gain stability:

$$ {\beta}_{im}={\gamma}_0 + {\gamma}_1 Cluster + {\gamma}_2 Month + {\gamma}_3 Year5 + {\gamma}_4Tmp\_Av{e}_{im} + {\gamma}_5 Cluster\times Month $$

Where *β*_*im*_ is the coefficient (which is also the natural logarithm of expected rate ratio) from the first stage model in city i and month m; *Cluster* is the cluster identification from 1 to 9; *Month* is the month; *Year5* is the 5-year time period, and *Tmp_Ave* is the average temperature of the city, by month and by each five-year period. The pooled effect estimates derived from the meta-regression were then used, along with the original city-specific results, to derive weighted Bayesian posterior estimates for each city.

### Mortality projection

**Fig. 3:**
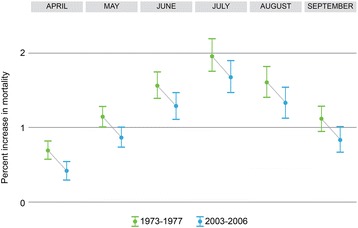
A comparison of the mortality effect for temperatures warmer than the median by month in cluster 1 for the periods 1973–1977 and 2003–2006. *Legend:* This figure shows a reduced mortality impact of high summer temperatures over time in our study

As a first step in developing mortality projections, we evaluated if the temperature impact on mortality had changed over the course of the 34-year period in the data record based on the results of the second stage model described above. As an example, Fig. 3 compares the calculated percent increase in mortality associated with a 1 °C increase in same day average temperature above the median in the hotter months within Cluster 1 from the first five-year period (1973–1977) to the last data period (2003–2006) in the data record for temperatures. This figure shows a downward shift in the mortality response to higher temperatures within cluster 1 over time. This evidence of possible adaptation in the form of a changing response to temperature over time was observed in the other clusters for the hotter and colder months. As a result, we chose to use the most recent decade of data (1997–2006), rather than the full time-period as the basis for developing the relationships used to project future mortality (see Results section for additional discussion). The same-day effect (lag0) appeared to capture most of the impact of hotter temperatures. In contrast, the delayed effect, implemented as the lag 1–5 term, showed more relevance to the effects of premature deaths in colder months. For this reason, in our projections of future mortality impacts, we used only the lag0 slopes for the warmer months and the lag 1–5 slopes for the colder months.

With the data period defined, we reran the initial model to obtain city and month-specific estimates. Second, we repeated the meta-analysis with the resulting city-specific estimates without any covariates to summarize the estimates by cluster and month. Third, we adjusted the city-specific estimates using the results from meta-analysis using an empirical Bayes approach that effectively generates month-specific, weighted, temperature-premature mortality response coefficients in each city for each month [23].

We calculated premature deaths (*ΔDeath*) attributable to temperature by multiplying the baseline mortality rate (*y*_*0*_), size of the exposed population (*Pop*), and the attributable fraction (*AF*) for each city, as followxs:

$$ \varDelta \mathrm{Death}={y}_0\times AF\times Pop $$

For our analyses, we converted the original annual mortality rates extracted from BenMAP to monthly mortality rates by constructing weights based on observed average monthly death counts in each city during the baseline period, as follows:

$$ weight = \frac{Death\  Coun{t}_i}{{\displaystyle {\sum}_{i=1}^{12}} Death\  Coun{t}_i} $$

Where *Death Count*_*i*_ is the number of deaths observed in month *i.* To create the monthly mortality rate values that we used for the projections, we multiplied the calculated weights by the original annual mortality rate. The resulting monthly mortality rates were converted to a daily equivalent by dividing by the number of days in the month.

The AF, which characterizes the fraction of the disease burden attributable to the risk factor, was defined as:

$$ \mathrm{A}\mathrm{F} = \frac{RR-1}{RR} $$

Based on evidence from prior research showing a stronger relationship between mortality and the same-day exposure for hotter weather and a lagged exposure for generally colder weather we divided the year into hotter (April–September) and colder (October–March), months. We then used the parameter estimates from the regression models described above to compute the relative risks (*RR*) as detailed below:For the hotter months (April–September):

$$ RR = \left\{\kern0.5em \begin{array}{c}\hfill {e}^{\beta_1\times Cen\_Tem{p}_k}\kern5.25em \left( Temperatur{e}_k<T{m}_{ij}\right)\hfill \\ {}\hfill {e}^{\left({\beta}_1+{\beta}_2\right)\times Cen\_Tem{p}_k}\kern4.1em \left( Temperatur{e}_k\ge T{m}_{ij}\right)\hfill \end{array}\right. $$

For the colder months (October–March):

$$ RR = \left\{\kern0.5em \begin{array}{c}\hfill {e}^{\beta_3\times Cen\_ Temp{15}_k}\kern5.25em \left( Temperature{15}_k< Tm{15}_{ij}\right)\hfill \\ {}\hfill {e}^{\left({\beta}_3+{\beta}_4\right)\times Cen\_ Temp{15}_k}\kern4em \left( Temperature{15}_k\ge Tm{15}_{ij}\right)\hfill \end{array}\right. $$

Definitions for the terminology in these equations appear in the section above.

Using the projected daily temperatures for each time slice, we then calculated the RRs and resulting premature deaths by day for each city. Premature deaths were then aggregated by month and summed for the hotter and colder months in a given year and for the year respectively. Results from the years within a time slice were then averaged to generate the values for the reporting year by city and cluster.

## Results

**Fig. 4:**
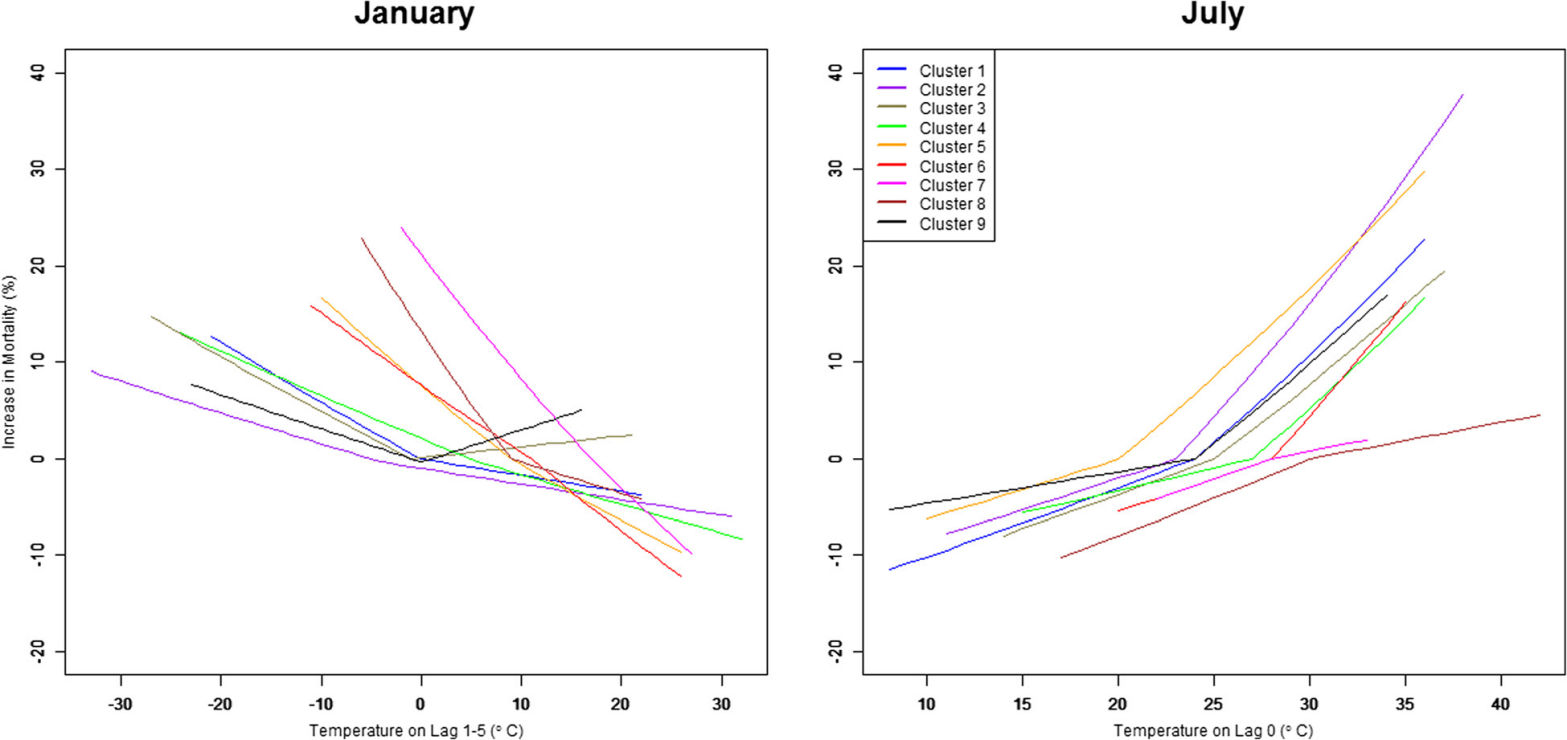
Month and cluster differences in temperature mortality effects. *Legend:* This figure shows the different premature mortality response to temperature by month and cluster. The kink in the response line for a cluster is at the median temperature for that cluster in that month based on 2003–2006 weather data

We found that the impact of changing future daily average temperatures on premature deaths varied by cluster. Figure 4 reflects this variation showing the Bayes-adjusted, cluster-specific, results for the temperature-attributable mortality response to different average temperatures in January and July. Within Fig. 4, the January results show the clusters in generally colder regions (e.g., [1–3, 9]) having a smaller mortality response per degree below the median, as well as a colder median, compared to warmer climate clusters. In contrast, in July, these generally colder clusters showed a larger premature mortality response to temperatures above the monthly median relative to the generally warm clusters. Interestingly, Cluster 5, located along the west coast, shows a comparable temperature mortality response to colder-region Clusters 1, 2 and 9 in July.

Projected changes in temperatures for January and July in 2086–2100 (Fig. 1) provide a sense of the underlying warming projected by the GFDL-CM3 and MIROC5 models. These results show that anticipated warming will vary by season and location. Further, while the models’ patterns and ranges of projected temperature increases are generally similar, there are important differences. For example, GFDL-CM3 generally projects larger temperature increases in the Eastern and Western regions, particularly in July, while MIROC5 projects greater warming in the Central region.

**Fig. 5:**
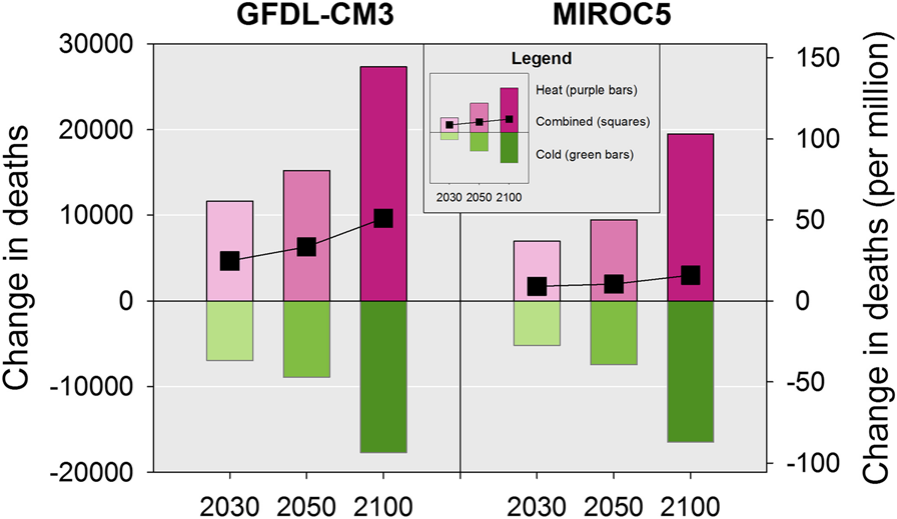
*Title:* Projected change in premature deaths across study cities from the GFDL-CM3 and MIROC5 climate models. *Legend:* This figure presents the projected change in total premature temperature-attributable deaths and the equivalent deaths per million study city residents (left and right sides of the y axis respectively) for future reporting years (x axis) relative to the 1990 baseline. The results for the GFDL-CM3 model are presented in the left panel and the MIROC5 model in the right panel. Changes in premature deaths for the hotter months of April – September (heat) are presented in purple, and changes for the colder months of October – March (cold) are presented in green. The combined effect is shown with the black squares

Changes in premature mortality from all cities and both climate models for the 2030, 2050 and 2100 reporting years relative to 1990 for the hotter (April–September), and colder (October–March) months, are summarized in Tables 2, 3 and Fig. 5. These results show roughly similar projected changes in average annual premature deaths for the colder months across models for the reporting years with a larger mortality impact in the hotter months from the GFDL-CM3 model.

**Table 2:** Projected change in premature temperature-attributable deaths by cluster and season for 2030, 2050 and 2100, relative to the 1990 baseline based on climate data from the GFDL-CM3 model

Change in premature deaths in future reporting years relative to the 1990 baseline reporting period										
Cluster	Population (2010)	Cold (October–March)			Heat (April–September)			Combined		
		2030	2050	2100	2030	2050	2100	2030	2050	2100
1	43,376,142	−2313	−2749	−5379	3369	4255	7645	1055	1506	2266
2	31,613,703	−874	−1061	−2330	2541	3354	5922	1667	2293	3592
3	14,372,496	−508	−604	−1320	1062	1345	2397	554	741	1078
4	21,143,442	−726	−870	−1782	1090	1369	2514	364	499	732
5	36,479,539	−766	−1170	−2048	1760	2512	4483	994	1342	2435
6	11,604,148	−412	−502	−1145	528	675	1401	116	172	256
7	15,148,594	−858	−1159	−2152	590	778	1377	−268	−381	−775
8	10,736,551	−440	−688	−1269	471	629	1038	31	−59	−230
9	4,774,894	−84	−131	−256	235	314	533	151	183	277
All	189,249,510	−6981	−8933	−17,680	11,646	15,229	27,312	4664	6296	9632
Change in premature deaths per million study city residents in future reporting years relative to the 1990 baseline reporting period (2010 populations in all reporting periods)										
Cluster	Population (2010)	Cold (October–March)			Heat (April–September)			Combined		
		2030	2050	2100	2030	2050	2100	2030	2050	2100
1	43,376,142	−53.3	−63.4	−124.0	77.7	98.1	176.2	24.3	34.7	52.2
2	31,613,703	−27.7	−33.5	−73.7	80.4	106.1	187.3	52.7	72.5	113.6
3	14,372,496	−35.3	−42.0	−91.8	73.9	93.6	166.8	38.6	51.5	75.0
4	21,143,442	−34.3	−41.1	−84.3	51.6	64.7	118.9	17.2	23.6	34.6
5	36,479,539	−21.0	−32.1	−56.1	48.3	68.9	122.9	27.3	36.8	66.8
6	11,604,148	−35.5	−43.3	−98.7	45.5	58.1	120.8	10.0	14.8	22.1
7	15,148,594	−56.6	−76.5	−142.1	38.9	51.4	90.9	−17.7	−25.2	−51.1
8	10,736,551	−41.0	−64.1	−118.1	43.8	58.6	96.7	2.9	−5.5	−21.5
9	4,774,894	−17.7	−27.4	−53.7	49.2	65.7	111.6	31.5	38.3	57.9
All	189,249,510	−36.9	−47.2	−93.4	61.5	80.5	144.3	24.6	33.3	50.9

**Table 3:** Projected change in premature temperature-attributable deaths by cluster and season for 2030, 2050 and 2100, relative to the 1990 baseline based on climate data from the MIROC5 model

Change in premature deaths in future reporting years relative to the 1990 baseline reporting period										
		Cold (October–March)			Heat (April–September)			Combined		
Cluster	Population (2010)	2030	2050	2100	2030	2050	2100	2030	2050	2100
1	43,376,142	−1479	−2168	−5279	1677	2170	5120	197	2	−160
2	31,613,703	−916	−1191	−2581	1535	1995	4240	620	803	1659
3	14,372,496	−478	−626	−1357	709	838	1720	231	212	363
4	21,143,442	−490	−746	−1645	644	903	1850	153	157	204
5	36,479,539	−738	−997	−1794	1313	1947	3422	576	950	1628
6	11,604,148	−281	−446	−973	341	505	1069	60	59	97
7	15,148,594	−298	−625	−1530	300	458	964	2	−167	−566
8	10,736,551	−428	−539	−1073	301	458	779	−127	−82	−295
9	4,774,894	−99	−130	−236	129	190	345	31	60	109
All	189,249,510	−5207	−7469	−16,468	6950	9462	19,509	1743	1994	3042
Change in premature deaths per million study city residents in future reporting years relative to the 1990 baseline reporting period (2010 populations in all reporting periods)										
		Cold (October–March)			Heat (April–September)			Combined		
Cluster	Population (2010)	2030	2050	2100	2030	2050	2100	2030	2050	2100
1	43,376,142	−34.1	−50.0	−121.7	38.7	50.0	118.0	4.6	0.0	−3.7
2	31,613,703	−29.0	−37.7	−81.6	48.6	63.1	134.1	19.6	25.4	52.5
3	14,372,496	−33.3	−43.6	−94.4	49.4	58.3	119.7	16.1	14.7	25.3
4	21,143,442	−23.2	−35.3	−77.8	30.4	42.7	87.5	7.3	7.4	9.7
5	36,479,539	−20.2	−27.3	−49.2	36.0	53.4	93.8	15.8	26.0	44.6
6	11,604,148	−24.2	−38.4	−83.8	29.4	43.5	92.1	5.2	5.1	8.3
7	15,148,594	−19.7	−41.3	−101.0	19.8	30.2	63.6	0.1	−11.0	−37.4
8	10,736,551	−39.9	−50.2	−100.0	28.1	42.6	72.6	−11.8	−7.6	−27.4
9	4,774,894	−20.7	−27.3	−49.4	27.1	39.8	72.3	6.5	12.5	22.9
All	189,249,510	−27.5	−39.5	−87.0	36.7	50.0	103.1	9.2	10.5	16.1

**Fig. 6:**
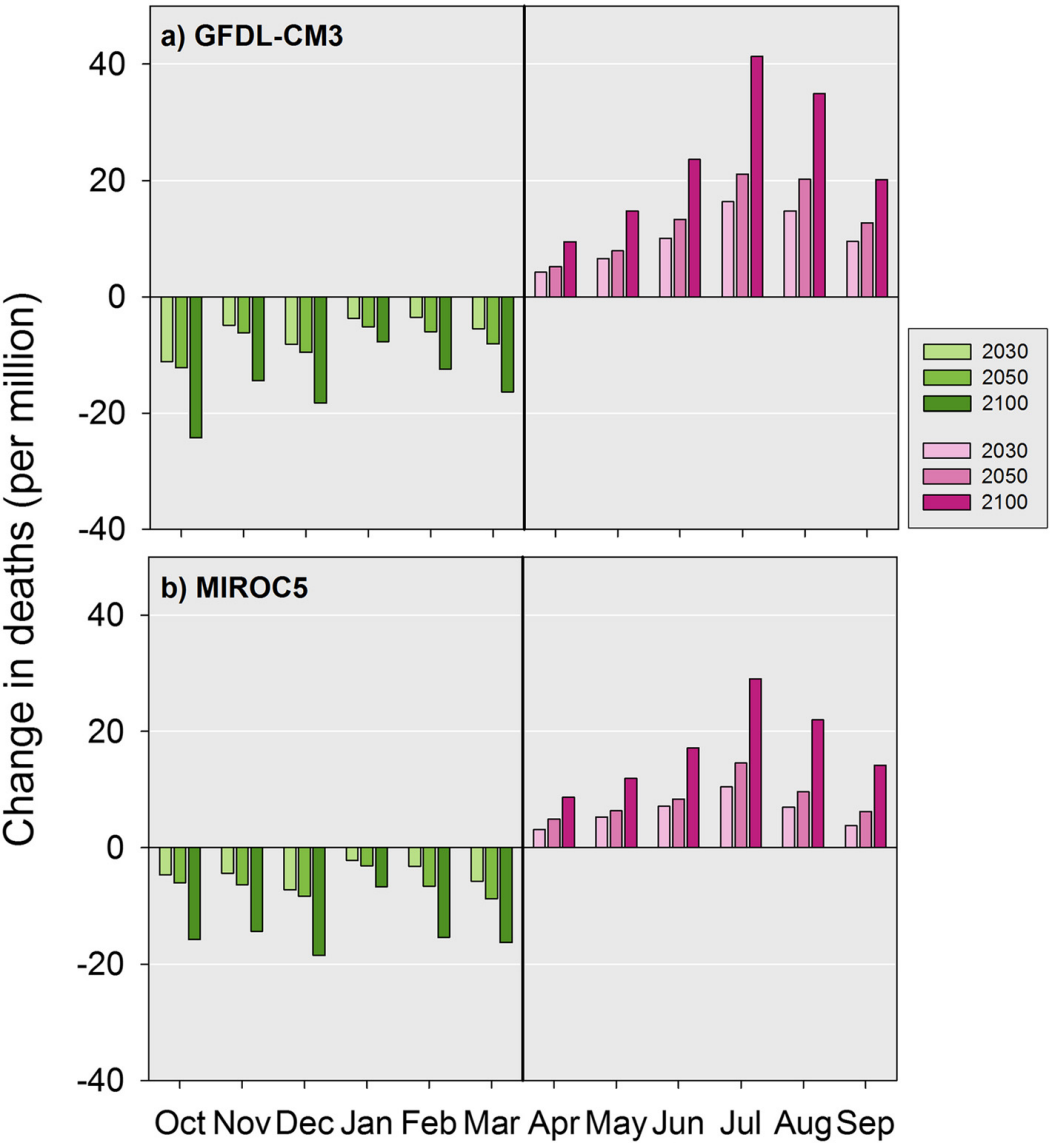
Projected change in premature temperature-attributable deaths by month per million study city residents for future reporting years relative to 1990 baseline for all study cities. *Legend:* This figure presents the projected change in premature temperature-attributable deaths per million study city residents for the future reporting years relative to the 1990 baseline across all study cities by month for both climate models

In both models, the magnitude of the increase in projected premature deaths in the hotter months exceeded the decreases in projected premature deaths in colder months across the designated reporting years. Specifically, for 2100, we projected a net increase of 9632 temperature-related premature deaths for the year across the study cities for the GFDL-CM3 model compared to 1990. This corresponded to 50.9 additional premature deaths per million persons in the study cities. For the MIROC5 model, we projected a net increase of 3042 temperature-related premature deaths across all cities, corresponding to an additional 16.1 premature deaths per million persons in the study cities compared to 1990 results (See Tables 2 and 3). Figure 6 disaggregates these annual changes to reflect the impact in terms of changes in the number of premature deaths per million persons by month for each model.

**Fig. 7:**
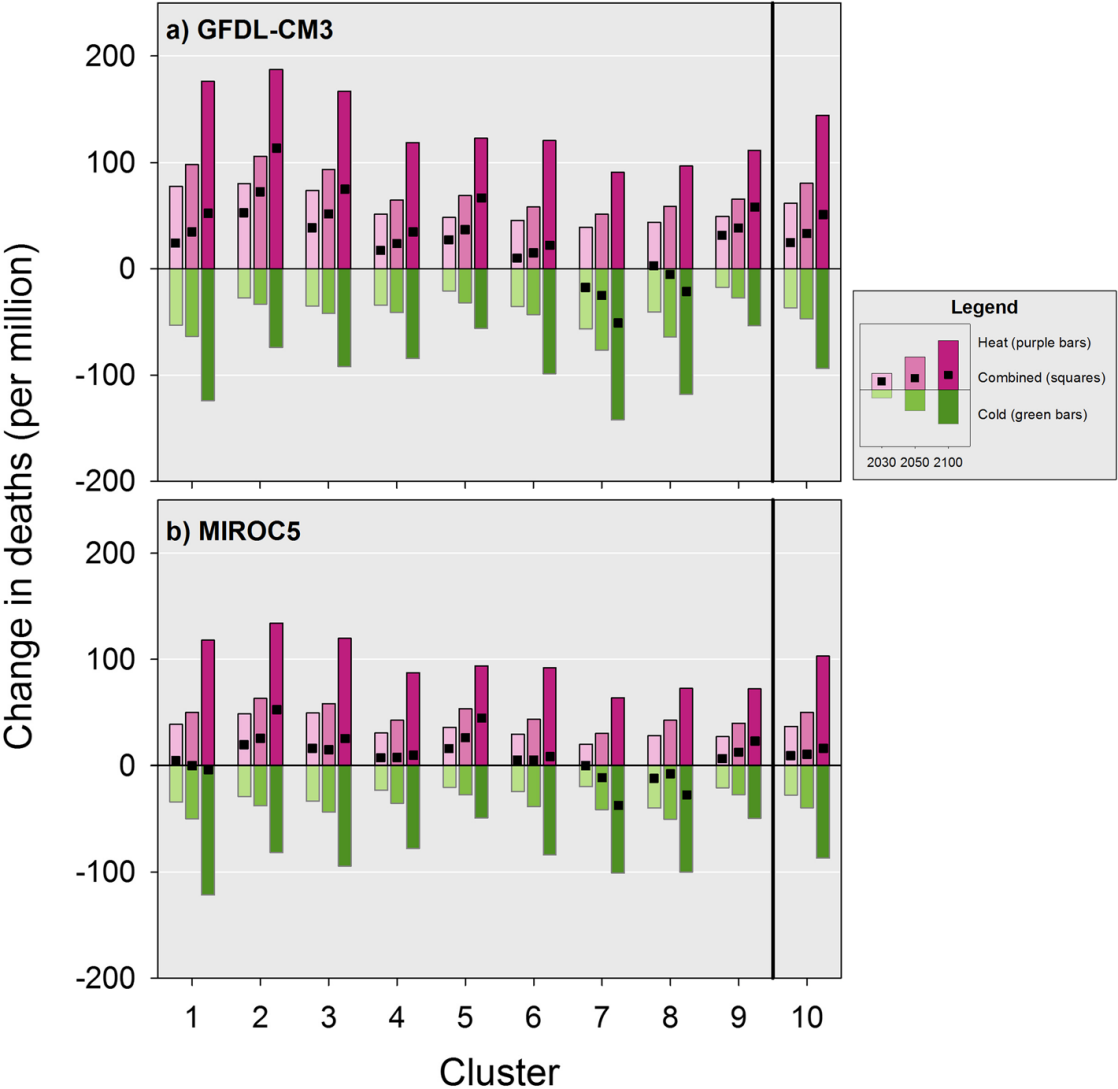
Combined effect of projected changes in premature temperature-attributable deaths from the hotter and colder months by individual cluster (1–9) and all clusters combined (10) in future reporting years relative to 1990 baseline. *Legend:* This figure presents the projected change in premature temperature-attributable deaths per million study residents by cluster and season for both climate models for the future reporting years relative to the 1990 baseline. Within a cluster results are presented from left to right for the 2030, 2050 and 2100 reporting years relative to 1990 baseline. Cumulative results across the clusters are presented as the results for cluster 10

We also examined future changes in premature deaths by climate cluster (see Tables 2, 3 and Fig. 7). Although most clusters followed the overall trend described above, in two clusters (7 and 8) characterized by warmer temperatures, net premature deaths decreased over time because reductions in colder months exceeded the increases during hotter months in both models. In addition, the MIROC5 model data projected that reductions in colder month premature deaths would roughly cancel out the increases in hotter months in clusters 1 and 6, in contrast to the results from the GFDL-CM3 model for these clusters where there is a clear net increase in premature deaths.

Additional file 2: Tables S2 and S3 provide corresponding projections for the colder and hotter months as well as the entire year, along with associated standard deviations, from the time slices for the different designated reporting years based on the GFDL-CM3 model’s climate data. Corresponding results based on the MIROC5 model’s data are provided in Additional file 2: Tables S4 and S5.

**Fig. 8:**
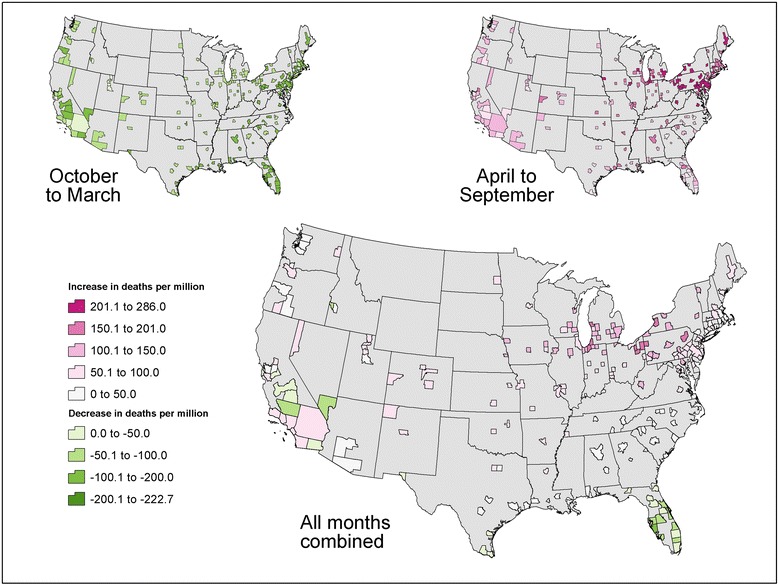
GFDL-CM3 projected combined change in premature temperature-attributable deaths per million study city residents in 2100 relative to 1990 baseline. *Legend:* This figure shows results for the change in premature temperature-attributable deaths per million study city residents in each study city in 2100 relative to the 1990 baseline based on GFDL-CM3 projections accounting for the cumulative effect of changes in premature mortality in both the hotter and colder months

**Fig. 9:**
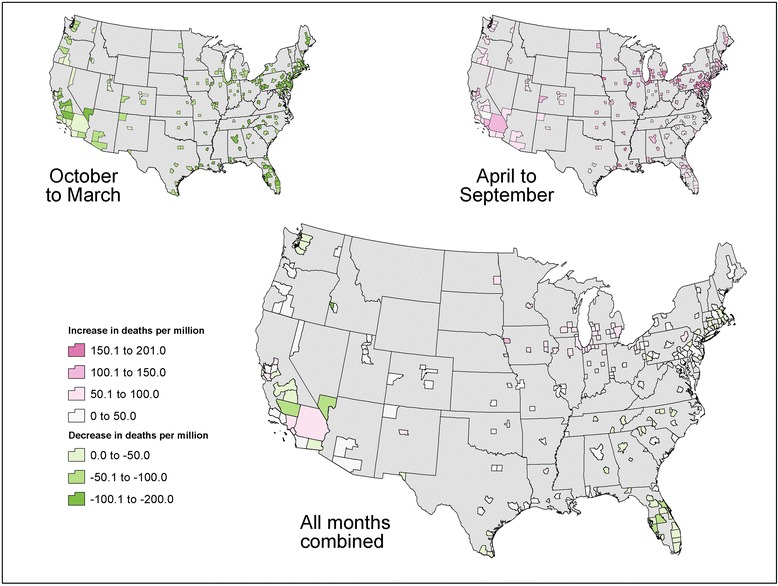
MIROC5 projected combined change in premature temperature-attributable deaths per million study city residents in 2100 relative to 1990 baseline. *Legend:* This figure shows results for the change in premature temperature-attributable deaths per million study city residents in each study city in 2100 relative to the 1990 baseline based on MIROC5 projections accounting for the cumulative effect of changes in premature mortality in both the hotter and colder months

Across the 209 cities, the combined heat- and cold-related changes in premature deaths per million study residents ranged from −100 to +181 from the GFDL-CM3 model and from −136 to +100 from the MIROC5 model. These city-specific results for the 2100 reporting year are reflected in Figs. 8 and 9 respectively.

For both climate models, projected increases in premature deaths in hotter months tended to be large in mid-western and northeastern cities. Changes in cold-related premature deaths showed less consistent spatial patterns. Mortality impacts across all months varied considerably over the country, with the largest increases in premature deaths projected in the upper mid-west and some northeastern cities and smaller increases to the South and West.

## Discussion

Using tailored relationships for the cities in each climate cluster that reflect observed temperature-mortality relationships in those locations and climate data for each study city, we found the net effect of climate change across our study locations would be to increase premature deaths in hotter months and decrease deaths in colder months. We also found the magnitude of these impacts increasing over time.

A key study feature was our use of continuous exposure-response curves that varied by cluster and month to develop premature mortality projections. This approach captures recent sensitivity to temperature while accounting for the timing and extent of the exposure within the year. This approach also enables comparisons between months that integrate the impact of variable warming and premature mortality risk by month. Based on these monthly results, we observe that projected warming in January would save relatively fewer lives than warming in the surrounding cold months. In contrast, projected July warming would result in more premature deaths than in other hot season months (see Fig. 6 for both results). It is also worth noting that exposure-response modeling based on short-term relationships between daily temperature and mortality may lead to some deaths being counted as temperature-related which were only moved forward, or “displaced,” by several days.

We also found evidence that temperature-mortality relationships have changed over the period from 1973 to 2006 (see Fig. 3), with increasing tolerance to the hottest temperatures. This might be explained by the acclimatization of populations over the course of 34 years. For example, this change could in part come from the increased penetration rate of air conditioning or heating in U.S. households over time. Although this shifting premature mortality-temperature relationship over time has been previously reported in the United States (e.g., [9, 24–26]), our study encompasses a longer time period across a larger number of cities.

For the country as a whole, we observed steady increases in projected changes in average net temperature-related mortality relative to 1990 in 2030, 2050 and 2100. These findings are consistent with results of other analyses that conclude climate change will increase temperature-attributable mortality over time (e.g., [5, 11, 27, 28]) although contrary results do exist in the literature [13].

However, details of our premature mortality projections differed between the climate models. While both models projected thousands of additional premature temperature-attributable deaths per year by 2100 relative to 1990, the GFDL-CM3 results were more than three times larger than the MIROC5 results. Additionally, the MIROC5 model data projected that reductions in colder month premature deaths would roughly cancel out the increases in hotter months in Clusters 1 and 6, in contrast to the results from the GFDL-CM3 where there is a net increase in temperature-attributable premature deaths in these clusters. This appears to reflect both relatively low sensitivities to heat and relatively high sensitivities to cold effects in these locations.

Collectively, these cluster results show a declining temperature-attributable mortality over time which raises the possibility that locations highly adapted to warmer temperatures (Cluster 6 includes portions of Texas, Louisiana, Alabama and Florida; Cluster 7 includes south Florida and Texas; and Cluster 8 includes southern California, Nevada and Arizona) could experience net premature mortality benefits from future warming. The difference between the two model projections of annual mortality in Cluster 1, which includes southern New England and the northern mid-Atlantic, is a result of the differences between the projections of temperature; MIROC5 projects lower increases in temperatures in this region, particularly in the hotter months, and therefore fewer heat-related mortalities. In short, in areas where cold temperatures are more exceptional than hot ones, the warming associated with climate change could produce a net health benefit with respect to temperature-attributable premature mortality.

At the same time, we found that in all regions, premature deaths during the hotter months are expected to increase. In contrast to these results, some other work (e.g., [10, 29]) project elevated premature mortality in Southern states despite a lower attributable risk, due to larger increases in frequency and duration of heat waves in that region. However, this research [10], while examining multiple definitions for heat waves, did not account for changes in mortality on hot days not identified as heat wave days.

Differences between our work and results in other research raise a broader issue of uncertainty and sensitivity to different assumptions/inputs. Collectively, this issue would extend to consideration of: alternative future climates, anticipated population growth and distribution patterns, alternative temperature-mortality relationships and direct consideration of the impact of future adaptation.

Clearly, our results and those from prior research (e.g., [30]) demonstrate that model selection influences results. While we only present the results of two climate models and therefore specific numerical results may be uncertain, the broad implications of the results (using a methodology that accounts for monthly variability in temperature changes and mortality response functions, as well as mortality responses for small changes in temperature, as well as extreme temperature events) add timely insight to the discussion of future climate impacts on premature mortality due to temperature effects.

More specifically, changes in the methods and data used to develop the temperature-mortality relationships would affect our results. For example, use of alternative regional definitions (e.g., [31, 32]) to assign cities to clusters would affect the subsequent meta-analyses and Bayesian adjustment that contribute to the final cluster-specific monthly relationships and projected mortality impacts at the city and cluster level. While completing the analysis with alternative cluster definitions could provide insight with respect to the importance of this choice we do not believe it would affect the sign and relative magnitude of the premature death results at the national level.

Likewise, our choice to develop month-specific temperature-mortality functions, the allocation of months to the hotter and colder periods and the use of slightly different models, in terms of same day or average lagged temperature exposure measures, affects our results. However, we believe the choices we made are consistent and supported with the available literature. Most importantly, we believe the framework we have developed directly incorporates and captures the current variation in temperature-mortality relationships over space and time that has been noted in recent research [2]. However, we believe these choices have little impact on the ultimate nature of the results in terms of sign, trends and orders of magnitude.

Our modeling framework does not explicitly incorporate a variable or explicit means to account for the full range and scope of potential future adaptation to climate change in general and temperature-attributable mortality risks more explicitly. Specifically, a key uncertainty in our results revolves around the extent to which the temperature-mortality relationships we incorporate will apply in the future.

Most explicitly, we based our future mortality projections on exposure-response slopes incorporating the most recent 10-year period of observed data (1997–2006) because of evidence these relationships have changed over time in our own results, consistent with results of other research (e.g., [9, 21, 24]) . We did not however extend these current observed trends to future periods. While improved adaptive responses over time could continue to reduce the mortality impact of temperature, there are likely limits to such adaptation as, for example, air conditioning penetration reaches 100 % or physiological tolerance reaches biological limits. In this context, it is important to note that our approach provides no constraint on the potential benefits that could accrue from future warming in cooler months and assumes that current relationships will hold for potentially warmer future extreme heat events. Some research has questioned these assumptions, particularly with respect to the assumption of reductions in future premature mortality in cooler months with a warming climate noting a number of influences that could contribute to or constrain future premature mortality reductions in cooler months [33]. Further exploration and incorporation of alternative adaptation assumptions remains an area of continued interest for future expansions of this research effort.

Finally, by not adjusting populations from their initial 2010 values, we are understating the magnitude of potential future impacts, all else being equal. The exact nature of this bias is uncertain though as exactly where a growing U.S. population will be located is critical to overall impacts given differences in temperature-mortality responses across the country [34].

## Conclusions

This study projected changes in premature deaths in 209 cities attributable to warming average temperatures from climate change, using month-specific relationships for different clusters of cities. Using projections from two climate models, our summary results show increases in premature temperature-attributable deaths in the U.S. over time; additional deaths during hotter months overwhelm reductions during colder months, while holding populations constant and making no direct adjustment for potential future adaptation. However, because there has been an observed increase in tolerance to high temperatures over time, as demonstrated in this and other works, there is an expectation that future mortality increases will be smaller than those in the results of this study.

However, we also identified a more nuanced picture at finer spatial scales. In our analysis, there were cities and clusters of cities projected to experience a net reduction in annual premature deaths attributable to temperature with continued climate change. We attributed this result primarily to continued reduction in premature deaths from temperatures in colder months in areas with relatively warm and consistent climates. While our research has not fully explored the potential impacts of these changes; it remains an important area for future research to address and to incorporate in future modeling efforts, along with an expanded consideration of data from additional climate models and population projections.

Still, our results suggest that climate change driven impacts on temperature alone will increase future health risks to an extent where there is the potential for at least thousands of additional premature deaths per year by the end of the century. This result highlights the importance of understanding how these risks vary now, and could change in the future, by location and time of year in order to help develop and improve strategies aimed at protecting public health.

## Additional files

Additional file 1: Table S1. Matching of counties to study cities. (DOCX 40 kb) Additional file 2: Table S2. Projected excess cold deaths (mean ± SD) for 209 cities, GFDL-CM3 model. **Table S3.** Projected excess heat deaths (mean ± SD) for 209 cities, GFDL-CM3 model. **Table S4.** Projected excess cold deaths (mean ± SD) for 209 cities, MIROC5 model. **Table S5.** Projected excess heat deaths (mean ± SD) for 209 cities, MIROC5 model. (DOCX 130 kb)

## Abbreviations

AF: Attributable fraction
BCCA: Bias-corrected constructed analogues
CMIP5: Coupled model intercomparison project phase 5
GFDL-CM3: Geophysical fluid dynamic laboratory–coupled physical model 3
ICD: International classification of diseases
ICLUS: Integrated climate and land use
MIROC5: Model for interdisciplinary research on climate
RR: Relative risks
