# Supplementary material for: Projections of temperature-attributable premature deaths in 209 U.S. cities using a cluster-based Poisson approach
Source: Environ Health. 2015 Nov 4;14:85. doi: 10.1186/s12940-015-0071-2 (PMC4632409; doi:10.1186/s12940-015-0071-2)
Supplement: Additional file 1: Table S1. — Matching of counties to study cities. (DOCX 40 kb) [file 12940_2015_71_MOESM1_ESM.docx]

| **Table S1. Matching of counties to study cities** | | | |
| --- | --- | --- | --- |
| **Study city name** | **County** | **State** | **Study cluster** |
| Allentown | Lehigh | Pennsylvania | 1 |
| Allentown | Northampton | Pennsylvania | 1 |
| Annandale | Fairfax | Virginia | 1 |
| Atlantic City | Atlantic | New Jersey | 1 |
| Baltimore | Baltimore | Maryland | 1 |
| Baltimore | Baltimore City | Maryland | 1 |
| Barnstable | Barnstable | Massachusetts | 1 |
| Bergen | Bergen | New Jersey | 1 |
| Bergen | Passaic | New Jersey | 1 |
| Boston | Middlesex | Massachusetts | 1 |
| Boston | Norfolk | Massachusetts | 1 |
| Boston | Suffolk | Massachusetts | 1 |
| Carlisle | Cumberland | Pennsylvania | 1 |
| Dover | Kent | Delaware | 1 |
| Elizabeth | Union | New Jersey | 1 |
| Gettysburg | Adams | Pennsylvania | 1 |
| Harrisburg | Dauphin | Pennsylvania | 1 |
| Hartford | Hartford | Connecticut | 1 |
| Jersey City | Hudson | New Jersey | 1 |
| Lancaster | Lancaster | Pennsylvania | 1 |
| Marlboro | Prince George’s | Maryland | 1 |
| Middlesex | Middlesex | New Jersey | 1 |
| Monmouth | Ocean | New Jersey | 1 |
| Nassau | Nassau | New York | 1 |
| Nassau | Suffolk | New York | 1 |
| Newark | Essex | New Jersey | 1 |
| Newark | Morris | New Jersey | 1 |
| Newburgh | Orange | New York | 1 |
| New Haven | New Haven | Connecticut | 1 |
| New London | New London | Connecticut | 1 |
| New York | Bronx | New York | 1 |
| New York | Kings | New York | 1 |
| New York | New York | New York | 1 |
| New York | Queens | New York | 1 |
| New York | Richmond | New York | 1 |
| Philadelphia | Burlington | New Jersey | 1 |
| Philadelphia | Camden | New Jersey | 1 |
| Philadelphia | Gloucester | New Jersey | 1 |
| Philadelphia | Bucks | Pennsylvania | 1 |
| Philadelphia | Delaware | Pennsylvania | 1 |
| Philadelphia | Montgomery | Pennsylvania | 1 |
| Philadelphia | Philadelphia | Pennsylvania | 1 |
| Plymouth | Plymouth | Massachusetts | 1 |
| Providence | Kent | Rhode Island | 1 |
| Providence | Providence | Rhode Island | 1 |
| Reading | Berks | Pennsylvania | 1 |
| Richmond | Chesterfield | Virginia | 1 |
| Richmond | Henrico | Virginia | 1 |
| Richmond | Richmond City | Virginia | 1 |
| Rockville | Montgomery | Maryland | 1 |
| Essex | Essex | Massachusetts | 1 |
| Springfield | Hampden | Massachusetts | 1 |
| Stamford | Fairfield | Connecticut | 1 |
| Trenton | Mercer | New Jersey | 1 |
| Washington, DC | District of Columbia | District of Columbia | 1 |
| Washington, DC | Arlington | Virginia | 1 |
| Wilmington | New Castle | Delaware | 1 |
| York | York | Pennsylvania | 1 |
| Akron | Summit | Ohio | 2 |
| Albany | Albany | New York | 2 |
| Anchorage | Anchorage | Alaska | 2 |
| Ann Arbor | Washtenaw | Michigan | 2 |
| Bangor | Penobscot | Maine | 2 |
| Bath | Steuben | New York | 2 |
| Boulder | Boulder | Colorado | 2 |
| Buffalo | Erie | New York | 2 |
| Burlington | Chittenden | Vermont | 2 |
| Canton | Stark | Ohio | 2 |
| Cedar Rapids | Linn | Iowa | 2 |
| Chicago | Cook | Illinois | 2 |
| Chicago | DuPage | Illinois | 2 |
| Chicago | Lake | Illinois | 2 |
| Colorado Springs | El Paso | Colorado | 2 |
| Davenport | Rock Island | Illinois | 2 |
| Davenport | Scott | Iowa | 2 |
| Denver | Adams | Colorado | 2 |
| Denver | Denver | Colorado | 2 |
| Denver | Jefferson | Colorado | 2 |
| Des Moines | Polk | Iowa | 2 |
| Detroit | Macomb | Michigan | 2 |
| Detroit | Oakland | Michigan | 2 |
| Detroit | Wayne | Michigan | 2 |
| Dodge | Dodge | Wisconsin | 2 |
| Elkhart | Elkhart | Indiana | 2 |
| Erie | Erie | Pennsylvania | 2 |
| Fargo | Cass | North Dakota | 2 |
| Flint | Genesee | Michigan | 2 |
| Fort Wayne | Allen | Indiana | 2 |
| Gary | Lake | Indiana | 2 |
| Grand Haven | Ottawa | Michigan | 2 |
| Grand Rapids | Kent | Michigan | 2 |
| Green Bay | Brown | Wisconsin | 2 |
| Holland | Allegan | Michigan | 2 |
| Iowa City | Johnson | Iowa | 2 |
| Kalamazoo | Kalamazoo | Michigan | 2 |
| Kenosha | Kenosha | Wisconsin | 2 |
| Lansing | Ingham | Michigan | 2 |
| LaPorte | LaPorte | Indiana | 2 |
| Logan | Cache | Utah | 2 |
| Madison | Dane | Wisconsin | 2 |
| Mercer | Mercer | Pennsylvania | 2 |
| Milwaukee | Milwaukee | Wisconsin | 2 |
| Milwaukee | Waukesha | Wisconsin | 2 |
| Minneapolis | Hennepin | Minnesota | 2 |
| Minneapolis | Ramsey | Minnesota | 2 |
| Muskegon | Muskegon | Michigan | 2 |
| Nashua | Hillsborough | New Hampshire | 2 |
| Niles | Berrien | Michigan | 2 |
| Omaha | Douglas | Nebraska | 2 |
| Ottawa | La Salle | Illinois | 2 |
| Portage | Porter | Indiana | 2 |
| Portland | Cumberland | Maine | 2 |
| Rochester | Monroe | New York | 2 |
| South Bend | St. Joseph | Indiana | 2 |
| State College | Centre | Pennsylvania | 2 |
| Scranton | Lackawanna | Pennsylvania | 2 |
| Scranton | Luzerne | Pennsylvania | 2 |
| Sioux City | Woodbury | Iowa | 2 |
| Toledo | Lucas | Ohio | 2 |
| Worcester | Worcester | Massachusetts | 2 |
| Youngstown | Mahoning | Ohio | 2 |
| Youngstown | Trumbull | Ohio | 2 |
| Charleston | Kanawha | West Virginia | 3 |
| Cincinnati | Hamilton | Ohio | 3 |
| Cleveland | Cuyahoga | Ohio | 3 |
| Cleveland | Lake | Ohio | 3 |
| Cleveland | Lorain | Ohio | 3 |
| Columbus | Franklin | Ohio | 3 |
| Dayton | Montgomery | Ohio | 3 |
| Evansville | Vanderburgh | Indiana | 3 |
| Greensburg | Westmoreland | Pennsylvania | 3 |
| Indianapolis | Marion | Indiana | 3 |
| Kansas | Johnson | Kansas | 3 |
| Kansas | Wyandotte | Kansas | 3 |
| Kansas | Clay | Missouri | 3 |
| Kansas | Jackson | Missouri | 3 |
| Lafayette | Tippecanoe | Indiana | 3 |
| Louisville | Jefferson | Kentucky | 3 |
| Madison | Madison | Illinois | 3 |
| Middletown | Butler | Ohio | 3 |
| Muncie | Delaware | Indiana | 3 |
| Pittsburgh | Allegheny | Pennsylvania | 3 |
| Saint Charles | Saint Charles | Missouri | 3 |
| Saint Clair | Saint Clair | Illinois | 3 |
| Springfield | Greene | Missouri | 3 |
| Steubenville | Jefferson | Ohio | 3 |
| Steubenville | Brooke | West Virginia | 3 |
| Steubenville | Hancock | West Virginia | 3 |
| St. Louis | Jefferson | Missouri | 3 |
| St. Louis | Saint Louis | Missouri | 3 |
| St. Louis | St. Louis City | Missouri | 3 |
| Terra Haute | Vigo | Indiana | 3 |
| Topeka | Shawnee | Kansas | 3 |
| Washington | Washington | Pennsylvania | 3 |
| Wichita | Sedgwick | Kansas | 3 |
| Atlanta | Clayton | Georgia | 4 |
| Atlanta | Cobb | Georgia | 4 |
| Atlanta | DeKalb | Georgia | 4 |
| Atlanta | Fulton | Georgia | 4 |
| Atlanta | Gwinnett | Georgia | 4 |
| Augusta | Richmond | Georgia | 4 |
| Birmingham | Blount | Alabama | 4 |
| Birmingham | Jefferson | Alabama | 4 |
| Birmingham | Shelby | Alabama | 4 |
| Birmingham | Walker | Alabama | 4 |
| Charlotte | Mecklenburg | North Carolina | 4 |
| Charleston | Charleston | South Carolina | 4 |
| Chattanooga | Hamilton | Tennessee | 4 |
| Columbia | Lexington | South Carolina | 4 |
| Columbia | Richland | South Carolina | 4 |
| Dallas | Dallas | Texas | 4 |
| Durham | Durham | North Carolina | 4 |
| Fayetteville | Cumberland | North Carolina | 4 |
| Fort Worth | Tarrant | Texas | 4 |
| Greensboro | Guilford | North Carolina | 4 |
| Greenville | Greenville | South Carolina | 4 |
| Hickory | Catawba | North Carolina | 4 |
| Knoxville | Blount | Tennessee | 4 |
| Knoxville | Knox | Tennessee | 4 |
| Little Rock | Pulaski | Arkansas | 4 |
| Macon | Bibb | Georgia | 4 |
| Memphis | Shelby | Tennessee | 4 |
| Monroe | Ouachita | Louisiana | 4 |
| Myrtle Beach | Horry | South Carolina | 4 |
| Nashville | Davidson | Tennessee | 4 |
| Norfolk | York | Virginia | 4 |
| Norfolk | Chesapeake City | Virginia | 4 |
| Norfolk | Hampton | Virginia | 4 |
| Norfolk | Newport News City | Virginia | 4 |
| Norfolk | Norfolk City | Virginia | 4 |
| Norfolk | Poquoson | Virginia | 4 |
| Norfolk | Portsmouth | Virginia | 4 |
| Norfolk | Virginia Beach City | Virginia | 4 |
| Oklahoma | Oklahoma | Oklahoma | 4 |
| Raleigh | Wake | North Carolina | 4 |
| Spartanburg | Spartanburg | South Carolina | 4 |
| Tulsa | Tulsa | Oklahoma | 4 |
| Winston | Forsyth | North Carolina | 4 |
| Anaheim | Orange | California | 5 |
| Eugene | Lane | Oregon | 5 |
| Everett | Snohomish | Washington | 5 |
| Los Angeles | Los Angeles | California | 5 |
| Oakland | Alameda | California | 5 |
| Oakland | Contra Costa | California | 5 |
| Portland | Clackamas | Oregon | 5 |
| Portland | Multnomah | Oregon | 5 |
| Portland | Washington | Oregon | 5 |
| Riverside | Riverside | California | 5 |
| Riverside | San Bernardino | California | 5 |
| Sacramento | Sacramento | California | 5 |
| Santa Barbara | Santa Barbara | California | 5 |
| San Diego | San Diego | California | 5 |
| Seattle | King | Washington | 5 |
| San Francisco | San Francisco | California | 5 |
| San Francisco | San Mateo | California | 5 |
| San Jose | Santa Clara | California | 5 |
| Stockton | San Joaquin | California | 5 |
| Tacoma | Pierce | Washington | 5 |
| Vancouver | Clark | Washington | 5 |
| Ventura | Ventura | California | 5 |
| Austin | Travis | Texas | 6 |
| Baton Rouge | East Baton Rouge | Louisiana | 6 |
| Baton Rouge | West Baton Rouge | Louisiana | 6 |
| Gainesville | Alachua | Florida | 6 |
| Houston | Harris | Texas | 6 |
| Jacksonville | Duval | Florida | 6 |
| Lafayette | Lafayette | Louisiana | 6 |
| Lake Charles | Calcasieu | Louisiana | 6 |
| Mobile | Mobile | Alabama | 6 |
| Montgomery | Montgomery | Alabama | 6 |
| New Orleans | Jefferson | Louisiana | 6 |
| New Orleans | Orleans | Louisiana | 6 |
| Ocala | Marion | Florida | 6 |
| Port Arthur | Jefferson | Texas | 6 |
| Pensacola | Escambia | Florida | 6 |
| San Antonio | Bexar | Texas | 6 |
| Tallahassee | Leon | Florida | 6 |
| Brownsville | Cameron | Texas | 7 |
| Corpus Christi | Nueces | Texas | 7 |
| Daytona Beach | Volusia | Florida | 7 |
| Fort Lauderdale | Broward | Florida | 7 |
| Fort Myers | Lee | Florida | 7 |
| Fort Pierce | Martin | Florida | 7 |
| Fort Pierce | St. Lucie | Florida | 7 |
| Honolulu | Honolulu | Hawaii | 7 |
| Lakeland | Polk | Florida | 7 |
| McAllen | Hidalgo | Texas | 7 |
| Melbourne | Brevard | Florida | 7 |
| Miami | Miami-Dade | Florida | 7 |
| Orlando | Orange | Florida | 7 |
| Orlando | Seminole | Florida | 7 |
| Palm Beach | Palm Beach | Florida | 7 |
| Sarasota | Manatee | Florida | 7 |
| Sarasota | Sarasota | Florida | 7 |
| St. Petersburg | Pinellas | Florida | 7 |
| Tampa | Hillsborough | Florida | 7 |
| Bakersfield | Kern | California | 8 |
| El Centro | Imperial | California | 8 |
| El Paso | El Paso | Texas | 8 |
| Fresno | Fresno | California | 8 |
| Las Vegas | Clark | Nevada | 8 |
| Modesto | Stanislaus | California | 8 |
| Phoenix | Maricopa | Arizona | 8 |
| Tucson | Pima | Arizona | 8 |
| Visalia | Tulare | California | 8 |
| Albuquerque | Bernalillo | New Mexico | 9 |
| Aztec | San Juan | New Mexico | 9 |
| Davis | Davis | Utah | 9 |
| Grand Junction | Mesa | Colorado | 9 |
| Klamath | Klamath | Oregon | 9 |
| Medford | Jackson | Oregon | 9 |
| Boise | Ada | Idaho | 9 |
| Nampa | Canyon | Idaho | 9 |
| Provo | Utah | Utah | 9 |
| Reno | Washoe | Nevada | 9 |
| Salt Lake City | Salt Lake | Utah | 9 |
| Spokane | Spokane | Washington | 9 |
| Weber | Weber | Utah | 9 |
