# Supplementary material for: Projections of temperature-attributable premature deaths in 209 U.S. cities using a cluster-based Poisson approach
Source: Environ Health. 2015 Nov 4;14:85. doi: 10.1186/s12940-015-0071-2 (PMC4632409; doi:10.1186/s12940-015-0071-2)
Supplement: Additional file 2: Table S2. — Projected excess cold deaths (mean ± SD) for 209 cities, GFDL-CM3 model. Table S3. Projected excess heat deaths (mean ± SD) for 209 cities, GFDL-CM3 model. Table S4. Projected excess cold deaths (mean ± SD) for 209 cities, MIROC5 model. Table S5. Projected excess heat deaths (mean ± SD) for 209 cities, MIROC5 model. (DOCX 130 kb) [file 12940_2015_71_MOESM2_ESM.docx]

**Supplemental Material**

**Title:** Projections of temperature-attributable deaths in 209 U.S. cities using a cluster-based Poisson approach

**Authors**: ^1^Joel D. Schwartz, ^1^Mihye Lee, ^2^Patrick L. Kinney, ^2^Suijia Yang, ^3^David Mills, ^4^Marcus Sarofim; ^3^Russell Jones, ^3^Richard Streeter, ^3^Alexis St. Juliana, ^3^Jennifer Peers, ^5^Radley M. Horton

**Affiliations:** ^1^Harvard School of Public Health, Department of Environmental Health, Department of Epidemiology, Harvard University, Boston, MA, USA; ^2^Columbia Climate and Health Program, Columbia University, New York, NY, USA; ^3^Stratus Consulting Inc., Boulder, CO, USA; ^4^Climate Change Division (6207-J), U.S. Environmental Protection Agency, Washington, DC, USA; ^5^Center for Climate Systems Research, Columbia University, New York, NY, USA

## Corresponding Author:

## *Name*: David Mills

*Email*: [dmills@stratusconsulting.com](mailto:dmills@stratusconsulting.com)

*Tel*: 303 381 8248

*Express mail address*: Stratus Consulting, 1881 Ninth Street, Suite 201, Boulder, CO 80302

| **Table S2. Projected excess cold deaths (mean ± SD) for 209 cities, GFDL-CM3 model** | | | | | | |
| --- | --- | --- | --- | --- | --- | --- |
|  |  |  | **Cold deaths (October–March)** | | | |
| **City** | **Cluster** | **Population (2010)** | **1990** | **2030** | **2050** | **2100** |
| Akron (OH) | 2 | 547,578 | -16.3 ± 12.11 | -34.45 ± 16.71 | -38.05 ± 16.03 | -64.11 ± 14.37 |
| Albany (NY) | 2 | 303,379 | 0.42 ± 6.02 | -12.71 ± 7.7 | -15.19 ± 7.35 | -29.61 ± 6.64 |
| Albuquerque (NM) | 9 | 652,540 | -19.39 ± 6.9 | -33.62 ± 8.28 | -38.13 ± 10.51 | -58.71 ± 11.47 |
| Allentown (PA) | 1 | 634,504 | 22.72 ± 18.25 | -14.88 ± 23.81 | -22.43 ± 22.2 | -67.58 ± 20.88 |
| Anaheim (CA) | 5 | 3,135,981 | -221.89 ± 70.91 | -289.03 ± 51.22 | -316.63 ± 44.94 | -382.64 ± 44.21 |
| Annandale (VA) | 1 | 1,350,383 | -45.16 ± 26.13 | -86.43 ± 24.95 | -92.2 ± 23.79 | -136.84 ± 24.57 |
| Ann Arbor (MI) | 2 | 369,089 | -1.54 ± 5.46 | -10.39 ± 7.59 | -11.9 ± 7.84 | -24.15 ± 6.28 |
| Atlanta (GA) | 4 | 3,982,479 | 16.99 ± 62.22 | -106.47 ± 64.95 | -130.55 ± 65.02 | -276.74 ± 78.53 |
| Atlantic City (NJ) | 1 | 301,495 | -8.29 ± 11.48 | -27.91 ± 12.49 | -31.23 ± 11.47 | -53.64 ± 11.71 |
| Aztec (NM) | 9 | 134,161 | 2.98 ± 2.07 | -0.5 ± 1.67 | -1.43 ± 2.04 | -5.2 ± 2.01 |
| Augusta (GA) | 4 | 200,965 | -8.32 ± 6.3 | -17.38 ± 5.04 | -19.47 ± 4.65 | -29.5 ± 5.94 |
| Austin (TX) | 6 | 1,186,535 | 26.04 ± 21.81 | 2.85 ± 24.68 | -2.74 ± 26.12 | -43.88 ± 19.39 |
| Bakersfield (CA) | 8 | 833,820 | 10.76 ± 31.06 | -17.44 ± 29.85 | -40.01 ± 25.61 | -88.1 ± 27.35 |
| Baltimore (MD) | 1 | 1,460,369 | -73.57 ± 54.5 | -166.55 ± 58.62 | -181.52 ± 54.89 | -288.24 ± 53.42 |
| Bangor (ME) | 2 | 149,052 | 6.27 ± 3.4 | -1.49 ± 4.21 | -3.12 ± 3.7 | -12.19 ± 3.16 |
| Barnstable (MA) | 1 | 231,855 | 2.92 ± 9.7 | -16.03 ± 10.31 | -19.54 ± 9.13 | -40.81 ± 9.04 |
| Bath (NY) | 2 | 94,238 | -0.04 ± 2.04 | -3.53 ± 2.7 | -4.19 ± 2.58 | -8.79 ± 2.25 |
| Paterson (NJ) | 1 | 1,532,376 | -5.12 ± 45.28 | -83.66 ± 47.65 | -98.63 ± 45.12 | -189.89 ± 44.54 |
| Birmingham (AL) | 4 | 993,979 | -28.4 ± 28.41 | -78.7 ± 27.68 | -86.94 ± 28.72 | -149.92 ± 34.18 |
| Boston (MA) | 1 | 2,888,305 | 61.37 ± 68.98 | -107.84 ± 93.45 | -142.06 ± 87.57 | -327.73 ± 85.4 |
| Boulder (CO) | 2 | 328,237 | -11.03 ± 5.56 | -16.86 ± 3.8 | -19.65 ± 5.32 | -29.7 ± 4.51 |
| Baton Rouge (LA) | 6 | 452,533 | 17.51 ± 13.52 | 0.33 ± 14.02 | -2.82 ± 15.61 | -29.8 ± 12.43 |
| Brownsville (TX) | 7 | 495,290 | 16.38 ± 10.86 | 1.46 ± 13.41 | -3.83 ± 15.78 | -26.45 ± 12.1 |
| Buffalo (NY) | 2 | 940,553 | -12.37 ± 18.72 | -51.59 ± 31.35 | -57.41 ± 29.61 | -106.73 ± 25.09 |
| Burlington (VT) | 2 | 156,597 | 4.15 ± 2.88 | -1.83 ± 3.67 | -3.06 ± 3.56 | -9.99 ± 2.8 |
| El Centro (CA) | 8 | 176,113 | -16.84 ± 6 | -23.83 ± 4.58 | -27.2 ± 3.97 | -35.2 ± 4.29 |
| Canton (OH) | 2 | 375,352 | -9.77 ± 8.38 | -21.95 ± 11.43 | -24.41 ± 10.95 | -42.04 ± 9.77 |
| Carlisle (PA) | 1 | 235,540 | 1.39 ± 7.7 | -13.53 ± 9.23 | -16.41 ± 8.8 | -34.16 ± 8.01 |
| Cedar Rapids (IA) | 2 | 208,038 | 3.17 ± 4.48 | -1.69 ± 5.95 | -3 ± 5.79 | -10.93 ± 5.55 |
| Charlotte (NC) | 4 | 975,223 | 13.51 ± 19.9 | -23.67 ± 20.7 | -30.66 ± 19.22 | -68.03 ± 21.98 |
| Charleston (SC) | 4 | 345,379 | -26.68 ± 7.02 | -38.6 ± 7.35 | -41.86 ± 6.86 | -56.55 ± 8.54 |
| Charleston (WV) | 3 | 183,151 | -2.58 ± 5.24 | -11.32 ± 7.2 | -12.87 ± 6.45 | -24 ± 6.73 |
| Chattanooga (TN) | 4 | 332,777 | 12.78 ± 9.27 | -3.01 ± 9.75 | -5.9 ± 10 | -25.39 ± 11.73 |
| Chicago (IL) | 2 | 7,213,360 | -251.44 ± 142.81 | -434.32 ± 171.71 | -466.97 ± 172.82 | -728.98 ± 161.63 |
| Cincinnati (OH) | 3 | 855,143 | 6.26 ± 18.1 | -22.45 ± 25.8 | -27.84 ± 24.85 | -67.71 ± 23.44 |
| Cleveland (OH) | 3 | 1,951,224 | 59.86 ± 46.21 | -16.4 ± 71.65 | -31.59 ± 67.12 | -136.17 ± 57.01 |
| Colorado Springs (CO) | 2 | 626,053 | -8.75 ± 7.98 | -19.43 ± 10.48 | -25.66 ± 13.98 | -48.72 ± 12.71 |
| Columbia (SC) | 4 | 639,401 | -19.75 ± 16.75 | -44.22 ± 14.6 | -50.71 ± 14.13 | -81.06 ± 18.8 |
| Columbus (OH) | 3 | 1,229,096 | 30.25 ± 25.17 | -4.45 ± 32.21 | -11.96 ± 31.36 | -58.97 ± 26.89 |
| Corpus Christi (TX) | 7 | 344,734 | 40.32 ± 11.85 | 25.15 ± 13.41 | 22.26 ± 14.78 | -4.03 ± 11.68 |
| Dallas (TX) | 4 | 2,697,303 | -125.85 ± 42.65 | -178.33 ± 55.63 | -186.97 ± 52.71 | -277.49 ± 54.37 |
| Davenport (IA) | 2 | 314,174 | -1.71 ± 7.2 | -10.74 ± 10.31 | -12.97 ± 10.11 | -27.54 ± 9.7 |
| Layton (UT) | 9 | 297,737 | 4.15 ± 3.33 | 0.91 ± 2.9 | -1.21 ± 2.83 | -7.21 ± 2.57 |
| Dayton (OH) | 3 | 550,109 | 18.7 ± 13.33 | -2.59 ± 19.11 | -6.82 ± 18.63 | -36.44 ± 17.55 |
| Daytona Beach (FL) | 7 | 509,402 | 80.76 ± 30.01 | 37.92 ± 35.08 | 24.37 ± 30.35 | -20.8 ± 21.45 |
| Denver (CO) | 2 | 1,738,073 | -37.56 ± 21.98 | -63.82 ± 26.32 | -77.94 ± 34.8 | -132.43 ± 31.03 |
| Des Moines (IA) | 2 | 452,715 | -1 ± 8.65 | -9.99 ± 12.4 | -12.65 ± 11.54 | -27.61 ± 11.24 |
| Detroit (MI) | 2 | 4,125,648 | -122.57 ± 87.76 | -254.94 ± 117.3 | -279.27 ± 122.09 | -470.65 ± 98.26 |
| Beaver Dam (WI) | 2 | 89,463 | 2.99 ± 2.09 | 0.59 ± 2.65 | -0.1 ± 2.76 | -4.03 ± 2.12 |
| Dover (DE) | 1 | 152,592 | -7.88 ± 5.23 | -18.13 ± 6.34 | -19.75 ± 5.86 | -31.19 ± 5.81 |
| Durham (NC) | 4 | 262,292 | 7.23 ± 5.42 | -3.56 ± 6.18 | -5.29 ± 5.5 | -15.62 ± 5.96 |
| Elizabeth (NJ) | 1 | 561,109 | -11.49 ± 15.68 | -40.24 ± 17.69 | -45.76 ± 16.75 | -79.32 ± 16.3 |
| Elkhart (IN) | 2 | 206,000 | -6.28 ± 4.02 | -11.63 ± 4.97 | -12.45 ± 5.05 | -20.26 ± 4.57 |
| El Paso (TX) | 8 | 820,495 | 53.27 ± 22.46 | 21.44 ± 14.51 | 6.54 ± 19.76 | -44.2 ± 16.36 |
| Erie (PA) | 2 | 287,899 | -8.59 ± 7.45 | -19.19 ± 8.79 | -20.87 ± 8.54 | -35.04 ± 7.05 |
| Eugene (OR) | 5 | 337,986 | 64.72 ± 11.72 | 51.66 ± 14.82 | 43.45 ± 12.85 | 29.1 ± 10.55 |
| Evansville (IN) | 3 | 174,247 | -2.86 ± 4.15 | -9.56 ± 5.98 | -10.7 ± 5.89 | -20.35 ± 6.23 |
| Everett (WA) | 5 | 705,799 | 95.83 ± 22.99 | 67.43 ± 24.7 | 54.04 ± 19.88 | 30.64 ± 17.34 |
| Fargo (ND) | 2 | 139,598 | 12.37 ± 3.58 | 10.18 ± 3.13 | 9.04 ± 3.49 | 4.13 ± 3.05 |
| Fayetteville (NC) | 4 | 317,492 | -3.4 ± 7.38 | -18.02 ± 8.34 | -20.44 ± 7.58 | -34.93 ± 8.78 |
| Flint (MI) | 2 | 451,035 | 1.41 ± 9.89 | -12.48 ± 12.85 | -15.09 ± 13.35 | -35.74 ± 10.63 |
| Fresno (CA) | 8 | 970,014 | 57.78 ± 30.82 | 21.61 ± 30.35 | -2 ± 24.16 | -50.4 ± 21.05 |
| Fort Lauderdale (FL) | 7 | 1,833,639 | -190.19 ± 68.6 | -285.7 ± 82.6 | -321.04 ± 64.95 | -428.99 ± 52.44 |
| Fort Myers (FL) | 7 | 616,755 | -10.32 ± 29.24 | -51.07 ± 33.8 | -65.72 ± 27.16 | -110.26 ± 19.88 |
| Fort Pierce (FL) | 7 | 410,505 | 0.38 ± 21.25 | -27.66 ± 23.99 | -37.66 ± 19.09 | -67.97 ± 14.12 |
| Fort Wayne (IN) | 2 | 356,260 | -6.42 ± 6.81 | -16.37 ± 9.24 | -17.91 ± 9.31 | -32.18 ± 8.76 |
| Fort Worth (TX) | 4 | 1,868,738 | -87.57 ± 30.98 | -131.31 ± 44.55 | -138.01 ± 42.23 | -212.89 ± 42.84 |
| Gary (IN) | 2 | 509,249 | -20.3 ± 10.13 | -33.21 ± 12.54 | -35.52 ± 12.54 | -54.78 ± 11.96 |
| Gainesville (FL) | 6 | 246,289 | -4.5 ± 7.62 | -15.32 ± 8.2 | -18.92 ± 7.55 | -32.38 ± 6 |
| Gettysburg (PA) | 1 | 103,888 | -1 ± 4.23 | -7.42 ± 4.11 | -8.54 ± 3.91 | -15.86 ± 3.74 |
| Grand Haven (MI) | 2 | 278,837 | -0.41 ± 3.76 | -5.36 ± 4.98 | -6.36 ± 5.13 | -14.39 ± 3.95 |
| Grand Junction (CO) | 9 | 134,793 | 2.95 ± 2.9 | -1.06 ± 2.6 | -2.66 ± 2.72 | -7.65 ± 2.46 |
| Grand Rapids (MI) | 2 | 655,332 | 2.05 ± 10.38 | -12.95 ± 14.11 | -15.58 ± 14.8 | -37.88 ± 12.16 |
| Green Bay (WI) | 2 | 270,814 | 11.07 ± 5.53 | 4.87 ± 6.81 | 2.86 ± 7.28 | -7.59 ± 4.97 |
| Greensboro (NC) | 4 | 468,766 | 28.06 ± 12.81 | 5.02 ± 14.18 | 0.57 ± 13.15 | -23.14 ± 13.88 |
| Greensburg (PA) | 3 | 353,508 | 8.76 ± 13.53 | -9.69 ± 15.13 | -12.76 ± 13.56 | -35.64 ± 12.55 |
| Greenville (SC) | 4 | 439,607 | -2.3 ± 16.62 | -25.62 ± 11.29 | -29.96 ± 10.85 | -53.64 ± 13.43 |
| Harrisburg (PA) | 1 | 268,917 | 1.67 ± 9.14 | -16.17 ± 11 | -19.64 ± 10.5 | -40.81 ± 9.51 |
| Hartford (CT) | 1 | 892,660 | 33.33 ± 30.02 | -23.17 ± 31.13 | -33.82 ± 28.94 | -95.7 ± 28.46 |
| Hickory (NC) | 4 | 160,102 | 8.33 ± 4.33 | -0.02 ± 4.45 | -1.69 ± 4.16 | -10.15 ± 4.82 |
| Holland (MI) | 2 | 117,602 | -0.01 ± 2.14 | -2.69 ± 2.84 | -3.27 ± 2.94 | -7.85 ± 2.24 |
| Houston (TX) | 6 | 3,968,385 | -64.15 ± 94.14 | -169.38 ± 87.77 | -188.69 ± 100.79 | -375.24 ± 74.05 |
| Indianapolis (IN) | 3 | 928,593 | 25.41 ± 17.55 | -1.84 ± 24.93 | -6.41 ± 25.08 | -45.55 ± 24.26 |
| Iowa City (IA) | 2 | 132,779 | 1.49 ± 2.72 | -0.83 ± 2.95 | -1.49 ± 2.91 | -5.41 ± 2.77 |
| Jacksonville (FL) | 6 | 888,355 | -49.75 ± 45.57 | -100.33 ± 34.57 | -113.47 ± 30.97 | -171.8 ± 28.27 |
| Jersey City (NJ) | 1 | 714,944 | 11.36 ± 17.81 | -18.26 ± 17.87 | -24.69 ± 16.67 | -59.58 ± 16.59 |
| Kalamazoo (MI) | 2 | 256,426 | -3.46 ± 4.7 | -10.36 ± 6.76 | -11.6 ± 7.03 | -22.23 ± 5.82 |
| Kansas City (KS) | 3 | 1,607,438 | -7.86 ± 30.78 | -45.41 ± 51.68 | -55.14 ± 48.37 | -119.3 ± 49.88 |
| Kenosha (WI) | 2 | 171,483 | -0.87 ± 3.56 | -6.38 ± 3.97 | -7.17 ± 4 | -13.29 ± 3.43 |
| Klamath Falls (OR) | 9 | 63,287 | 3.17 ± 1.34 | 1.91 ± 1.52 | 1.09 ± 1.44 | -0.69 ± 1.22 |
| Knoxville (TN) | 4 | 549,539 | 32.87 ± 13.83 | 4.62 ± 16.73 | -1.37 ± 15.79 | -34.29 ± 19.22 |
| Lafayette (IN) | 3 | 174,427 | 6.89 ± 4.03 | 1.4 ± 5.26 | 0.53 ± 5.24 | -7.34 ± 4.98 |
| Lafayette (LA) | 6 | 207,716 | 4.7 ± 6.62 | -3.79 ± 7.11 | -5.35 ± 7.81 | -18.75 ± 6.12 |
| Lakeland (FL) | 7 | 572,942 | 16.28 ± 41.36 | -24.94 ± 33.73 | -38.34 ± 28.17 | -80.73 ± 18.99 |
| Lancaster (PA) | 1 | 519,229 | 0.05 ± 17.86 | -30.37 ± 18.98 | -36.08 ± 17.91 | -72.56 ± 16.41 |
| Lansing (MI) | 2 | 314,338 | 2.27 ± 5.24 | -5.25 ± 7.25 | -6.65 ± 7.63 | -18.12 ± 6.11 |
| La Porte (IN) | 2 | 108,383 | -2.44 ± 2.43 | -5.67 ± 3.24 | -6.2 ± 3.31 | -11.22 ± 3.06 |
| Las Vegas (NV) | 8 | 2,074,364 | 68.92 ± 115.33 | -68 ± 111.7 | -151.73 ± 92.17 | -310.9 ± 102.79 |
| Lake Charles (LA) | 6 | 189,618 | 0.53 ± 11.07 | -8.94 ± 7.62 | -10.58 ± 8.48 | -24.69 ± 6.47 |
| Logan (UT) | 2 | 111,182 | 1.24 ± 1.82 | -0.18 ± 1.36 | -1.27 ± 1.3 | -3.91 ± 1.06 |
| Los Angeles (CA) | 5 | 10,559,243 | -563.69 ± 140.76 | -734.28 ± 167.55 | -820.37 ± 148.63 | -1061.77 ± 175.9 |
| Louisville (KY) | 3 | 738,978 | -17.37 ± 16.87 | -43.99 ± 21.66 | -48.55 ± 21.12 | -85.47 ± 21.85 |
| Little Rock (AR) | 4 | 380,611 | 2.69 ± 8.77 | -9.36 ± 10.84 | -12.19 ± 11.06 | -30.74 ± 11.62 |
| Macon (GA) | 4 | 157,307 | -9.09 ± 3.3 | -16.18 ± 3.87 | -17.62 ± 3.84 | -26.08 ± 4.59 |
| Madison (IL) | 3 | 273,407 | -6.39 ± 9.52 | -17.76 ± 10.94 | -19.56 ± 10.33 | -35.98 ± 11.85 |
| Madison (WI) | 2 | 571,173 | 13.4 ± 10.68 | 0.89 ± 13.06 | -2.52 ± 13.58 | -22.27 ± 10.63 |
| Upper Marlboro (MD) | 1 | 895,794 | -37.93 ± 21.42 | -79.43 ± 25.52 | -85.3 ± 23.74 | -130.93 ± 24.64 |
| Mcallen (TX) | 7 | 762,535 | 22.4 ± 17.18 | -2.7 ± 19.87 | -7.49 ± 21.47 | -49.16 ± 17.44 |
| Medford (OR) | 9 | 197,576 | -6.52 ± 3.44 | -9.47 ± 4.21 | -11.45 ± 4.02 | -17.01 ± 4.06 |
| Melbourne (FL) | 7 | 547,450 | 11.25 ± 27.69 | -27.53 ± 35.31 | -41.17 ± 28.88 | -83.62 ± 21.24 |
| Memphis (TN) | 4 | 954,592 | -3.8 ± 19.4 | -39.12 ± 28.69 | -46.34 ± 30.42 | -96.95 ± 31.76 |
| Mercer (PA) | 2 | 112,377 | -1.88 ± 3.17 | -6.44 ± 4.05 | -7.24 ± 3.85 | -13.64 ± 3.28 |
| Boise City (ID) | 9 | 386,522 | 5.62 ± 7.27 | 0.17 ± 7.16 | -3.05 ± 6.44 | -12.01 ± 5.06 |
| Miami (FL) | 7 | 3,099,526 | -265.85 ± 87.81 | -399.38 ± 109.54 | -455.56 ± 87.51 | -618.1 ± 70.98 |
| Middlesex (NJ) | 1 | 881,321 | -7.94 ± 24.96 | -49.16 ± 25.6 | -57.36 ± 24.32 | -106.33 ± 23.98 |
| Middletown (OH) | 3 | 383,546 | 2.88 ± 7.98 | -8.73 ± 10.4 | -10.86 ± 9.94 | -26.89 ± 9.55 |
| Milwaukee (WI) | 2 | 1,357,450 | 3.58 ± 29.04 | -33.05 ± 35.7 | -40.49 ± 36.35 | -95.79 ± 30.9 |
| Minneapolis (MN) | 2 | 1,743,595 | 60.87 ± 30.86 | 29.87 ± 33.44 | 20.08 ± 35.18 | -32.4 ± 30.01 |
| Mobile (AL) | 6 | 417,866 | 10 ± 18.59 | -9.73 ± 13.6 | -14.62 ± 15.64 | -40.4 ± 12.48 |
| Modesto (CA) | 8 | 573,131 | 30.33 ± 23.95 | 8.29 ± 20.02 | -5.92 ± 16.47 | -34.68 ± 11.32 |
| Toms River (NJ) | 1 | 587,588 | -26.85 ± 26.87 | -69.47 ± 25.41 | -77.36 ± 24.17 | -125.56 ± 23.69 |
| Monroe (LA) | 4 | 153,883 | -8.85 ± 3.83 | -14.24 ± 4.6 | -15.14 ± 4.71 | -23.73 ± 4.94 |
| Montgomery (AL) | 6 | 228,548 | 15.39 ± 8.22 | 2.58 ± 7.79 | -0.34 ± 8.46 | -15.8 ± 8.02 |
| Muncie (IN) | 3 | 114,470 | 8.58 ± 4.8 | 3.74 ± 4.44 | 2.95 ± 4.48 | -3.64 ± 3.99 |
| Muskegon (MI) | 2 | 177,808 | -0.5 ± 3.61 | -5.25 ± 4.74 | -6.2 ± 4.89 | -13.86 ± 3.77 |
| Myrtle Beach (SC) | 4 | 254,638 | -18.05 ± 7.71 | -30.34 ± 8.11 | -32.77 ± 7.32 | -46.51 ± 8.53 |
| Nampa (ID) | 9 | 185,267 | 2.89 ± 12.3 | -10.89 ± 21.16 | -17.58 ± 14.87 | -28.38 ± 11.98 |
| Nashua (NH) | 2 | 437,603 | -11.74 ± 9.04 | -29 ± 9.37 | -32 ± 8.41 | -49.94 ± 8.23 |
| Nashville (TN) | 4 | 652,834 | 25.24 ± 14.02 | 4.5 ± 16.76 | -0.45 ± 17.82 | -30.22 ± 19.09 |
| Melville (NY) | 1 | 3,000,255 | 0.45 ± 102.36 | -164.42 ± 94.64 | -196.56 ± 88.04 | -382.97 ± 88.93 |
| Newark (NJ) | 1 | 1,365,331 | -30.2 ± 38.73 | -101.48 ± 43.87 | -115.02 ± 41.43 | -198.49 ± 40.2 |
| Newburgh (NY) | 1 | 419,658 | 20.12 ± 10.44 | -1.61 ± 13.66 | -6.32 ± 12.99 | -32.64 ± 12.32 |
| New Haven (CT) | 1 | 872,769 | -3.89 ± 30.65 | -58.7 ± 31.54 | -69.38 ± 29.46 | -131.5 ± 28.8 |
| New London (CT) | 1 | 269,894 | 1.14 ± 7.95 | -14.56 ± 8.85 | -17.49 ± 8.24 | -34.88 ± 8.08 |
| Niles (MI) | 2 | 162,189 | -4.2 ± 3.99 | -9.56 ± 5.26 | -10.43 ± 5.37 | -18.55 ± 4.93 |
| Norfolk (VA) | 4 | 1,514,213 | 25.96 ± 28.46 | -24.16 ± 30.37 | -34.95 ± 28.51 | -83.85 ± 31.47 |
| New Orleans (LA) | 6 | 945,703 | -24.95 ± 40.89 | -68.41 ± 34.15 | -76.1 ± 36.56 | -138.22 ± 31.62 |
| New York (NY) | 1 | 10,396,046 | 157.69 ± 264.61 | -290.19 ± 268.83 | -387.37 ± 252.66 | -916.38 ± 258.91 |
| Oakland (CA) | 5 | 2,651,188 | -37.49 ± 51.44 | -83.98 ± 54.9 | -115.89 ± 45.67 | -172.06 ± 30.58 |
| Ocala (FL) | 6 | 327,455 | -19.82 ± 19.61 | -44.96 ± 18.84 | -52.98 ± 17.26 | -84.06 ± 13.69 |
| Oklahoma City (OK) | 4 | 732,376 | 36.17 ± 18.38 | 14.66 ± 25.96 | 8.79 ± 25.41 | -26.35 ± 23.22 |
| Omaha (NE) | 2 | 525,046 | -2.45 ± 10.76 | -11.71 ± 14.12 | -14.91 ± 13.52 | -32.53 ± 13.19 |
| Orlando (FL) | 7 | 1,617,589 | 80.18 ± 54.91 | 0.19 ± 65.16 | -26.2 ± 55.29 | -109.99 ± 38.04 |
| Ottawa (IL) | 2 | 110,661 | -1.09 ± 1.96 | -4.09 ± 2.93 | -4.73 ± 2.99 | -9.14 ± 2.85 |
| Port Arthur (TX) | 6 | 262,282 | 2.16 ± 7.93 | -9.53 ± 9.08 | -11.55 ± 10.35 | -29.01 ± 7.62 |
| Palm Beach (FL) | 7 | 1,363,061 | -88.18 ± 72.8 | -165.39 ± 75.67 | -195.34 ± 58.57 | -284.91 ± 47.52 |
| Pensacola (FL) | 6 | 311,251 | 7.16 ± 11.91 | -6.2 ± 10.55 | -9.84 ± 11.31 | -28.32 ± 9.3 |
| Philadelphia (PA) | 1 | 4,875,946 | -91.66 ± 174.13 | -392.99 ± 189.06 | -443.31 ± 174.68 | -785.73 ± 172.68 |
| Phoenix (AZ) | 8 | 3,828,993 | -308.01 ± 77.33 | -437.12 ± 79.5 | -497.13 ± 82.01 | -664.79 ± 82.72 |
| Pittsburgh (PA) | 3 | 1,237,627 | 55.14 ± 40.36 | -7.65 ± 53.54 | -18.86 ± 48.02 | -98.98 ± 44.64 |
| Plymouth (MA) | 1 | 508,851 | 21.21 ± 12.83 | -8.93 ± 17.29 | -14.91 ± 15.7 | -49.15 ± 15.27 |
| Portage (IN) | 2 | 169,889 | -6.97 ± 3.51 | -11.44 ± 4.35 | -12.22 ± 4.34 | -18.93 ± 4.17 |
| Portland (OR) | 5 | 1,627,866 | 224.37 ± 43.03 | 167.51 ± 59.12 | 137.39 ± 50.7 | 84.13 ± 40.71 |
| Portland (ME) | 2 | 283,360 | 2.39 ± 5.32 | -9.58 ± 6.76 | -12.05 ± 6.17 | -25.85 ± 5.79 |
| Providence (RI) | 1 | 837,540 | 27.97 ± 26.26 | -24.4 ± 29.92 | -34.8 ± 27.25 | -94.11 ± 26.41 |
| Provo (UT) | 9 | 509,040 | 6.43 ± 6.02 | -0.68 ± 6.09 | -4.6 ± 4.91 | -13.94 ± 4.56 |
| Raleigh (NC) | 4 | 874,071 | 20.52 ± 14.99 | -9.37 ± 16.75 | -14.07 ± 14.89 | -42.35 ± 16.32 |
| Reading (PA) | 1 | 424,666 | 5.64 ± 16.79 | -21.81 ± 16.82 | -27.01 ± 15.88 | -59.44 ± 14.6 |
| Reno (NV) | 9 | 470,501 | 3.12 ± 8.15 | -3.78 ± 8.57 | -8.4 ± 8.57 | -21.02 ± 8.5 |
| Richmond (VA) | 1 | 863,737 | -79.64 ± 25.69 | -128.55 ± 29.43 | -134.85 ± 27.97 | -186.41 ± 31.91 |
| Riverside (CA) | 5 | 4,249,734 | -66.69 ± 78.56 | -140.38 ± 69.23 | -172.87 ± 59.6 | -256.31 ± 55.72 |
| Rochester (NY) | 2 | 785,278 | -6.21 ± 14.55 | -34.13 ± 22.15 | -39.2 ± 21.2 | -76.33 ± 16.85 |
| Rockville (MD) | 1 | 993,900 | -62.4 ± 29.27 | -103.11 ± 23.68 | -108.57 ± 22.99 | -151.54 ± 23.76 |
| Sacramento (CA) | 5 | 1,443,068 | 36.43 ± 33.82 | 4.02 ± 39.48 | -17.9 ± 30.82 | -62.01 ± 18.77 |
| Essex (MA) | 1 | 756,210 | 26.65 ± 22.83 | -21.24 ± 26.22 | -30.92 ± 24.63 | -83.2 ± 24.09 |
| Salt Lake City (UT) | 9 | 1,063,702 | 9.3 ± 10.97 | -6.8 ± 13.95 | -16.62 ± 10.48 | -38.16 ± 9.38 |
| San Antonio (TX) | 6 | 1,685,188 | -5.77 ± 56.65 | -54.98 ± 46.09 | -65.04 ± 48.4 | -168.87 ± 38.88 |
| Sarasota (FL) | 7 | 705,040 | 17.89 ± 48.6 | -44.23 ± 49.11 | -64.99 ± 41.68 | -134.78 ± 28.14 |
| Santa Barbara (CA) | 5 | 425,999 | -15.23 ± 7.26 | -23.66 ± 9.13 | -29.13 ± 7.05 | -41.55 ± 6.63 |
| South Bend (IN) | 2 | 282,297 | -6.3 ± 6.12 | -14.39 ± 7.92 | -15.72 ± 8.14 | -28 ± 7.46 |
| St. Charles (MO) | 3 | 377,645 | -3.63 ± 7.44 | -14.25 ± 10.33 | -15.9 ± 9.79 | -31.67 ± 11.3 |
| East St. Louis (IL) | 3 | 266,433 | -5.24 ± 8.33 | -15 ± 9.67 | -16.48 ± 9.15 | -31.13 ± 10.6 |
| State College (PA) | 2 | 165,012 | -3.34 ± 4.2 | -9.57 ± 3.92 | -10.51 ± 3.68 | -17.59 ± 3.26 |
| Scranton (PA) | 2 | 503,640 | -15.45 ± 13.63 | -40.48 ± 16.86 | -44.96 ± 15.72 | -75.77 ± 13.94 |
| San Diego (CA) | 5 | 3,076,373 | -218.67 ± 65.05 | -282.96 ± 45.36 | -312.11 ± 40.67 | -386.38 ± 38.58 |
| Seattle (WA) | 5 | 2,095,098 | 241.99 ± 46.2 | 189.6 ± 58.61 | 159.32 ± 49.09 | 113.67 ± 36.58 |
| San Francisco (CA) | 5 | 1,474,044 | -57.42 ± 28.53 | -92.06 ± 37.69 | -116.81 ± 29.9 | -157.9 ± 24.66 |
| Sioux City (IA) | 2 | 104,608 | 2.57 ± 3 | 0.2 ± 3.69 | -0.56 ± 3.77 | -5.45 ± 3.61 |
| San Jose (CA) | 5 | 1,773,974 | -65.99 ± 26.96 | -97.65 ± 30.24 | -114.77 ± 22.65 | -152.04 ± 17.25 |
| Spartanburg (SC) | 4 | 290,717 | 3.76 ± 8.48 | -11.36 ± 8.38 | -14.56 ± 7.91 | -31.78 ± 9.68 |
| St. Petersburg (FL) | 7 | 947,997 | -3.01 ± 65.4 | -88.86 ± 64.73 | -117.95 ± 55.94 | -214.11 ± 39.15 |
| Spokane (WA) | 9 | 455,457 | 18.35 ± 7.51 | 11.57 ± 10.23 | 7.28 ± 9.65 | -5.61 ± 7.8 |
| Springfield (MA) | 1 | 470,398 | 31.91 ± 15.31 | -2.76 ± 19.17 | -9.53 ± 18.1 | -46.94 ± 17.45 |
| Springfield (MO) | 3 | 262,664 | -4.01 ± 5.56 | -12.3 ± 9.14 | -13.84 ± 8.62 | -27.16 ± 10.11 |
| Stamford (CT) | 1 | 929,629 | 27.67 ± 27.43 | -22.74 ± 28.88 | -33.52 ± 27.96 | -91.61 ± 27.33 |
| Steubenville (OH) | 3 | 108,793 | 4.16 ± 4.53 | -1.2 ± 4.81 | -2.3 ± 4.39 | -9.47 ± 3.76 |
| St. Louis (MO) | 3 | 1,620,561 | -40.95 ± 45.31 | -101.43 ± 56.28 | -110.94 ± 52.21 | -192.3 ± 58.89 |
| Stockton (CA) | 5 | 726,034 | 15.51 ± 18.3 | -3.98 ± 18.19 | -15.14 ± 13.02 | -36.59 ± 7.47 |
| Tacoma (WA) | 5 | 801,618 | 125.5 ± 29.25 | 95.17 ± 29.78 | 79.16 ± 24.4 | 53.12 ± 21.63 |
| Tallahassee (FL) | 6 | 286,424 | 7.52 ± 8.45 | -4.33 ± 7.57 | -7.29 ± 7.76 | -21.96 ± 6.9 |
| Tampa (FL) | 7 | 1,322,129 | 70.86 ± 56.38 | -5.79 ± 58.81 | -31.58 ± 50.78 | -118.88 ± 34.35 |
| Terre Haute (IN) | 3 | 101,706 | 3.24 ± 3.3 | -0.83 ± 4.16 | -1.64 ± 4.1 | -7.63 ± 4.21 |
| Toledo (OH) | 2 | 464,712 | -10.63 ± 9.01 | -25.83 ± 13.11 | -28.6 ± 13.28 | -49.25 ± 11.01 |
| Topeka (KS) | 3 | 177,127 | 2.97 ± 4.23 | -1.28 ± 6.5 | -2.51 ± 6.14 | -10.63 ± 6.55 |
| Trenton (NJ) | 1 | 392,619 | -3.6 ± 11.63 | -22.54 ± 12.34 | -26.46 ± 11.66 | -49.91 ± 11.38 |
| Tucson (AZ) | 8 | 980,969 | -2.04 ± 23.67 | -34.18 ± 18.65 | -49.23 ± 21.93 | -97.21 ± 22.65 |
| Tulsa (OK) | 4 | 603,879 | 28.28 ± 16.72 | 5.89 ± 23.92 | 0.41 ± 24.14 | -34.02 ± 23.12 |
| Vancouver (WA) | 5 | 489,556 | 73.21 ± 13.87 | 55.07 ± 19.21 | 44.99 ± 16.3 | 27.84 ± 13.54 |
| Ventura (CA) | 5 | 905,979 | -44.57 ± 21.78 | -62.38 ± 15.44 | -70.9 ± 12.11 | -91.14 ± 10.9 |
| Visalia (CA) | 8 | 478,651 | 25 ± 14 | 8.66 ± 13.66 | -2.02 ± 10.64 | -23.87 ± 9.94 |
| Washington (PA) | 3 | 196,082 | 8.91 ± 7.85 | -0.42 ± 8.29 | -2.36 ± 7.62 | -14.83 ± 6.62 |
| Washington (DC) | 1 | 810,015 | -57.91 ± 20.73 | -99.54 ± 25.82 | -105.72 ± 25.02 | -152.22 ± 25.94 |
| Ogden (UT) | 9 | 224,311 | 1.84 ± 4.14 | -1.99 ± 3.35 | -3.94 ± 3.37 | -10.41 ± 3.42 |
| Wichita (KS) | 3 | 506,519 | -3.76 ± 10.21 | -17.63 ± 16.64 | -21.3 ± 16.58 | -45.76 ± 16.98 |
| Wilmington (DE) | 1 | 556,014 | -10.85 ± 15.91 | -40.08 ± 18.45 | -45.13 ± 17.09 | -78.33 ± 17.02 |
| Winston-Salem (NC) | 4 | 340,276 | 11.01 ± 9.61 | -6.54 ± 11.03 | -10.02 ± 10.21 | -28.63 ± 10.87 |
| Worcester (MA) | 2 | 804,868 | 0.73 ± 14.82 | -29.85 ± 17.4 | -35.65 ± 15.99 | -69.58 ± 15.26 |
| York (PA) | 1 | 419,797 | 4.26 ± 12.44 | -18.68 ± 14.25 | -23.13 ± 13.55 | -50.51 ± 12.17 |
| Youngstown (OH) | 2 | 453,322 | -9.6 ± 11.57 | -26.71 ± 15.28 | -29.93 ± 14.88 | -53.58 ± 12.39 |

| **Table S3. Projected excess heat deaths (mean ± SD) for 209 cities, GFDL-CM3 model** | | | | | | |
| --- | --- | --- | --- | --- | --- | --- |
|  |  |  | **Heat deaths (April–September)** | | | |
| **City** | **Cluster** | **Population (2010)** | **1990** | **2030** | **2050** | **2100** |
| Akron (OH) | 2 | 547,578 | 28.72 ± 16.86 | 82.29 ± 25.81 | 101.23 ± 21.92 | 160.12 ± 19.17 |
| Albany (NY) | 2 | 303,379 | 9.07 ± 6.44 | 36.53 ± 12.76 | 45.7 ± 10.86 | 73.72 ± 10.65 |
| Albuquerque (NM) | 9 | 652,540 | 51.07 ± 12.38 | 102.24 ± 15.89 | 109.6 ± 19.28 | 140.3 ± 21.98 |
| Allentown (PA) | 1 | 634,504 | 29.17 ± 14.89 | 89.17 ± 25.9 | 104.2 ± 22.72 | 165.8 ± 20.3 |
| Anaheim (CA) | 5 | 3,135,981 | 363.22 ± 162.37 | 545.89 ± 86.44 | 612.98 ± 61.57 | 771.81 ± 52.95 |
| Annandale (VA) | 1 | 1,350,383 | 92.35 ± 28.83 | 165.15 ± 27.48 | 183.61 ± 25.24 | 253.26 ± 23.73 |
| Ann Arbor (MI) | 2 | 369,089 | 13.72 ± 7.82 | 39.38 ± 10.55 | 47.99 ± 9.73 | 75.81 ± 7.72 |
| Atlanta (GA) | 4 | 3,982,479 | 19.38 ± 51.83 | 161.65 ± 67.24 | 208.64 ± 71.01 | 388.01 ± 69.16 |
| Atlantic City (NJ) | 1 | 301,495 | 20.44 ± 7.88 | 48.6 ± 12.68 | 56.39 ± 11.12 | 86.16 ± 12.5 |
| Aztec (NM) | 9 | 134,161 | 3.25 ± 2.67 | 12.2 ± 2.73 | 14.55 ± 3.24 | 21.58 ± 2.93 |
| Augusta (GA) | 4 | 200,965 | 6.91 ± 4.58 | 18.37 ± 5.45 | 21.63 ± 4.75 | 33.52 ± 4.79 |
| Austin (TX) | 6 | 1,186,535 | 23.95 ± 15.74 | 70.82 ± 22.87 | 79.87 ± 24.31 | 138.75 ± 16.81 |
| Bakersfield (CA) | 8 | 833,820 | -13.27 ± 13.47 | 3.75 ± 13.61 | 12.48 ± 12.4 | 36.38 ± 11.51 |
| Baltimore (MD) | 1 | 1,460,369 | 180.7 ± 59.09 | 349.66 ± 67.41 | 388.99 ± 59.12 | 551.75 ± 50.98 |
| Bangor (ME) | 2 | 149,052 | -4 ± 2.41 | 7.58 ± 6.74 | 13.02 ± 6.62 | 29.3 ± 7.24 |
| Barnstable (MA) | 1 | 231,855 | -1.01 ± 7.29 | 18.76 ± 11.91 | 24.67 ± 10.16 | 48.76 ± 10.84 |
| Bath (NY) | 2 | 94,238 | 1.52 ± 2.5 | 10.09 ± 4.28 | 13.14 ± 3.96 | 23.02 ± 3.28 |
| Paterson (NJ) | 1 | 1,532,376 | 103.23 ± 43.3 | 228.16 ± 52.79 | 259.38 ± 44.74 | 377.01 ± 43.01 |
| Birmingham (AL) | 4 | 993,979 | 28.5 ± 24.75 | 85.93 ± 29.21 | 107.67 ± 32.77 | 177.93 ± 26.39 |
| Boston (MA) | 1 | 2,888,305 | 68 ± 52.8 | 258.11 ± 97.09 | 322.27 ± 87.23 | 536.51 ± 81.83 |
| Boulder (CO) | 2 | 328,237 | 19.34 ± 10.57 | 40.53 ± 7.51 | 47.37 ± 8.22 | 66.65 ± 8.22 |
| Baton Rouge (LA) | 6 | 452,533 | 2.07 ± 5.49 | 19.34 ± 8.71 | 25.09 ± 8.42 | 55.23 ± 6.62 |
| Brownsville (TX) | 7 | 495,290 | 5.8 ± 3.94 | 17.84 ± 4.99 | 21.53 ± 4.43 | 33.67 ± 4.49 |
| Buffalo (NY) | 2 | 940,553 | 19.16 ± 24.83 | 120.11 ± 43.78 | 154.21 ± 41.2 | 261.95 ± 28.95 |
| Burlington (VT) | 2 | 156,597 | 1.72 ± 2.53 | 12.95 ± 5.13 | 17.62 ± 4.76 | 32.62 ± 4.71 |
| El Centro (CA) | 8 | 176,113 | 13.32 ± 4.02 | 21 ± 3.24 | 23.66 ± 2.21 | 29.74 ± 1.53 |
| Canton (OH) | 2 | 375,352 | 18.61 ± 11.22 | 55.19 ± 17.64 | 68.11 ± 14.99 | 108.71 ± 13.07 |
| Carlisle (PA) | 1 | 235,540 | 19.64 ± 9.05 | 45.1 ± 10.98 | 51.31 ± 9.46 | 76.87 ± 7.88 |
| Cedar Rapids (IA) | 2 | 208,038 | 11.94 ± 5.62 | 30.69 ± 7.27 | 34.01 ± 5.97 | 49.1 ± 6.31 |
| Charlotte (NC) | 4 | 975,223 | 2.73 ± 13.63 | 44.7 ± 18.32 | 55.04 ± 17.63 | 99.85 ± 19.52 |
| Charleston (SC) | 4 | 345,379 | 14.95 ± 4.96 | 31.45 ± 7.98 | 36.59 ± 6.94 | 53.84 ± 6.02 |
| Charleston (WV) | 3 | 183,151 | 1.17 ± 4.33 | 19.57 ± 8.12 | 25.46 ± 7.96 | 43.01 ± 7.39 |
| Chattanooga (TN) | 4 | 332,777 | 1.6 ± 8.2 | 23.17 ± 9.87 | 30.06 ± 10.31 | 54.84 ± 10.02 |
| Chicago (IL) | 2 | 7,213,360 | 599.51 ± 213.88 | 1145.3 ± 219.83 | 1300.11 ± 201.74 | 1772.45 ± 151.62 |
| Cincinnati (OH) | 3 | 855,143 | 13.14 ± 15.62 | 71.29 ± 25.85 | 88.15 ± 23.87 | 143.68 ± 19.89 |
| Cleveland (OH) | 3 | 1,951,224 | 5.1 ± 48.34 | 158.92 ± 68.66 | 211.84 ± 63.28 | 388.01 ± 51.4 |
| Colorado Springs (CO) | 2 | 626,053 | 3.62 ± 9.74 | 39.96 ± 14.86 | 51.57 ± 18.34 | 89.34 ± 20.23 |
| Columbia (SC) | 4 | 639,401 | 21.22 ± 12.26 | 53.8 ± 16.35 | 63.42 ± 14.4 | 98.95 ± 14.38 |
| Columbus (OH) | 3 | 1,229,096 | -0.95 ± 22.68 | 69.72 ± 30.89 | 91.31 ± 28.55 | 166.45 ± 25.07 |
| Corpus Christi (TX) | 7 | 344,734 | 2.7 ± 3.07 | 17.48 ± 4.72 | 20.87 ± 4.03 | 36.82 ± 3.61 |
| Dallas (TX) | 4 | 2,697,303 | 184.86 ± 50.8 | 309.12 ± 68.06 | 325.9 ± 72.82 | 445.12 ± 54.55 |
| Davenport (IA) | 2 | 314,174 | 30.23 ± 10.58 | 63.7 ± 12.79 | 70.58 ± 11.06 | 98.58 ± 10.27 |
| Layton (UT) | 9 | 297,737 | 5.12 ± 4.36 | 17.33 ± 4.64 | 21.98 ± 4.39 | 32.87 ± 4.62 |
| Dayton (OH) | 3 | 550,109 | 0.97 ± 13.19 | 39.54 ± 18.13 | 51.26 ± 16.63 | 91.12 ± 14.15 |
| Daytona Beach (FL) | 7 | 509,402 | -21.12 ± 8.61 | 6.08 ± 9.27 | 13.58 ± 7.94 | 40.6 ± 8.54 |
| Denver (CO) | 2 | 1,738,073 | 60.96 ± 32.08 | 166.83 ± 40.12 | 199.49 ± 44.18 | 299.57 ± 46.47 |
| Des Moines (IA) | 2 | 452,715 | 40.61 ± 11.97 | 77.87 ± 15.85 | 83.62 ± 11.89 | 113.94 ± 14.85 |
| Detroit (MI) | 2 | 4,125,648 | 380.2 ± 130.8 | 771.38 ± 164.87 | 905.44 ± 148.68 | 1325.3 ± 115.24 |
| Beaver Dam (WI) | 2 | 89,463 | 2.95 ± 2.47 | 10.26 ± 2.89 | 12.92 ± 2.93 | 21.08 ± 2.48 |
| Dover (DE) | 1 | 152,592 | 15.25 ± 4.47 | 31.33 ± 6.66 | 35.25 ± 5.79 | 51.25 ± 5.53 |
| Durham (NC) | 4 | 262,292 | -0.86 ± 2.92 | 10.37 ± 4.89 | 13 ± 4.66 | 25.64 ± 5.19 |
| Elizabeth (NJ) | 1 | 561,109 | 49.3 ± 12.56 | 97.74 ± 20 | 109.59 ± 16.73 | 153.3 ± 16.1 |
| Elkhart (IN) | 2 | 206,000 | 21.53 ± 9.49 | 39.52 ± 7.27 | 45.07 ± 6.28 | 61.77 ± 4.94 |
| El Paso (TX) | 8 | 820,495 | -7.51 ± 12.08 | 20.53 ± 14.4 | 26.95 ± 14.57 | 53.51 ± 15.32 |
| Erie (PA) | 2 | 287,899 | 9.98 ± 9.59 | 38.25 ± 11.58 | 48.3 ± 11.05 | 79.58 ± 7.59 |
| Eugene (OR) | 5 | 337,986 | -6.78 ± 6.45 | 6.63 ± 10.04 | 15.1 ± 9.55 | 41.8 ± 10.66 |
| Evansville (IN) | 3 | 174,247 | 9.7 ± 3.76 | 22.24 ± 5.44 | 25.75 ± 4.64 | 37.49 ± 4.06 |
| Everett (WA) | 5 | 705,799 | -19.41 ± 22.7 | 3.97 ± 14.86 | 16.87 ± 14.44 | 59.01 ± 12.55 |
| Fargo (ND) | 2 | 139,598 | 2.44 ± 3.15 | 9.19 ± 3.92 | 13.63 ± 3.91 | 21.7 ± 6.05 |
| Fayetteville (NC) | 4 | 317,492 | 8.16 ± 5.64 | 27.15 ± 7.71 | 31.33 ± 7.17 | 50.05 ± 7.5 |
| Flint (MI) | 2 | 451,035 | 14.11 ± 12.17 | 54.18 ± 17.09 | 67.94 ± 15.95 | 113.98 ± 12.92 |
| Fresno (CA) | 8 | 970,014 | -30.96 ± 16.85 | 4.12 ± 18.25 | 20.36 ± 16.43 | 67.31 ± 16.07 |
| Fort Lauderdale (FL) | 7 | 1,833,639 | 35.47 ± 24.31 | 93.01 ± 16.91 | 111.75 ± 14.89 | 168.55 ± 19.97 |
| Fort Myers (FL) | 7 | 616,755 | -1.78 ± 8.59 | 23.14 ± 6.2 | 31.11 ± 6.23 | 56.72 ± 8.4 |
| Fort Pierce (FL) | 7 | 410,505 | -4.16 ± 6.74 | 13.11 ± 5 | 18.58 ± 4.39 | 36.95 ± 6.24 |
| Fort Wayne (IN) | 2 | 356,260 | 23.14 ± 10.16 | 53.93 ± 13.23 | 63.02 ± 11.62 | 92.13 ± 9.66 |
| Fort Worth (TX) | 4 | 1,868,738 | 151.69 ± 44.13 | 263.97 ± 58.33 | 281.7 ± 61.5 | 387.42 ± 50.43 |
| Gary (IN) | 2 | 509,249 | 50.92 ± 15.2 | 93.22 ± 17.11 | 105.42 ± 15.55 | 143.6 ± 11.96 |
| Gainesville (FL) | 6 | 246,289 | -1.52 ± 1.85 | 6.5 ± 3.52 | 9.96 ± 3.71 | 24.99 ± 4.22 |
| Gettysburg (PA) | 1 | 103,888 | 10.05 ± 5.35 | 21.14 ± 4.49 | 24 ± 3.99 | 34.98 ± 3.5 |
| Grand Haven (MI) | 2 | 278,837 | 3.1 ± 4.95 | 19.21 ± 6.44 | 25.43 ± 5.74 | 43.76 ± 4.37 |
| Grand Junction (CO) | 9 | 134,793 | 8.34 ± 2.37 | 18.88 ± 3.59 | 22.42 ± 3.68 | 31.3 ± 3.76 |
| Grand Rapids (MI) | 2 | 655,332 | 20.27 ± 13.55 | 66.93 ± 19.35 | 83.28 ± 17.53 | 134.06 ± 13.83 |
| Green Bay (WI) | 2 | 270,814 | 1.33 ± 5.5 | 18.28 ± 6.95 | 25.69 ± 7.5 | 47.33 ± 7.34 |
| Greensboro (NC) | 4 | 468,766 | -7.7 ± 6.23 | 17.48 ± 10.77 | 24.01 ± 11.21 | 51.83 ± 13.23 |
| Greensburg (PA) | 3 | 353,508 | 6.41 ± 11.77 | 41.07 ± 15.33 | 51.52 ± 14.25 | 89.63 ± 12.09 |
| Greenville (SC) | 4 | 439,607 | 10.68 ± 17.9 | 37.53 ± 12.7 | 45.33 ± 12.18 | 75.85 ± 14.75 |
| Harrisburg (PA) | 1 | 268,917 | 24.62 ± 11.3 | 56.39 ± 13.68 | 64.16 ± 11.83 | 96.11 ± 9.84 |
| Hartford (CT) | 1 | 892,660 | 41.21 ± 25.18 | 113.76 ± 34.16 | 133.47 ± 29.78 | 206.76 ± 28.63 |
| Hickory (NC) | 4 | 160,102 | -2.85 ± 2.34 | 5.89 ± 4.02 | 8.33 ± 4.19 | 18.8 ± 4.95 |
| Holland (MI) | 2 | 117,602 | 2.09 ± 3.01 | 11.2 ± 3.68 | 14.73 ± 3.26 | 25.14 ± 2.51 |
| Houston (TX) | 6 | 3,968,385 | 71.75 ± 38.18 | 228.61 ± 63.85 | 268.85 ± 63.59 | 471.89 ± 47.19 |
| Indianapolis (IN) | 3 | 928,593 | 8.41 ± 15.6 | 61.82 ± 25.36 | 76.99 ± 22.17 | 127.51 ± 19.16 |
| Iowa City (IA) | 2 | 132,779 | 7.14 ± 3.74 | 16.95 ± 3.8 | 18.72 ± 3.11 | 26.66 ± 3.25 |
| Jacksonville (FL) | 6 | 888,355 | 13.14 ± 20.2 | 54.79 ± 19.61 | 68.53 ± 19.1 | 121.98 ± 18.04 |
| Jersey City (NJ) | 1 | 714,944 | -9.5 ± 12.42 | 24.11 ± 15.96 | 33.6 ± 14.68 | 71.13 ± 16.64 |
| Kalamazoo (MI) | 2 | 256,426 | 21.45 ± 10.78 | 45.12 ± 9.55 | 53.3 ± 8.5 | 77.34 ± 6.4 |
| Kansas City (KS) | 3 | 1,607,438 | 126.74 ± 41.55 | 236.43 ± 47.93 | 253.95 ± 38.94 | 349.19 ± 52.68 |
| Kenosha (WI) | 2 | 171,483 | 5.69 ± 5.48 | 17.62 ± 5.01 | 21.41 ± 4.83 | 33.14 ± 3.79 |
| Klamath Falls (OR) | 9 | 63,287 | -2.98 ± 1.08 | -1.43 ± 1.39 | -0.52 ± 1.18 | 2.76 ± 1.26 |
| Knoxville (TN) | 4 | 549,539 | -6.35 ± 8.89 | 25.17 ± 13.32 | 36.15 ± 15.33 | 78.21 ± 17.24 |
| Lafayette (IN) | 3 | 174,427 | 0.64 ± 2.96 | 10.51 ± 4.34 | 13.41 ± 3.93 | 23.21 ± 3.3 |
| Lafayette (LA) | 6 | 207,716 | 1.93 ± 2.73 | 10.87 ± 4.33 | 13.67 ± 3.99 | 27.67 ± 3.32 |
| Lakeland (FL) | 7 | 572,942 | 6.08 ± 10.9 | 29.13 ± 6.49 | 36.51 ± 5.7 | 59.96 ± 7.46 |
| Lancaster (PA) | 1 | 519,229 | 44 ± 17.44 | 98.16 ± 22.55 | 110.94 ± 19.4 | 163.76 ± 16.23 |
| Lansing (MI) | 2 | 314,338 | 8.08 ± 6.79 | 29.79 ± 9.45 | 37.62 ± 8.77 | 62.93 ± 7.18 |
| La Porte (IN) | 2 | 108,383 | 8.37 ± 3.99 | 19.48 ± 4.76 | 22.98 ± 4.25 | 33.84 ± 3.37 |
| Las Vegas (NV) | 8 | 2,074,364 | 69.48 ± 39.92 | 160.47 ± 35.01 | 189.77 ± 31.81 | 263.27 ± 24.23 |
| Lake Charles (LA) | 6 | 189,618 | 7.49 ± 8.47 | 20.14 ± 5.87 | 22.55 ± 5.46 | 38.72 ± 3.81 |
| Logan (UT) | 2 | 111,182 | 2.65 ± 3.19 | 9.14 ± 2.32 | 11.31 ± 2.21 | 16.79 ± 2.59 |
| Los Angeles (CA) | 5 | 10,559,243 | 915.04 ± 530.45 | 1475.92 ± 323.33 | 1718.42 ± 266.61 | 2338.59 ± 221.37 |
| Louisville (KY) | 3 | 738,978 | 41.81 ± 18.06 | 94.42 ± 24.12 | 109.76 ± 21.15 | 157.05 ± 17.01 |
| Little Rock (AR) | 4 | 380,611 | 18.07 ± 10 | 45.44 ± 15.08 | 52.2 ± 13.11 | 75.84 ± 10.89 |
| Macon (GA) | 4 | 157,307 | 5.93 ± 3.14 | 14.86 ± 4.13 | 17.82 ± 3.88 | 27.75 ± 3.45 |
| Madison (IL) | 3 | 273,407 | 26.79 ± 12.8 | 51.01 ± 9.44 | 55.81 ± 8.01 | 76.08 ± 8.02 |
| Madison (WI) | 2 | 571,173 | 15.73 ± 12.12 | 52.55 ± 14.53 | 65.11 ± 14.37 | 104.71 ± 12.9 |
| Upper Marlboro (MD) | 1 | 895,794 | 69.88 ± 21.38 | 136.1 ± 25.11 | 152.14 ± 23.42 | 219.21 ± 21.84 |
| Mcallen (TX) | 7 | 762,535 | 14.68 ± 6.34 | 38.09 ± 7.29 | 42.73 ± 6.72 | 64.69 ± 5.73 |
| Medford (OR) | 9 | 197,576 | 4.37 ± 4.79 | 12.47 ± 6.44 | 16.9 ± 5.57 | 31.55 ± 4.93 |
| Melbourne (FL) | 7 | 547,450 | -3.66 ± 9.27 | 20.68 ± 7.98 | 28.19 ± 6.5 | 53.08 ± 8.82 |
| Memphis (TN) | 4 | 954,592 | 55.77 ± 24.97 | 123.24 ± 32.83 | 144.76 ± 27.91 | 204 ± 24.68 |
| Mercer (PA) | 2 | 112,377 | 5.64 ± 5.73 | 17.84 ± 6.31 | 22.15 ± 5.65 | 37.78 ± 4.46 |
| Boise City (ID) | 9 | 386,522 | 7.24 ± 5.48 | 18.88 ± 8.3 | 23.93 ± 7.01 | 38.03 ± 6.51 |
| Miami (FL) | 7 | 3,099,526 | 56.56 ± 32.1 | 169.43 ± 36.48 | 210.34 ± 32.71 | 320.94 ± 47.33 |
| Middlesex (NJ) | 1 | 881,321 | 66.7 ± 26.04 | 135.26 ± 28.78 | 152.3 ± 24.36 | 215.9 ± 23.17 |
| Middletown (OH) | 3 | 383,546 | 5.2 ± 6.77 | 28.51 ± 10.64 | 35.37 ± 9.68 | 58.3 ± 8.14 |
| Milwaukee (WI) | 2 | 1,357,450 | 53.8 ± 56.35 | 150.27 ± 45.33 | 184.33 ± 44.07 | 292.19 ± 34.73 |
| Minneapolis (MN) | 2 | 1,743,595 | 71.22 ± 38.95 | 173.67 ± 41.94 | 211.14 ± 28.49 | 311.59 ± 49.79 |
| Mobile (AL) | 6 | 417,866 | 7.83 ± 10.48 | 25.43 ± 9.08 | 32.6 ± 9.11 | 61.42 ± 7.53 |
| Modesto (CA) | 8 | 573,131 | -20.62 ± 15.96 | -5.46 ± 11.1 | 2.1 ± 8.75 | 20.07 ± 9.22 |
| Toms River (NJ) | 1 | 587,588 | 70.58 ± 23.9 | 140.94 ± 28.68 | 157.98 ± 24.18 | 220.9 ± 22.9 |
| Monroe (LA) | 4 | 153,883 | 10.14 ± 3.73 | 21.58 ± 6.07 | 24.16 ± 5.43 | 35.46 ± 3.72 |
| Montgomery (AL) | 6 | 228,548 | 7.16 ± 6.96 | 19.06 ± 6.28 | 23.18 ± 6.98 | 38.83 ± 5.21 |
| Muncie (IN) | 3 | 114,470 | -2.48 ± 3.83 | 5.96 ± 3.74 | 8.42 ± 3.4 | 17.13 ± 3.03 |
| Muskegon (MI) | 2 | 177,808 | 3.07 ± 4.92 | 18.98 ± 6.37 | 25.14 ± 5.69 | 43.35 ± 4.35 |
| Myrtle Beach (SC) | 4 | 254,638 | 10.59 ± 5.43 | 26.32 ± 6.94 | 31.19 ± 6.33 | 48.59 ± 6.31 |
| Nampa (ID) | 9 | 185,267 | 3.63 ± 2.88 | 10.14 ± 4.69 | 13.25 ± 4.22 | 22.32 ± 3.85 |
| Nashua (NH) | 2 | 437,603 | 35.74 ± 13.51 | 69.03 ± 17.61 | 80.05 ± 14.73 | 114.29 ± 12.08 |
| Nashville (TN) | 4 | 652,834 | 3.76 ± 10.79 | 36.19 ± 17.87 | 47.38 ± 16.05 | 81.3 ± 13.74 |
| Melville (NY) | 1 | 3,000,255 | 97.04 ± 100.82 | 331.78 ± 101.86 | 392.5 ± 88.08 | 614.59 ± 94.32 |
| Newark (NJ) | 1 | 1,365,331 | 121.21 ± 31.34 | 241.5 ± 49.53 | 271.12 ± 41.55 | 379.5 ± 39.37 |
| Newburgh (NY) | 1 | 419,658 | 8.91 ± 8.23 | 39.2 ± 13.73 | 48.2 ± 12.23 | 80.63 ± 11.62 |
| New Haven (CT) | 1 | 872,769 | 40.48 ± 23.03 | 120.71 ± 35.16 | 141.22 ± 30.07 | 215.98 ± 28.8 |
| New London (CT) | 1 | 269,894 | 1.94 ± 8.37 | 22.46 ± 8.9 | 27.79 ± 7.81 | 47.59 ± 7.8 |
| Niles (MI) | 2 | 162,189 | 12.18 ± 6.3 | 29.2 ± 7.47 | 34.7 ± 6.6 | 51.84 ± 5.24 |
| Norfolk (VA) | 4 | 1,514,213 | 6.58 ± 18.14 | 76.45 ± 26.83 | 94.63 ± 27.71 | 171.02 ± 28.6 |
| New Orleans (LA) | 6 | 945,703 | 38.78 ± 32.91 | 98.31 ± 24.84 | 116.46 ± 22.61 | 192.86 ± 17.67 |
| New York (NY) | 1 | 10,396,046 | -198.35 ± 206.98 | 381.82 ± 274.59 | 544.6 ± 252.31 | 1183.25 ± 282.09 |
| Oakland (CA) | 5 | 2,651,188 | -77.33 ± 39.17 | -1.16 ± 53.71 | 27.12 ± 41.73 | 110.34 ± 54.98 |
| Ocala (FL) | 6 | 327,455 | -2.76 ± 6.16 | 13.89 ± 7.22 | 21.03 ± 7.59 | 51.4 ± 8.52 |
| Oklahoma City (OK) | 4 | 732,376 | 30.34 ± 21.5 | 85 ± 31.68 | 95.35 ± 30.35 | 139.46 ± 30.01 |
| Omaha (NE) | 2 | 525,046 | 52.22 ± 14.03 | 92.79 ± 15.96 | 100.53 ± 13.01 | 137.11 ± 17.48 |
| Orlando (FL) | 7 | 1,617,589 | -19.85 ± 15.39 | 29.79 ± 15.35 | 44.2 ± 13.79 | 96.13 ± 15.42 |
| Ottawa (IL) | 2 | 110,661 | 8.33 ± 3.85 | 17.44 ± 3.51 | 19.92 ± 3.3 | 28.38 ± 2.58 |
| Port Arthur (TX) | 6 | 262,282 | 4.24 ± 4.44 | 20.53 ± 6.66 | 24.92 ± 6.35 | 46.43 ± 4.8 |
| Palm Beach (FL) | 7 | 1,363,061 | -1.22 ± 25.16 | 62.09 ± 17.42 | 82.44 ± 15.73 | 146.22 ± 22.36 |
| Pensacola (FL) | 6 | 311,251 | 4.05 ± 5.05 | 16.13 ± 6.46 | 21.66 ± 7.1 | 43.69 ± 6.23 |
| Philadelphia (PA) | 1 | 4,875,946 | 445.6 ± 154.37 | 898.32 ± 195.98 | 1013.95 ± 170.39 | 1484.5 ± 163.84 |
| Phoenix (AZ) | 8 | 3,828,993 | 430.3 ± 64.69 | 642.09 ± 86.22 | 708.47 ± 71.49 | 863.76 ± 44.61 |
| Pittsburgh (PA) | 3 | 1,237,627 | -20.31 ± 32.85 | 88.8 ± 51.21 | 123.95 ± 47.95 | 258.6 ± 43.02 |
| Plymouth (MA) | 1 | 508,851 | 3.65 ± 14.14 | 34.65 ± 17.5 | 43.99 ± 15.53 | 80.67 ± 15.74 |
| Portage (IN) | 2 | 169,889 | 17.47 ± 5.24 | 31.95 ± 5.86 | 36.08 ± 5.28 | 49.26 ± 4.09 |
| Portland (OR) | 5 | 1,627,866 | 19.84 ± 31.45 | 98.31 ± 56.3 | 137.99 ± 52.05 | 253.6 ± 48.9 |
| Portland (ME) | 2 | 283,360 | -4.78 ± 4.3 | 14.65 ± 10.62 | 22.23 ± 9.83 | 46.78 ± 10.12 |
| Providence (RI) | 1 | 837,540 | 15.64 ± 17.21 | 77.17 ± 32.43 | 94.4 ± 28.51 | 161.63 ± 28.22 |
| Provo (UT) | 9 | 509,040 | 5.33 ± 4.94 | 24.11 ± 7.02 | 31.22 ± 6.89 | 47.79 ± 7.34 |
| Raleigh (NC) | 4 | 874,071 | -0.5 ± 7.75 | 29.98 ± 13.32 | 37.23 ± 12.82 | 71.77 ± 14.4 |
| Reading (PA) | 1 | 424,666 | 36.47 ± 14.68 | 83.75 ± 20.49 | 95.43 ± 17.79 | 143.57 ± 14.87 |
| Reno (NV) | 9 | 470,501 | 6.91 ± 11.14 | 27.09 ± 12.38 | 36.52 ± 9.84 | 63.39 ± 10.12 |
| Richmond (VA) | 1 | 863,737 | 112.23 ± 23.4 | 195.49 ± 28.59 | 215.33 ± 27.91 | 291.02 ± 28.35 |
| Riverside (CA) | 5 | 4,249,734 | 637.28 ± 130.5 | 877.96 ± 128.36 | 977.38 ± 93.68 | 1219.55 ± 81.4 |
| Rochester (NY) | 2 | 785,278 | 21.4 ± 18.45 | 88.77 ± 31.72 | 116.55 ± 30.56 | 197.89 ± 22.31 |
| Rockville (MD) | 1 | 993,900 | 103.06 ± 33.65 | 173.03 ± 24.94 | 189.72 ± 22.59 | 252.57 ± 21.23 |
| Sacramento (CA) | 5 | 1,443,068 | 195.6 ± 44.42 | 293.52 ± 71.2 | 330.73 ± 54.24 | 431.92 ± 40.35 |
| Essex (MA) | 1 | 756,210 | 17.09 ± 24.75 | 69.01 ± 27.57 | 87.12 ± 24.88 | 148.14 ± 23.38 |
| Salt Lake City (UT) | 9 | 1,063,702 | 33.18 ± 15.33 | 83.26 ± 16.13 | 100.74 ± 17.72 | 142.25 ± 17.2 |
| San Antonio (TX) | 6 | 1,685,188 | 70.04 ± 47.13 | 162.69 ± 44.27 | 181.72 ± 42.85 | 308.06 ± 38.8 |
| Sarasota (FL) | 7 | 705,040 | 5.05 ± 16.47 | 40.65 ± 10.5 | 52.07 ± 8.76 | 87.09 ± 11 |
| Santa Barbara (CA) | 5 | 425,999 | -0.29 ± 10.96 | 16.49 ± 8.76 | 23.77 ± 7.73 | 48.56 ± 8.55 |
| South Bend (IN) | 2 | 282,297 | 17.81 ± 9.07 | 42.62 ± 10.86 | 50.68 ± 9.7 | 75.43 ± 7.69 |
| St. Charles (MO) | 3 | 377,645 | 22.94 ± 7.55 | 46.21 ± 9.4 | 51.05 ± 7.97 | 71.36 ± 8 |
| East St. Louis (IL) | 3 | 266,433 | 22.81 ± 12.27 | 44.82 ± 8.71 | 49.31 ± 7.43 | 68.01 ± 7.41 |
| State College (PA) | 2 | 165,012 | 5.29 ± 6.3 | 20.16 ± 6.95 | 24.06 ± 6.36 | 40.78 ± 5.18 |
| Scranton (PA) | 2 | 503,640 | 24.81 ± 17.28 | 85.45 ± 27.35 | 102.56 ± 23.58 | 165.59 ± 20.16 |
| San Diego (CA) | 5 | 3,076,373 | 253.53 ± 136.63 | 429.77 ± 85.12 | 499.2 ± 54.26 | 658.94 ± 45.13 |
| Seattle (WA) | 5 | 2,095,098 | -104.25 ± 57.3 | -51.33 ± 36.43 | -20.27 ± 35.42 | 83.54 ± 32.44 |
| San Francisco (CA) | 5 | 1,474,044 | -26.22 ± 24.4 | 14.23 ± 30.97 | 29.57 ± 24.07 | 72.72 ± 29.78 |
| Sioux City (IA) | 2 | 104,608 | 10.47 ± 3.54 | 20.57 ± 3.91 | 22.87 ± 3.05 | 32.23 ± 4.69 |
| San Jose (CA) | 5 | 1,773,974 | 163.32 ± 64.33 | 244.87 ± 57.94 | 278.89 ± 45.08 | 355.76 ± 40.85 |
| Spartanburg (SC) | 4 | 290,717 | 1.47 ± 8.4 | 19.53 ± 9.13 | 25.15 ± 9.05 | 48.36 ± 11.47 |
| St. Petersburg (FL) | 7 | 947,997 | 40.12 ± 26.71 | 95.54 ± 16.27 | 113.84 ± 14.35 | 171.86 ± 18.32 |
| Spokane (WA) | 9 | 455,457 | -8.36 ± 8.65 | 8.74 ± 12.04 | 17.75 ± 10.47 | 42.99 ± 13.01 |
| Springfield (MA) | 1 | 470,398 | 20.8 ± 16.8 | 66.18 ± 20.79 | 80.09 ± 18.53 | 125.13 ± 18.2 |
| Springfield (MO) | 3 | 262,664 | 11.35 ± 6.33 | 31.97 ± 10.16 | 35.44 ± 7.91 | 51.18 ± 9.07 |
| Stamford (CT) | 1 | 929,629 | 15.78 ± 16.68 | 83.26 ± 29.47 | 102.75 ± 25.84 | 171.15 ± 25.42 |
| Steubenville (OH) | 3 | 108,793 | -0.08 ± 4.07 | 9.66 ± 4.52 | 12.97 ± 4.09 | 24.39 ± 3.72 |
| St. Louis (MO) | 3 | 1,620,561 | 151.73 ± 70.34 | 287.64 ± 53.07 | 315.27 ± 46.54 | 427.22 ± 44.67 |
| Stockton (CA) | 5 | 726,034 | 121.47 ± 19.32 | 156.35 ± 28.43 | 172.89 ± 21.9 | 209.86 ± 19.86 |
| Tacoma (WA) | 5 | 801,618 | -19.48 ± 28.32 | 7.55 ± 19.07 | 23.85 ± 19.48 | 79.04 ± 17.48 |
| Tallahassee (FL) | 6 | 286,424 | -0.73 ± 2.83 | 8.13 ± 4.35 | 11.96 ± 4.94 | 26.91 ± 4.85 |
| Tampa (FL) | 7 | 1,322,129 | -2.09 ± 12.97 | 46.42 ± 14.51 | 62.94 ± 12.72 | 116.54 ± 16.54 |
| Terre Haute (IN) | 3 | 101,706 | 1.51 ± 1.81 | 9.67 ± 3.01 | 11.57 ± 2.84 | 18.98 ± 2.53 |
| Toledo (OH) | 2 | 464,712 | 37.54 ± 21.62 | 84.44 ± 19 | 98.5 ± 16.26 | 144.53 ± 13.74 |
| Topeka (KS) | 3 | 177,127 | 8.57 ± 4.09 | 20.27 ± 5.47 | 22.26 ± 4.49 | 33.16 ± 6.46 |
| Trenton (NJ) | 1 | 392,619 | 28.19 ± 10.68 | 60.31 ± 13.95 | 68.6 ± 11.89 | 99.64 ± 11.25 |
| Tucson (AZ) | 8 | 980,969 | 7.18 ± 17.16 | 58.1 ± 19.83 | 72.99 ± 18.08 | 113.04 ± 18.47 |
| Tulsa (OK) | 4 | 603,879 | 37.01 ± 19.3 | 85.91 ± 27.31 | 94.61 ± 22.63 | 133.19 ± 23.02 |
| Vancouver (WA) | 5 | 489,556 | 3.84 ± 8.73 | 27.61 ± 16.38 | 39.18 ± 15.03 | 73.56 ± 13.95 |
| Ventura (CA) | 5 | 905,979 | 10.4 ± 31.61 | 43.38 ± 16.88 | 57.77 ± 14.72 | 104.27 ± 15.42 |
| Visalia (CA) | 8 | 478,651 | -16.98 ± 9.68 | -3.12 ± 8.54 | 3.14 ± 6.99 | 22.06 ± 7.46 |
| Washington (PA) | 3 | 196,082 | -5 ± 7.57 | 10.57 ± 7.45 | 16 ± 6.82 | 35.42 ± 6.28 |
| Washington (DC) | 1 | 810,015 | 112.2 ± 22.81 | 194.74 ± 29.43 | 214.46 ± 26.85 | 288.58 ± 25.11 |
| Ogden (UT) | 9 | 224,311 | 12.77 ± 5.18 | 25.82 ± 4.52 | 30.07 ± 4.54 | 40.42 ± 5.01 |
| Wichita (KS) | 3 | 506,519 | 41.5 ± 13.73 | 79.09 ± 18.81 | 85.82 ± 16.24 | 118.78 ± 20.51 |
| Wilmington (DE) | 1 | 556,014 | 43.83 ± 11.13 | 91.31 ± 19.83 | 102.97 ± 17.18 | 150.35 ± 16.49 |
| Winston-Salem (NC) | 4 | 340,276 | 1.26 ± 6.87 | 23.18 ± 9.18 | 28.74 ± 9.31 | 51.11 ± 10.38 |
| Worcester (MA) | 2 | 804,868 | 0.03 ± 14.27 | 53.04 ± 28.74 | 71.79 ± 25.74 | 135.13 ± 23.53 |
| York (PA) | 1 | 419,797 | 27.11 ± 11.08 | 67.24 ± 17.28 | 77.1 ± 15.15 | 117.95 ± 12.69 |
| Youngstown (OH) | 2 | 453,322 | 15.18 ± 12.99 | 61.94 ± 22.4 | 78.31 ± 19.9 | 135.17 ± 15.64 |

| **Table S4. Projected excess cold deaths (mean ± SD) for 209 cities, MIROC5 model** | | | | | | |
| --- | --- | --- | --- | --- | --- | --- |
|  |  |  | **Cold deaths (October–March)** | | | |
| **City** | **Cluster** | **Population (2010)** | **1990** | **2030** | **2050** | **2100** |
| Akron (OH) | 2 | 547,578 | -16.3 ± 12.11 | -31.64 ± 14.96 | -38.27 ± 13.92 | -66.71 ± 11.57 |
| Albany (NY) | 2 | 303,379 | 0.42 ± 6.02 | -8.19 ± 6.26 | -12.01 ± 7.18 | -29.4 ± 4 |
| Albuquerque (NM) | 9 | 652,540 | -19.39 ± 6.9 | -33.38 ± 6.78 | -35.51 ± 6.46 | -49.89 ± 8.43 |
| Allentown (PA) | 1 | 634,504 | 22.72 ± 18.25 | -4.07 ± 21.95 | -15.44 ± 21.51 | -67.58 ± 15.29 |
| Anaheim (CA) | 5 | 3,135,981 | -221.89 ± 70.91 | -286.25 ± 28.76 | -300.3 ± 41.65 | -365.1 ± 49.34 |
| Annandale (VA) | 1 | 1,350,383 | -45.16 ± 26.13 | -69.78 ± 26.8 | -83.43 ± 22.5 | -134.49 ± 19.21 |
| Ann Arbor (MI) | 2 | 369,089 | -1.54 ± 5.46 | -11 ± 5.89 | -13.72 ± 6.05 | -27.42 ± 4.81 |
| Atlanta (GA) | 4 | 3,982,479 | 16.99 ± 62.22 | -54.22 ± 81.54 | -96.05 ± 65.18 | -224.83 ± 80.68 |
| Atlantic City (NJ) | 1 | 301,495 | -8.29 ± 11.48 | -19.98 ± 11.49 | -25.94 ± 11.64 | -53.32 ± 7.68 |
| Aztec (NM) | 9 | 134,161 | 2.98 ± 2.07 | -0.47 ± 1.49 | -1.08 ± 1.96 | -4.2 ± 1.77 |
| Augusta (GA) | 4 | 200,965 | -8.32 ± 6.3 | -13.51 ± 6.19 | -16.59 ± 4.68 | -26.41 ± 5.44 |
| Austin (TX) | 6 | 1,186,535 | 26.04 ± 21.81 | 3.53 ± 23.24 | -8.73 ± 18.65 | -42.93 ± 31.13 |
| Bakersfield (CA) | 8 | 833,820 | 10.76 ± 31.06 | -23.64 ± 18.92 | -33.14 ± 27.19 | -84.42 ± 28.19 |
| Baltimore (MD) | 1 | 1,460,369 | -73.57 ± 54.5 | -133.36 ± 57.7 | -163.19 ± 51.85 | -285.41 ± 40 |
| Bangor (ME) | 2 | 149,052 | 6.27 ± 3.4 | 2 ± 3.06 | 0.15 ± 3.31 | -8.83 ± 2.21 |
| Barnstable (MA) | 1 | 231,855 | 2.92 ± 9.7 | -7.26 ± 9.48 | -12.18 ± 10.99 | -37.08 ± 5.92 |
| Bath (NY) | 2 | 94,238 | -0.04 ± 2.04 | -2.68 ± 2.05 | -3.8 ± 2.33 | -9.27 ± 1.64 |
| Paterson (NJ) | 1 | 1,532,376 | -5.12 ± 45.28 | -59.73 ± 42.97 | -82.04 ± 44.47 | -189.89 ± 30.38 |
| Birmingham (AL) | 4 | 993,979 | -28.4 ± 28.41 | -57.53 ± 35.5 | -73.59 ± 28.54 | -131.89 ± 38.59 |
| Boston (MA) | 1 | 2,888,305 | 61.37 ± 68.98 | -35.87 ± 81.99 | -83.22 ± 94.72 | -314.83 ± 51.11 |
| Boulder (CO) | 2 | 328,237 | -11.03 ± 5.56 | -16.27 ± 4.07 | -18.33 ± 4.34 | -26.26 ± 5.78 |
| Baton Rouge (LA) | 6 | 452,533 | 17.51 ± 13.52 | 3 ± 14.04 | -3.89 ± 12.11 | -29.97 ± 19 |
| Brownsville (TX) | 7 | 495,290 | 16.38 ± 10.86 | 8.46 ± 14.06 | 2.46 ± 11.66 | -10.69 ± 21.35 |
| Buffalo (NY) | 2 | 940,553 | -12.37 ± 18.72 | -43.85 ± 23.09 | -54.8 ± 24.84 | -111.9 ± 17.8 |
| Burlington (VT) | 2 | 156,597 | 4.15 ± 2.88 | 0.6 ± 2.58 | -1.23 ± 3.19 | -8.96 ± 2.24 |
| El Centro (CA) | 8 | 176,113 | -16.84 ± 6 | -23.94 ± 2.86 | -25.53 ± 3.03 | -33.95 ± 4.09 |
| Canton (OH) | 2 | 375,352 | -9.77 ± 8.38 | -20.28 ± 10.35 | -24.75 ± 9.47 | -43.99 ± 7.99 |
| Carlisle (PA) | 1 | 235,540 | 1.39 ± 7.7 | -9.55 ± 8.76 | -14.11 ± 8.28 | -34.24 ± 6.34 |
| Cedar Rapids (IA) | 2 | 208,038 | 3.17 ± 4.48 | -4.1 ± 5.39 | -5.52 ± 5.28 | -14.6 ± 5.64 |
| Charlotte (NC) | 4 | 975,223 | 13.51 ± 19.9 | -8.5 ± 26.6 | -21 ± 20.46 | -61.65 ± 19.9 |
| Charleston (SC) | 4 | 345,379 | -26.68 ± 7.02 | -32.19 ± 8.94 | -36.49 ± 6.99 | -50.14 ± 8.82 |
| Charleston (WV) | 3 | 183,151 | -2.58 ± 5.24 | -8.03 ± 7.39 | -11.07 ± 5.81 | -22.12 ± 5.77 |
| Chattanooga (TN) | 4 | 332,777 | 12.78 ± 9.27 | 1.41 ± 11.84 | -3.89 ± 9.81 | -22.98 ± 11.32 |
| Chicago (IL) | 2 | 7,213,360 | -251.44 ± 142.81 | -460.71 ± 143.94 | -514.65 ± 152.71 | -798.49 ± 130.54 |
| Cincinnati (OH) | 3 | 855,143 | 6.26 ± 18.1 | -19.17 ± 24.05 | -28.12 ± 22.08 | -68.76 ± 19.17 |
| Cleveland (OH) | 3 | 1,951,224 | 59.86 ± 46.21 | -7.14 ± 56.93 | -30.69 ± 53.73 | -143.78 ± 40.52 |
| Colorado Springs (CO) | 2 | 626,053 | -8.75 ± 7.98 | -20.95 ± 11.41 | -25.1 ± 10.43 | -42.11 ± 16.9 |
| Columbia (SC) | 4 | 639,401 | -19.75 ± 16.75 | -33.29 ± 18.68 | -42.78 ± 14.63 | -72.34 ± 17.34 |
| Columbus (OH) | 3 | 1,229,096 | 30.25 ± 25.17 | -2.91 ± 29.85 | -15.04 ± 27.51 | -66.04 ± 22.45 |
| Corpus Christi (TX) | 7 | 344,734 | 40.32 ± 11.85 | 30.93 ± 14.06 | 24.64 ± 10.96 | 8.78 ± 19.71 |
| Dallas (TX) | 4 | 2,697,303 | -125.85 ± 42.65 | -174.58 ± 48.72 | -197.06 ± 40.15 | -281.92 ± 61.68 |
| Davenport (IA) | 2 | 314,174 | -1.71 ± 7.2 | -14.49 ± 9.17 | -17.04 ± 9.14 | -34 ± 9.33 |
| Layton (UT) | 9 | 297,737 | 4.15 ± 3.33 | 1.22 ± 3.27 | -0.36 ± 4.21 | -5.8 ± 2.22 |
| Dayton (OH) | 3 | 550,109 | 18.7 ± 13.33 | -0.11 ± 18.48 | -6.61 ± 16.46 | -36.93 ± 14.12 |
| Daytona Beach (FL) | 7 | 509,402 | 80.76 ± 30.01 | 63.71 ± 28.33 | 49.62 ± 23.53 | -0.36 ± 36.35 |
| Denver (CO) | 2 | 1,738,073 | -37.56 ± 21.98 | -66.75 ± 27.88 | -79.03 ± 26.71 | -121.97 ± 38.19 |
| Des Moines (IA) | 2 | 452,715 | -1 ± 8.65 | -14.55 ± 10.72 | -17.48 ± 10.59 | -34.56 ± 12.31 |
| Detroit (MI) | 2 | 4,125,648 | -122.57 ± 87.76 | -263.23 ± 90.84 | -308.33 ± 96.29 | -522.95 ± 75.65 |
| Beaver Dam (WI) | 2 | 89,463 | 2.99 ± 2.09 | -0.25 ± 2.16 | -1.01 ± 2.29 | -5.52 ± 1.84 |
| Dover (DE) | 1 | 152,592 | -7.88 ± 5.23 | -14.43 ± 6.05 | -17.61 ± 5.74 | -31.11 ± 4.15 |
| Durham (NC) | 4 | 262,292 | 7.23 ± 5.42 | 1.54 ± 7.62 | -2.08 ± 6 | -14 ± 5.39 |
| Elizabeth (NJ) | 1 | 561,109 | -11.49 ± 15.68 | -31.43 ± 15.89 | -39.76 ± 16.5 | -79.53 ± 11.01 |
| Elkhart (IN) | 2 | 206,000 | -6.28 ± 4.02 | -12.16 ± 3.96 | -13.96 ± 4.14 | -22.53 ± 3.62 |
| El Paso (TX) | 8 | 820,495 | 53.27 ± 22.46 | 28.82 ± 13.1 | 19.19 ± 11.3 | -6.63 ± 16.54 |
| Erie (PA) | 2 | 287,899 | -8.59 ± 7.45 | -18.31 ± 6.39 | -21.44 ± 7.02 | -37.43 ± 4.81 |
| Eugene (OR) | 5 | 337,986 | 64.72 ± 11.72 | 54.86 ± 15.01 | 49.07 ± 13.88 | 37.43 ± 8.5 |
| Evansville (IN) | 3 | 174,247 | -2.86 ± 4.15 | -9.07 ± 5.4 | -10.74 ± 4.97 | -20.08 ± 5.05 |
| Everett (WA) | 5 | 705,799 | 95.83 ± 22.99 | 73.98 ± 24.51 | 61.73 ± 22.82 | 42.34 ± 11.63 |
| Fargo (ND) | 2 | 139,598 | 12.37 ± 3.58 | 8.12 ± 3.01 | 7.74 ± 3.53 | 2.12 ± 3.24 |
| Fayetteville (NC) | 4 | 317,492 | -3.4 ± 7.38 | -11.13 ± 10.3 | -16.17 ± 8.3 | -32.62 ± 7.53 |
| Flint (MI) | 2 | 451,035 | 1.41 ± 9.89 | -13.69 ± 10.19 | -18.24 ± 10.3 | -41.55 ± 8.27 |
| Fresno (CA) | 8 | 970,014 | 57.78 ± 30.82 | 15.82 ± 19.41 | 5.82 ± 27.57 | -43.02 ± 21.5 |
| Fort Lauderdale (FL) | 7 | 1,833,639 | -190.19 ± 68.6 | -217.7 ± 63.61 | -254.14 ± 57.81 | -353.09 ± 101.22 |
| Fort Myers (FL) | 7 | 616,755 | -10.32 ± 29.24 | -24.48 ± 24.12 | -40.23 ± 21.39 | -81.32 ± 36.58 |
| Fort Pierce (FL) | 7 | 410,505 | 0.38 ± 21.25 | -7.5 ± 17.68 | -17.85 ± 15.57 | -47.05 ± 26.44 |
| Fort Wayne (IN) | 2 | 356,260 | -6.42 ± 6.81 | -16.79 ± 7.81 | -19.78 ± 7.71 | -35.02 ± 7.01 |
| Fort Worth (TX) | 4 | 1,868,738 | -87.57 ± 30.98 | -127.93 ± 38.15 | -143.88 ± 30.66 | -212.89 ± 47.77 |
| Gary (IN) | 2 | 509,249 | -20.3 ± 10.13 | -34.81 ± 10.43 | -38.97 ± 11.26 | -59.96 ± 9.85 |
| Gainesville (FL) | 6 | 246,289 | -4.5 ± 7.62 | -9.24 ± 7.23 | -13.03 ± 5.95 | -25.4 ± 9.26 |
| Gettysburg (PA) | 1 | 103,888 | -1 ± 4.23 | -5.48 ± 4.33 | -7.61 ± 3.69 | -16.06 ± 3.01 |
| Grand Haven (MI) | 2 | 278,837 | -0.41 ± 3.76 | -6.57 ± 3.77 | -8.37 ± 4.15 | -17.46 ± 3.23 |
| Grand Junction (CO) | 9 | 134,793 | 2.95 ± 2.9 | -0.58 ± 2.09 | -1.59 ± 3.07 | -5.74 ± 2.12 |
| Grand Rapids (MI) | 2 | 655,332 | 2.05 ± 10.38 | -15.95 ± 11.58 | -21.06 ± 11.82 | -46.56 ± 9.58 |
| Green Bay (WI) | 2 | 270,814 | 11.07 ± 5.53 | 2.49 ± 5.22 | 0.72 ± 5.59 | -10.9 ± 4.74 |
| Greensboro (NC) | 4 | 468,766 | 28.06 ± 12.81 | 14.8 ± 17.66 | 6.67 ± 13.72 | -19.68 ± 12.14 |
| Greensburg (PA) | 3 | 353,508 | 8.76 ± 13.53 | -5.07 ± 13.95 | -10.85 ± 11.78 | -35.46 ± 9.71 |
| Greenville (SC) | 4 | 439,607 | -2.3 ± 16.62 | -16.5 ± 14.14 | -23.53 ± 11.34 | -46.44 ± 12.99 |
| Harrisburg (PA) | 1 | 268,917 | 1.67 ± 9.14 | -11.37 ± 10.44 | -16.87 ± 9.92 | -40.79 ± 7.55 |
| Hartford (CT) | 1 | 892,660 | 33.33 ± 30.02 | -2.43 ± 27.96 | -17.27 ± 31.41 | -90.93 ± 19.27 |
| Hickory (NC) | 4 | 160,102 | 8.33 ± 4.33 | 3.45 ± 5.65 | 0.89 ± 4.41 | -7.87 ± 4.51 |
| Holland (MI) | 2 | 117,602 | -0.01 ± 2.14 | -3.43 ± 2.17 | -4.42 ± 2.35 | -9.57 ± 1.8 |
| Houston (TX) | 6 | 3,968,385 | -64.15 ± 94.14 | -138.02 ± 91.03 | -187.2 ± 69.12 | -314.03 ± 119.6 |
| Indianapolis (IN) | 3 | 928,593 | 25.41 ± 17.55 | -0.82 ± 23.05 | -7.77 ± 21.55 | -46.47 ± 19 |
| Iowa City (IA) | 2 | 132,779 | 1.49 ± 2.72 | -2.13 ± 2.7 | -2.8 ± 2.63 | -7.34 ± 2.74 |
| Jacksonville (FL) | 6 | 888,355 | -49.75 ± 45.57 | -70.26 ± 30.14 | -83.21 ± 26.37 | -140.05 ± 41.37 |
| Jersey City (NJ) | 1 | 714,944 | 11.36 ± 17.81 | -7.68 ± 16.92 | -16.95 ± 18.25 | -58.15 ± 12.11 |
| Kalamazoo (MI) | 2 | 256,426 | -3.46 ± 4.7 | -11.76 ± 5.42 | -14.28 ± 5.64 | -26.41 ± 4.54 |
| Kansas City (KS) | 3 | 1,607,438 | -7.86 ± 30.78 | -61.34 ± 44.79 | -72.14 ± 40.99 | -137.09 ± 51.52 |
| Kenosha (WI) | 2 | 171,483 | -0.87 ± 3.56 | -6.99 ± 3.21 | -8.32 ± 3.53 | -15.02 ± 2.93 |
| Klamath Falls (OR) | 9 | 63,287 | 3.17 ± 1.34 | 1.76 ± 1.54 | 1.34 ± 1.64 | 0.13 ± 0.84 |
| Knoxville (TN) | 4 | 549,539 | 32.87 ± 13.83 | 15.2 ± 20.53 | 6.11 ± 16.22 | -25.61 ± 18.58 |
| Lafayette (IN) | 3 | 174,427 | 6.89 ± 4.03 | 1.29 ± 4.7 | -0.16 ± 4.41 | -8.2 ± 3.87 |
| Lafayette (LA) | 6 | 207,716 | 4.7 ± 6.62 | -2.3 ± 7.05 | -5.73 ± 6 | -18.82 ± 9.7 |
| Lakeland (FL) | 7 | 572,942 | 16.28 ± 41.36 | -0.69 ± 25.39 | -16.31 ± 22.94 | -58.32 ± 37.94 |
| Lancaster (PA) | 1 | 519,229 | 0.05 ± 17.86 | -21.68 ± 17.53 | -30.86 ± 16.58 | -71.92 ± 12.82 |
| Lansing (MI) | 2 | 314,338 | 2.27 ± 5.24 | -6.47 ± 5.89 | -9.05 ± 5.89 | -22.06 ± 4.77 |
| La Porte (IN) | 2 | 108,383 | -2.44 ± 2.43 | -6.06 ± 2.7 | -7.17 ± 2.8 | -12.68 ± 2.45 |
| Las Vegas (NV) | 8 | 2,074,364 | 68.92 ± 115.33 | -58.89 ± 83.3 | -92.94 ± 100.08 | -257.65 ± 85.97 |
| Lake Charles (LA) | 6 | 189,618 | 0.53 ± 11.07 | -8.12 ± 7.51 | -12.08 ± 6.16 | -25.39 ± 10.3 |
| Logan (UT) | 2 | 111,182 | 1.24 ± 1.82 | -0.45 ± 1.34 | -1.15 ± 1.91 | -3.82 ± 1.18 |
| Los Angeles (CA) | 5 | 10,559,243 | -563.69 ± 140.76 | -755.56 ± 127.54 | -805.07 ± 142.1 | -1034.02 ± 194.41 |
| Louisville (KY) | 3 | 738,978 | -17.37 ± 16.87 | -38.63 ± 19.73 | -45.74 ± 18.36 | -80.64 ± 16.63 |
| Little Rock (AR) | 4 | 380,611 | 2.69 ± 8.77 | -9.99 ± 11.81 | -15.39 ± 10.13 | -33.43 ± 16.25 |
| Macon (GA) | 4 | 157,307 | -9.09 ± 3.3 | -12.89 ± 4.7 | -15.2 ± 3.57 | -22.57 ± 4.43 |
| Madison (IL) | 3 | 273,407 | -6.39 ± 9.52 | -18.4 ± 9.95 | -21.25 ± 9.24 | -37.2 ± 11.11 |
| Madison (WI) | 2 | 571,173 | 13.4 ± 10.68 | -3.65 ± 10.88 | -7.45 ± 11.69 | -30.26 ± 10.09 |
| Upper Marlboro (MD) | 1 | 895,794 | -37.93 ± 21.42 | -60.57 ± 25.74 | -73.46 ± 22.75 | -126.31 ± 17.66 |
| Mcallen (TX) | 7 | 762,535 | 22.4 ± 17.18 | 3.71 ± 20.33 | -6.12 ± 16.64 | -30.82 ± 26.95 |
| Medford (OR) | 9 | 197,576 | -6.52 ± 3.44 | -10.1 ± 4.66 | -10.94 ± 4.52 | -14.77 ± 3.15 |
| Melbourne (FL) | 7 | 547,450 | 11.25 ± 27.69 | 1.05 ± 26.43 | -13.1 ± 23.06 | -55.33 ± 39.62 |
| Memphis (TN) | 4 | 954,592 | -3.8 ± 19.4 | -34.28 ± 31.21 | -47.25 ± 27.33 | -97.74 ± 36.66 |
| Mercer (PA) | 2 | 112,377 | -1.88 ± 3.17 | -5.7 ± 3.34 | -7.2 ± 3.3 | -14.31 ± 2.42 |
| Boise City (ID) | 9 | 386,522 | 5.62 ± 7.27 | -1.34 ± 7.07 | -4.56 ± 8.11 | -10.63 ± 4.44 |
| Miami (FL) | 7 | 3,099,526 | -265.85 ± 87.81 | -301.05 ± 83.93 | -357.1 ± 80.21 | -497.73 ± 137.83 |
| Middlesex (NJ) | 1 | 881,321 | -7.94 ± 24.96 | -36.39 ± 23.17 | -48.47 ± 24.04 | -106.69 ± 16.46 |
| Middletown (OH) | 3 | 383,546 | 2.88 ± 7.98 | -7.12 ± 9.45 | -10.59 ± 8.56 | -26.76 ± 7.68 |
| Milwaukee (WI) | 2 | 1,357,450 | 3.58 ± 29.04 | -38.86 ± 29.37 | -50.66 ± 31.65 | -111.7 ± 26.43 |
| Minneapolis (MN) | 2 | 1,743,595 | 60.87 ± 30.86 | 14.48 ± 28.07 | 7.83 ± 35.11 | -50.89 ± 33.87 |
| Mobile (AL) | 6 | 417,866 | 10 ± 18.59 | -2.76 ± 15.32 | -9.57 ± 13.23 | -35.67 ± 21.35 |
| Modesto (CA) | 8 | 573,131 | 30.33 ± 23.95 | 5.46 ± 12.68 | -1.2 ± 16.8 | -28.32 ± 11.9 |
| Toms River (NJ) | 1 | 587,588 | -26.85 ± 26.87 | -56.64 ± 22.5 | -68.7 ± 23.69 | -125.85 ± 15.83 |
| Monroe (LA) | 4 | 153,883 | -8.85 ± 3.83 | -13.61 ± 4.94 | -16.24 ± 4.2 | -24.51 ± 6.57 |
| Montgomery (AL) | 6 | 228,548 | 15.39 ± 8.22 | 8.41 ± 9.22 | 3.62 ± 8.33 | -12.39 ± 11.53 |
| Muncie (IN) | 3 | 114,470 | 8.58 ± 4.8 | 3.68 ± 3.99 | 2.31 ± 3.73 | -4.46 ± 3 |
| Muskegon (MI) | 2 | 177,808 | -0.5 ± 3.61 | -6.38 ± 3.6 | -8.1 ± 3.98 | -16.81 ± 3.14 |
| Myrtle Beach (SC) | 4 | 254,638 | -18.05 ± 7.71 | -22.43 ± 9.85 | -26.74 ± 8.05 | -40.99 ± 8.61 |
| Nampa (ID) | 9 | 185,267 | 2.89 ± 12.3 | -16.24 ± 15.72 | -20.92 ± 16.13 | -34.04 ± 12.42 |
| Nashua (NH) | 2 | 437,603 | -11.74 ± 9.04 | -22.36 ± 7.65 | -26.79 ± 8.77 | -48.9 ± 4.46 |
| Nashville (TN) | 4 | 652,834 | 25.24 ± 14.02 | 8.27 ± 19.34 | 1.05 ± 16.82 | -28.99 ± 17.91 |
| Melville (NY) | 1 | 3,000,255 | 0.45 ± 102.36 | -105.56 ± 87.29 | -153.51 ± 94.07 | -371.55 ± 63.59 |
| Newark (NJ) | 1 | 1,365,331 | -30.2 ± 38.73 | -79.38 ± 39.04 | -100 ± 40.71 | -198.49 ± 27.33 |
| Newburgh (NY) | 1 | 419,658 | 20.12 ± 10.44 | 4.93 ± 11.97 | -1.33 ± 13.1 | -32.5 ± 8.87 |
| New Haven (CT) | 1 | 872,769 | -3.89 ± 30.65 | -38.16 ± 27.99 | -53.22 ± 31.56 | -127.48 ± 18.96 |
| New London (CT) | 1 | 269,894 | 1.14 ± 7.95 | -8.94 ± 7.88 | -13.13 ± 8.83 | -33.91 ± 5.29 |
| Niles (MI) | 2 | 162,189 | -4.2 ± 3.99 | -10.23 ± 4.38 | -12.06 ± 4.55 | -20.94 ± 3.97 |
| Norfolk (VA) | 4 | 1,514,213 | 25.96 ± 28.46 | 3.38 ± 37.92 | -14.27 ± 35.43 | -72.06 ± 30.04 |
| New Orleans (LA) | 6 | 945,703 | -24.95 ± 40.89 | -58.22 ± 35.94 | -73.51 ± 29.3 | -132.03 ± 48.5 |
| New York (NY) | 1 | 10,396,046 | 157.69 ± 264.61 | -133.68 ± 256.18 | -272.97 ± 274.71 | -903.42 ± 184.41 |
| Oakland (CA) | 5 | 2,651,188 | -37.49 ± 51.44 | -85.85 ± 39.85 | -103.4 ± 40.21 | -155.88 ± 30.12 |
| Ocala (FL) | 6 | 327,455 | -19.82 ± 19.61 | -30.15 ± 15.67 | -38.22 ± 13.67 | -66.77 ± 22.06 |
| Oklahoma City (OK) | 4 | 732,376 | 36.17 ± 18.38 | 11.66 ± 22.04 | 3.66 ± 19.3 | -31.94 ± 29.88 |
| Omaha (NE) | 2 | 525,046 | -2.45 ± 10.76 | -17.76 ± 12.88 | -21.45 ± 12.87 | -41.1 ± 15.16 |
| Orlando (FL) | 7 | 1,617,589 | 80.18 ± 54.91 | 48 ± 49.49 | 20.49 ± 42.06 | -67.12 ± 66.43 |
| Ottawa (IL) | 2 | 110,661 | -1.09 ± 1.96 | -4.81 ± 2.62 | -5.66 ± 2.68 | -10.66 ± 2.33 |
| Port Arthur (TX) | 6 | 262,282 | 2.16 ± 7.93 | -6.79 ± 9.99 | -11.77 ± 7.7 | -25.48 ± 13.32 |
| Palm Beach (FL) | 7 | 1,363,061 | -88.18 ± 72.8 | -106.54 ± 56.89 | -139.36 ± 52.4 | -225.2 ± 90.27 |
| Pensacola (FL) | 6 | 311,251 | 7.16 ± 11.91 | -0.08 ± 11.29 | -4.9 ± 9.49 | -23.32 ± 15 |
| Philadelphia (PA) | 1 | 4,875,946 | -91.66 ± 174.13 | -285.83 ± 184.96 | -379.44 ± 173.39 | -784.71 ± 125.38 |
| Phoenix (AZ) | 8 | 3,828,993 | -308.01 ± 77.33 | -426.28 ± 57.14 | -454.88 ± 71.29 | -606.14 ± 83.65 |
| Pittsburgh (PA) | 3 | 1,237,627 | 55.14 ± 40.36 | 8.75 ± 49.77 | -11.34 ± 41.13 | -96.85 ± 33.36 |
| Plymouth (MA) | 1 | 508,851 | 21.21 ± 12.83 | 4.92 ± 16.02 | -2.96 ± 18.41 | -42.22 ± 11.11 |
| Portage (IN) | 2 | 169,889 | -6.97 ± 3.51 | -11.99 ± 3.58 | -13.37 ± 3.82 | -20.65 ± 3.36 |
| Portland (OR) | 5 | 1,627,866 | 224.37 ± 43.03 | 183.06 ± 60.54 | 159.15 ± 54.35 | 117.89 ± 31.89 |
| Portland (ME) | 2 | 283,360 | 2.39 ± 5.32 | -4.57 ± 5.32 | -7.89 ± 6.02 | -23.79 ± 3.04 |
| Providence (RI) | 1 | 837,540 | 27.97 ± 26.26 | -0.34 ± 27.86 | -14.1 ± 32 | -82.26 ± 18.96 |
| Provo (UT) | 9 | 509,040 | 6.43 ± 6.02 | -1.92 ± 5.65 | -4.3 ± 8.12 | -14.17 ± 4.67 |
| Raleigh (NC) | 4 | 874,071 | 20.52 ± 14.99 | 5.04 ± 20.48 | -4.71 ± 16.31 | -37.43 ± 14.4 |
| Reading (PA) | 1 | 424,666 | 5.64 ± 16.79 | -14.03 ± 15.7 | -22.18 ± 14.8 | -58.78 ± 11.52 |
| Reno (NV) | 9 | 470,501 | 3.12 ± 8.15 | -5.64 ± 8.09 | -7.22 ± 8.61 | -15.96 ± 6.72 |
| Richmond (VA) | 1 | 863,737 | -79.64 ± 25.69 | -104.16 ± 32.68 | -119.73 ± 27.79 | -178.75 ± 23.4 |
| Riverside (CA) | 5 | 4,249,734 | -66.69 ± 78.56 | -137.97 ± 43.28 | -156.88 ± 55.47 | -226.14 ± 55.61 |
| Rochester (NY) | 2 | 785,278 | -6.21 ± 14.55 | -26.62 ± 14.76 | -34.78 ± 17.59 | -77.02 ± 12.71 |
| Rockville (MD) | 1 | 993,900 | -62.4 ± 29.27 | -87.06 ± 25.4 | -100.36 ± 21.62 | -149.31 ± 18.08 |
| Sacramento (CA) | 5 | 1,443,068 | 36.43 ± 33.82 | -6.41 ± 26.37 | -16.73 ± 27.36 | -54.27 ± 18.45 |
| Essex (MA) | 1 | 756,210 | 26.65 ± 22.83 | -1.04 ± 23.22 | -14.38 ± 26.79 | -79.62 ± 14.6 |
| Salt Lake City (UT) | 9 | 1,063,702 | 9.3 ± 10.97 | -10.47 ± 12.62 | -16.04 ± 17.82 | -39.81 ± 11.35 |
| San Antonio (TX) | 6 | 1,685,188 | -5.77 ± 56.65 | -49.85 ± 44.46 | -73.93 ± 36.13 | -142.85 ± 59.23 |
| Sarasota (FL) | 7 | 705,040 | 17.89 ± 48.6 | -4.92 ± 37.25 | -28.38 ± 33.64 | -93.48 ± 56.18 |
| Santa Barbara (CA) | 5 | 425,999 | -15.23 ± 7.26 | -24.42 ± 5.41 | -27.53 ± 7.35 | -39.26 ± 7.68 |
| South Bend (IN) | 2 | 282,297 | -6.3 ± 6.12 | -15.49 ± 6.68 | -18.22 ± 6.92 | -31.73 ± 6.03 |
| St. Charles (MO) | 3 | 377,645 | -3.63 ± 7.44 | -14.65 ± 9.21 | -17.11 ± 8.48 | -32.38 ± 10.23 |
| East St. Louis (IL) | 3 | 266,433 | -5.24 ± 8.33 | -15.51 ± 8.73 | -17.77 ± 7.87 | -31.87 ± 9.66 |
| State College (PA) | 2 | 165,012 | -3.34 ± 4.2 | -8.03 ± 3.62 | -9.92 ± 3.32 | -17.79 ± 2.6 |
| Scranton (PA) | 2 | 503,640 | -15.45 ± 13.63 | -33.98 ± 14.19 | -41.14 ± 15.12 | -76.59 ± 9.94 |
| San Diego (CA) | 5 | 3,076,373 | -218.67 ± 65.05 | -271.47 ± 26.84 | -288.05 ± 36.05 | -350.97 ± 40.39 |
| Seattle (WA) | 5 | 2,095,098 | 241.99 ± 46.2 | 201.22 ± 60.31 | 167.1 ± 58.06 | 121.07 ± 26.77 |
| San Francisco (CA) | 5 | 1,474,044 | -57.42 ± 28.53 | -89.81 ± 27.23 | -101.99 ± 28.37 | -143.56 ± 23.98 |
| Sioux City (IA) | 2 | 104,608 | 2.57 ± 3 | -1.6 ± 3.56 | -2.58 ± 3.6 | -7.97 ± 4.21 |
| San Jose (CA) | 5 | 1,773,974 | -65.99 ± 26.96 | -99.73 ± 21.56 | -107.9 ± 21.58 | -140.16 ± 18.18 |
| Spartanburg (SC) | 4 | 290,717 | 3.76 ± 8.48 | -4.55 ± 10.56 | -9.66 ± 8.25 | -26.44 ± 9.33 |
| St. Petersburg (FL) | 7 | 947,997 | -3.01 ± 65.4 | -36.08 ± 48.79 | -66.33 ± 42.81 | -153.9 ± 73.93 |
| Spokane (WA) | 9 | 455,457 | 18.35 ± 7.51 | 10.88 ± 12.53 | 5.01 ± 11.3 | -1.07 ± 5.86 |
| Springfield (MA) | 1 | 470,398 | 31.91 ± 15.31 | 11.18 ± 16.38 | 1.48 ± 18.72 | -44.52 ± 10.73 |
| Springfield (MO) | 3 | 262,664 | -4.01 ± 5.56 | -12.28 ± 7.83 | -14.38 ± 6.97 | -26.61 ± 9.91 |
| Stamford (CT) | 1 | 929,629 | 27.67 ± 27.43 | -7.36 ± 25.65 | -21.83 ± 28.43 | -93.03 ± 17.79 |
| Steubenville (OH) | 3 | 108,793 | 4.16 ± 4.53 | -0.25 ± 4.59 | -2.11 ± 3.92 | -9.86 ± 3.11 |
| St. Louis (MO) | 3 | 1,620,561 | -40.95 ± 45.31 | -102.65 ± 47.34 | -115.78 ± 45.16 | -196.64 ± 56.78 |
| Stockton (CA) | 5 | 726,034 | 15.51 ± 18.3 | -7.09 ± 12.42 | -12.47 ± 13.21 | -31.59 ± 9.33 |
| Tacoma (WA) | 5 | 801,618 | 125.5 ± 29.25 | 101.67 ± 30.32 | 87.68 ± 27.49 | 63.55 ± 13.66 |
| Tallahassee (FL) | 6 | 286,424 | 7.52 ± 8.45 | 1.78 ± 7.69 | -1.85 ± 6.57 | -15.52 ± 10.04 |
| Tampa (FL) | 7 | 1,322,129 | 70.86 ± 56.38 | 44.06 ± 44.77 | 15.81 ± 39.31 | -64.76 ± 66.22 |
| Terre Haute (IN) | 3 | 101,706 | 3.24 ± 3.3 | -1.4 ± 3.87 | -2.54 ± 3.67 | -8.91 ± 3.33 |
| Toledo (OH) | 2 | 464,712 | -10.63 ± 9.01 | -26.47 ± 10.7 | -31.29 ± 10.74 | -54.06 ± 8.98 |
| Topeka (KS) | 3 | 177,127 | 2.97 ± 4.23 | -3.25 ± 5.56 | -4.57 ± 5.03 | -12.24 ± 6.81 |
| Trenton (NJ) | 1 | 392,619 | -3.6 ± 11.63 | -16.53 ± 11.12 | -22.36 ± 11.62 | -50.08 ± 7.83 |
| Tucson (AZ) | 8 | 980,969 | -2.04 ± 23.67 | -32.74 ± 15.18 | -39.71 ± 20.12 | -74.09 ± 20.76 |
| Tulsa (OK) | 4 | 603,879 | 28.28 ± 16.72 | 2.72 ± 22.92 | -4.55 ± 20.02 | -40.44 ± 29.58 |
| Vancouver (WA) | 5 | 489,556 | 73.21 ± 13.87 | 60.01 ± 19.05 | 52.16 ± 17.28 | 38.26 ± 10.21 |
| Ventura (CA) | 5 | 905,979 | -44.57 ± 21.78 | -62.21 ± 9.04 | -67.65 ± 12.57 | -87.52 ± 13.23 |
| Visalia (CA) | 8 | 478,651 | 25 ± 14 | 6.65 ± 8.46 | 2.19 ± 11.83 | -20.09 ± 10.18 |
| Washington (PA) | 3 | 196,082 | 8.91 ± 7.85 | 1.3 ± 7.97 | -2.01 ± 6.9 | -15.51 ± 5.39 |
| Washington (DC) | 1 | 810,015 | -57.91 ± 20.73 | -82.29 ± 27.79 | -96.61 ± 23.43 | -149.91 ± 19.85 |
| Ogden (UT) | 9 | 224,311 | 1.84 ± 4.14 | -2.18 ± 3.5 | -4.05 ± 4.71 | -9.94 ± 2.87 |
| Wichita (KS) | 3 | 506,519 | -3.76 ± 10.21 | -19.11 ± 13.29 | -23.45 ± 11.87 | -45.49 ± 18.58 |
| Wilmington (DE) | 1 | 556,014 | -10.85 ± 15.91 | -29.24 ± 17.7 | -38.56 ± 16.89 | -77.79 ± 12.15 |
| Winston-Salem (NC) | 4 | 340,276 | 11.01 ± 9.61 | 0.74 ± 13.61 | -5.53 ± 10.61 | -25.94 ± 9.5 |
| Worcester (MA) | 2 | 804,868 | 0.73 ± 14.82 | -17.63 ± 14.94 | -26.23 ± 16.99 | -67.59 ± 8.45 |
| York (PA) | 1 | 419,797 | 4.26 ± 12.44 | -12.57 ± 13.63 | -19.65 ± 12.87 | -50.52 ± 9.71 |
| Youngstown (OH) | 2 | 453,322 | -9.6 ± 11.57 | -25.23 ± 12.7 | -31.03 ± 12.74 | -57.42 ± 9.33 |

| **Table S5.** **Projected excess heat deaths (mean ± SD) for 209 cities, MIROC5 model** | | | | | | |
| --- | --- | --- | --- | --- | --- | --- |
|  |  |  | **Heat deaths (April–September)** | | | |
| **City** | **Cluster** | **Population (2010)** | **1990** | **2030** | **2050** | **2100** |
| Akron (OH) | 2 | 547,578 | 28.72 ± 16.86 | 55.17 ± 15.51 | 63.12 ± 14.64 | 107.43 ± 18.29 |
| Albany (NY) | 2 | 303,379 | 9.07 ± 6.44 | 22.03 ± 7.01 | 26.5 ± 7.86 | 48.99 ± 6.94 |
| Albuquerque (NM) | 9 | 652,540 | 51.07 ± 12.38 | 71.12 ± 15.45 | 85.42 ± 13.7 | 115.21 ± 14.72 |
| Allentown (PA) | 1 | 634,504 | 29.17 ± 14.89 | 58.59 ± 16.01 | 67.1 ± 15.43 | 118.84 ± 19.47 |
| Anaheim (CA) | 5 | 3,135,981 | 363.22 ± 162.37 | 498.13 ± 82.38 | 560.13 ± 68.61 | 684.86 ± 88.77 |
| Annandale (VA) | 1 | 1,350,383 | 92.35 ± 28.83 | 131.02 ± 18.56 | 140.12 ± 16.63 | 200.64 ± 20.08 |
| Ann Arbor (MI) | 2 | 369,089 | 13.72 ± 7.82 | 29.85 ± 9.85 | 34.51 ± 8.92 | 56.11 ± 10.21 |
| Atlanta (GA) | 4 | 3,982,479 | 19.38 ± 51.83 | 95.56 ± 40.79 | 130.58 ± 45.29 | 252.83 ± 43.17 |
| Atlantic City (NJ) | 1 | 301,495 | 20.44 ± 7.88 | 33.23 ± 8.58 | 37.5 ± 8.23 | 64.69 ± 11.47 |
| Aztec (NM) | 9 | 134,161 | 3.25 ± 2.67 | 6.77 ± 2.23 | 9.07 ± 2.05 | 14.17 ± 3.44 |
| Augusta (GA) | 4 | 200,965 | 6.91 ± 4.58 | 13.56 ± 3.39 | 16.47 ± 3.63 | 27.23 ± 3.17 |
| Austin (TX) | 6 | 1,186,535 | 23.95 ± 15.74 | 53.45 ± 16.38 | 74.53 ± 23.51 | 109.22 ± 22.3 |
| Bakersfield (CA) | 8 | 833,820 | -13.27 ± 13.47 | 1.77 ± 13.5 | 10.88 ± 12.51 | 27.61 ± 17.43 |
| Baltimore (MD) | 1 | 1,460,369 | 180.7 ± 59.09 | 277.37 ± 43.58 | 300.16 ± 39.44 | 440.99 ± 47.73 |
| Bangor (ME) | 2 | 149,052 | -4 ± 2.41 | 0.64 ± 3.09 | 3.87 ± 4.58 | 14.53 ± 3.7 |
| Barnstable (MA) | 1 | 231,855 | -1.01 ± 7.29 | 5.92 ± 7.77 | 9.42 ± 8.02 | 29.33 ± 8.48 |
| Bath (NY) | 2 | 94,238 | 1.52 ± 2.5 | 5.66 ± 2.87 | 7.13 ± 2.83 | 14.38 ± 3.09 |
| Paterson (NJ) | 1 | 1,532,376 | 103.23 ± 43.3 | 168.46 ± 35.7 | 186.25 ± 34.12 | 294.62 ± 40.62 |
| Birmingham (AL) | 4 | 993,979 | 28.5 ± 24.75 | 62.01 ± 16.35 | 77.59 ± 19.68 | 136.88 ± 21.61 |
| Boston (MA) | 1 | 2,888,305 | 68 ± 52.8 | 147.22 ± 58.14 | 187.01 ± 66.8 | 366.08 ± 55.29 |
| Boulder (CO) | 2 | 328,237 | 19.34 ± 10.57 | 26.66 ± 7.3 | 33.3 ± 6.06 | 49.86 ± 9.28 |
| Baton Rouge (LA) | 6 | 452,533 | 2.07 ± 5.49 | 20.02 ± 8.26 | 23.1 ± 11.35 | 54.45 ± 10.76 |
| Brownsville (TX) | 7 | 495,290 | 5.8 ± 3.94 | 11.54 ± 4.4 | 14.98 ± 4.77 | 21.45 ± 6.39 |
| Buffalo (NY) | 2 | 940,553 | 19.16 ± 24.83 | 66.61 ± 30.47 | 82.87 ± 29.65 | 160.91 ± 32.97 |
| Burlington (VT) | 2 | 156,597 | 1.72 ± 2.53 | 6.66 ± 3.43 | 9.32 ± 3.87 | 19.6 ± 3.83 |
| El Centro (CA) | 8 | 176,113 | 13.32 ± 4.02 | 17.99 ± 2.62 | 19.97 ± 1.84 | 23.85 ± 2.64 |
| Canton (OH) | 2 | 375,352 | 18.61 ± 11.22 | 36.77 ± 10.61 | 42.22 ± 10.07 | 72.58 ± 12.57 |
| Carlisle (PA) | 1 | 235,540 | 19.64 ± 9.05 | 32.57 ± 6.9 | 36.08 ± 6.27 | 56.77 ± 7.33 |
| Cedar Rapids (IA) | 2 | 208,038 | 11.94 ± 5.62 | 24.76 ± 5.43 | 26.51 ± 4.44 | 41.77 ± 6.79 |
| Charlotte (NC) | 4 | 975,223 | 2.73 ± 13.63 | 24.73 ± 9.8 | 31.07 ± 9.95 | 67.15 ± 10.93 |
| Charleston (SC) | 4 | 345,379 | 14.95 ± 4.96 | 23.65 ± 4.81 | 27.82 ± 5.55 | 45.1 ± 4.59 |
| Charleston (WV) | 3 | 183,151 | 1.17 ± 4.33 | 10.07 ± 4.46 | 12.32 ± 4.07 | 25.06 ± 5.21 |
| Chattanooga (TN) | 4 | 332,777 | 1.6 ± 8.2 | 13.59 ± 5.55 | 17.49 ± 5.8 | 35.32 ± 5.9 |
| Chicago (IL) | 2 | 7,213,360 | 599.51 ± 213.88 | 966.11 ± 191.84 | 1047.18 ± 159.88 | 1531.36 ± 211.32 |
| Cincinnati (OH) | 3 | 855,143 | 13.14 ± 15.62 | 51.28 ± 17.59 | 58.58 ± 15.73 | 103.48 ± 20.77 |
| Cleveland (OH) | 3 | 1,951,224 | 5.1 ± 48.34 | 89.29 ± 47.57 | 118.22 ± 48.66 | 247.05 ± 58.99 |
| Colorado Springs (CO) | 2 | 626,053 | 3.62 ± 9.74 | 14.89 ± 13.49 | 27.12 ± 13.48 | 60.78 ± 18.68 |
| Columbia (SC) | 4 | 639,401 | 21.22 ± 12.26 | 39.69 ± 10.16 | 48.28 ± 10.96 | 80.3 ± 9.21 |
| Columbus (OH) | 3 | 1,229,096 | -0.95 ± 22.68 | 42.18 ± 22.92 | 53.86 ± 21.54 | 110.89 ± 27.79 |
| Corpus Christi (TX) | 7 | 344,734 | 2.7 ± 3.07 | 9.36 ± 3.28 | 13.59 ± 3.88 | 21.21 ± 4.27 |
| Dallas (TX) | 4 | 2,697,303 | 184.86 ± 50.8 | 280.47 ± 55.63 | 322.67 ± 60.43 | 428.26 ± 55.04 |
| Davenport (IA) | 2 | 314,174 | 30.23 ± 10.58 | 54.41 ± 10.11 | 57.53 ± 8.64 | 86.76 ± 11.9 |
| Layton (UT) | 9 | 297,737 | 5.12 ± 4.36 | 11.8 ± 3.45 | 15.19 ± 3.04 | 23.97 ± 5.6 |
| Dayton (OH) | 3 | 550,109 | 0.97 ± 13.19 | 25.37 ± 12.09 | 30.44 ± 10.9 | 62.16 ± 15.06 |
| Daytona Beach (FL) | 7 | 509,402 | -21.12 ± 8.61 | -5.25 ± 8.07 | -1.22 ± 7.25 | 32.19 ± 6.52 |
| Denver (CO) | 2 | 1,738,073 | 60.96 ± 32.08 | 102.62 ± 38.82 | 136.76 ± 35.04 | 225.18 ± 51.05 |
| Des Moines (IA) | 2 | 452,715 | 40.61 ± 11.97 | 67.58 ± 11.45 | 70.2 ± 8.91 | 100.96 ± 13.15 |
| Detroit (MI) | 2 | 4,125,648 | 380.2 ± 130.8 | 625.49 ± 154.62 | 699.19 ± 138.08 | 1027.81 ± 152.79 |
| Beaver Dam (WI) | 2 | 89,463 | 2.95 ± 2.47 | 8.42 ± 3.18 | 10.11 ± 3 | 17.95 ± 3.47 |
| Dover (DE) | 1 | 152,592 | 15.25 ± 4.47 | 24.26 ± 4.43 | 26.59 ± 4.14 | 40.98 ± 5.23 |
| Durham (NC) | 4 | 262,292 | -0.86 ± 2.92 | 5.01 ± 2.59 | 6.51 ± 2.62 | 17.76 ± 3.22 |
| Elizabeth (NJ) | 1 | 561,109 | 49.3 ± 12.56 | 75.53 ± 13.93 | 82.37 ± 13.22 | 123.1 ± 15.07 |
| Elkhart (IN) | 2 | 206,000 | 21.53 ± 9.49 | 33.46 ± 6.61 | 36.55 ± 5.57 | 50.84 ± 6.47 |
| El Paso (TX) | 8 | 820,495 | -7.51 ± 12.08 | 7.6 ± 8.03 | 15.97 ± 8.03 | 35.63 ± 7.39 |
| Erie (PA) | 2 | 287,899 | 9.98 ± 9.59 | 25.14 ± 9.25 | 30.1 ± 9.46 | 54.03 ± 10.99 |
| Eugene (OR) | 5 | 337,986 | -6.78 ± 6.45 | 0.95 ± 9.06 | 5.15 ± 8.52 | 20.71 ± 7.38 |
| Evansville (IN) | 3 | 174,247 | 9.7 ± 3.76 | 19.7 ± 4.09 | 20.54 ± 3.04 | 31.69 ± 4.52 |
| Everett (WA) | 5 | 705,799 | -19.41 ± 22.7 | -2.54 ± 15.3 | 3.51 ± 13.73 | 26.79 ± 12.4 |
| Fargo (ND) | 2 | 139,598 | 2.44 ± 3.15 | 8.47 ± 4.1 | 10.43 ± 4.41 | 19.97 ± 5.13 |
| Fayetteville (NC) | 4 | 317,492 | 8.16 ± 5.64 | 18.48 ± 4.54 | 21.2 ± 4.44 | 38.95 ± 4.98 |
| Flint (MI) | 2 | 451,035 | 14.11 ± 12.17 | 38.65 ± 15.79 | 45.99 ± 14.4 | 81.03 ± 16.86 |
| Fresno (CA) | 8 | 970,014 | -30.96 ± 16.85 | -3.02 ± 19.57 | 13.79 ± 19.25 | 48.5 ± 31.23 |
| Fort Lauderdale (FL) | 7 | 1,833,639 | 35.47 ± 24.31 | 64.31 ± 18.27 | 80.91 ± 17.04 | 125.14 ± 12.2 |
| Fort Myers (FL) | 7 | 616,755 | -1.78 ± 8.59 | 10.79 ± 5.32 | 17.67 ± 5.49 | 38.73 ± 3.77 |
| Fort Pierce (FL) | 7 | 410,505 | -4.16 ± 6.74 | 3.69 ± 4.67 | 8.89 ± 4.44 | 23.84 ± 3.13 |
| Fort Wayne (IN) | 2 | 356,260 | 23.14 ± 10.16 | 43.81 ± 10.89 | 47.58 ± 9.39 | 73.13 ± 11.68 |
| Fort Worth (TX) | 4 | 1,868,738 | 151.69 ± 44.13 | 223.83 ± 49.39 | 262.41 ± 50.09 | 344.76 ± 48.15 |
| Gary (IN) | 2 | 509,249 | 50.92 ± 15.2 | 79.66 ± 15.57 | 85.51 ± 13.23 | 123.83 ± 16.35 |
| Gainesville (FL) | 6 | 246,289 | -1.52 ± 1.85 | 2.01 ± 2.52 | 3.41 ± 2.85 | 18.59 ± 3.04 |
| Gettysburg (PA) | 1 | 103,888 | 10.05 ± 5.35 | 15.96 ± 2.84 | 17.44 ± 2.46 | 26.34 ± 3 |
| Grand Haven (MI) | 2 | 278,837 | 3.1 ± 4.95 | 12.15 ± 5.74 | 15.34 ± 5.44 | 31.09 ± 6.68 |
| Grand Junction (CO) | 9 | 134,793 | 8.34 ± 2.37 | 13.58 ± 2.95 | 16.02 ± 2.1 | 22.14 ± 4.04 |
| Grand Rapids (MI) | 2 | 655,332 | 20.27 ± 13.55 | 49.64 ± 18.09 | 59.02 ± 15.89 | 102.23 ± 18.19 |
| Green Bay (WI) | 2 | 270,814 | 1.33 ± 5.5 | 13.87 ± 7.81 | 17.83 ± 7.66 | 36.72 ± 9.15 |
| Greensboro (NC) | 4 | 468,766 | -7.7 ± 6.23 | 5.09 ± 5.8 | 8.35 ± 5.81 | 30.69 ± 7.62 |
| Greensburg (PA) | 3 | 353,508 | 6.41 ± 11.77 | 23.72 ± 9.23 | 28.85 ± 8.73 | 54.84 ± 10.53 |
| Greenville (SC) | 4 | 439,607 | 10.68 ± 17.9 | 24 ± 7.82 | 29.48 ± 7.8 | 51.13 ± 7.4 |
| Harrisburg (PA) | 1 | 268,917 | 24.62 ± 11.3 | 40.71 ± 8.61 | 45.11 ± 7.84 | 71 ± 9.07 |
| Hartford (CT) | 1 | 892,660 | 41.21 ± 25.18 | 75.45 ± 21.99 | 87.97 ± 23.3 | 157.73 ± 22.08 |
| Hickory (NC) | 4 | 160,102 | -2.85 ± 2.34 | 1.33 ± 2.03 | 2.62 ± 2.04 | 9.71 ± 2.43 |
| Holland (MI) | 2 | 117,602 | 2.09 ± 3.01 | 7.17 ± 3.29 | 9.03 ± 3.11 | 17.99 ± 3.8 |
| Houston (TX) | 6 | 3,968,385 | 71.75 ± 38.18 | 167.74 ± 43.9 | 230.3 ± 60.93 | 353.51 ± 60.67 |
| Indianapolis (IN) | 3 | 928,593 | 8.41 ± 15.6 | 47.33 ± 17.3 | 51.44 ± 14.8 | 99.03 ± 21.55 |
| Iowa City (IA) | 2 | 132,779 | 7.14 ± 3.74 | 13.88 ± 2.85 | 14.76 ± 2.34 | 22.75 ± 3.52 |
| Jacksonville (FL) | 6 | 888,355 | 13.14 ± 20.2 | 34.16 ± 15.54 | 39.66 ± 15.34 | 108.23 ± 15.14 |
| Jersey City (NJ) | 1 | 714,944 | -9.5 ± 12.42 | 4.7 ± 10.42 | 9.38 ± 10.2 | 39.57 ± 13.17 |
| Kalamazoo (MI) | 2 | 256,426 | 21.45 ± 10.78 | 36.77 ± 9.12 | 41.67 ± 7.84 | 62.66 ± 8.56 |
| Kansas City (KS) | 3 | 1,607,438 | 126.74 ± 41.55 | 221.92 ± 43.64 | 228.61 ± 32.42 | 324.86 ± 60.52 |
| Kenosha (WI) | 2 | 171,483 | 5.69 ± 5.48 | 13.58 ± 5.02 | 15.82 ± 4.54 | 27.3 ± 5.48 |
| Klamath Falls (OR) | 9 | 63,287 | -2.98 ± 1.08 | -1.84 ± 0.95 | -1.3 ± 1.17 | 0.2 ± 1.22 |
| Knoxville (TN) | 4 | 549,539 | -6.35 ± 8.89 | 8.21 ± 6.99 | 13.21 ± 7.29 | 38.77 ± 7.62 |
| Lafayette (IN) | 3 | 174,427 | 0.64 ± 2.96 | 7.32 ± 3.46 | 8.5 ± 3.14 | 17.48 ± 4.36 |
| Lafayette (LA) | 6 | 207,716 | 1.93 ± 2.73 | 10.9 ± 3.85 | 12.4 ± 5.3 | 27.3 ± 4.93 |
| Lakeland (FL) | 7 | 572,942 | 6.08 ± 10.9 | 18.53 ± 4.78 | 24.04 ± 5.12 | 44.98 ± 3.9 |
| Lancaster (PA) | 1 | 519,229 | 44 ± 17.44 | 72.75 ± 14.52 | 80.16 ± 13.25 | 123.05 ± 15.2 |
| Lansing (MI) | 2 | 314,338 | 8.08 ± 6.79 | 20.95 ± 8.87 | 25.14 ± 7.86 | 45.16 ± 9.34 |
| La Porte (IN) | 2 | 108,383 | 8.37 ± 3.99 | 15.48 ± 4.22 | 17.21 ± 3.62 | 27.12 ± 4.59 |
| Las Vegas (NV) | 8 | 2,074,364 | 69.48 ± 39.92 | 124.15 ± 47.88 | 147.78 ± 41.15 | 214.97 ± 47.77 |
| Lake Charles (LA) | 6 | 189,618 | 7.49 ± 8.47 | 18.65 ± 3.67 | 22.11 ± 5.97 | 38.62 ± 6.21 |
| Logan (UT) | 2 | 111,182 | 2.65 ± 3.19 | 6.09 ± 1.9 | 7.84 ± 1.77 | 12.43 ± 2.76 |
| Los Angeles (CA) | 5 | 10,559,243 | 915.04 ± 530.45 | 1368.12 ± 336.21 | 1608.39 ± 308.91 | 2078.49 ± 407.23 |
| Louisville (KY) | 3 | 738,978 | 41.81 ± 18.06 | 79.13 ± 15.44 | 83.19 ± 12.41 | 126.03 ± 17.75 |
| Little Rock (AR) | 4 | 380,611 | 18.07 ± 10 | 36.45 ± 12.02 | 43.43 ± 13.97 | 69.79 ± 18.82 |
| Macon (GA) | 4 | 157,307 | 5.93 ± 3.14 | 11.22 ± 2.75 | 13.45 ± 2.97 | 21.26 ± 2.43 |
| Madison (IL) | 3 | 273,407 | 26.79 ± 12.8 | 46.42 ± 8.78 | 47.95 ± 6.02 | 68.81 ± 10.69 |
| Madison (WI) | 2 | 571,173 | 15.73 ± 12.12 | 42.02 ± 14.77 | 50.2 ± 13.83 | 88.61 ± 16.45 |
| Upper Marlboro (MD) | 1 | 895,794 | 69.88 ± 21.38 | 105.73 ± 17.24 | 115.14 ± 15.68 | 173.39 ± 21.06 |
| Mcallen (TX) | 7 | 762,535 | 14.68 ± 6.34 | 27.01 ± 5.22 | 33.96 ± 6.51 | 45.02 ± 6.23 |
| Medford (OR) | 9 | 197,576 | 4.37 ± 4.79 | 10.39 ± 4.38 | 12.94 ± 5.43 | 21.1 ± 6.55 |
| Melbourne (FL) | 7 | 547,450 | -3.66 ± 9.27 | 7.79 ± 6.57 | 14.45 ± 6.38 | 35.68 ± 4.86 |
| Memphis (TN) | 4 | 954,592 | 55.77 ± 24.97 | 101.01 ± 18.86 | 112.36 ± 16.89 | 168.6 ± 30.26 |
| Mercer (PA) | 2 | 112,377 | 5.64 ± 5.73 | 10.73 ± 3.94 | 12.73 ± 3.71 | 23.22 ± 4.1 |
| Boise City (ID) | 9 | 386,522 | 7.24 ± 5.48 | 15.86 ± 5.43 | 18.98 ± 5.47 | 28.16 ± 7.52 |
| Miami (FL) | 7 | 3,099,526 | 56.56 ± 32.1 | 110.65 ± 44.05 | 146.65 ± 36.82 | 230.92 ± 24.77 |
| Middlesex (NJ) | 1 | 881,321 | 66.7 ± 26.04 | 103.1 ± 19.77 | 112.95 ± 18.84 | 171.92 ± 22.03 |
| Middletown (OH) | 3 | 383,546 | 5.2 ± 6.77 | 20.34 ± 7.21 | 23.29 ± 6.43 | 41.88 ± 8.67 |
| Milwaukee (WI) | 2 | 1,357,450 | 53.8 ± 56.35 | 113.75 ± 45.58 | 133.94 ± 41.2 | 238.98 ± 50.23 |
| Minneapolis (MN) | 2 | 1,743,595 | 71.22 ± 38.95 | 150.37 ± 42.16 | 176.26 ± 39.81 | 278.33 ± 50.64 |
| Mobile (AL) | 6 | 417,866 | 7.83 ± 10.48 | 21.84 ± 6.15 | 24.75 ± 7.89 | 53.56 ± 9.21 |
| Modesto (CA) | 8 | 573,131 | -20.62 ± 15.96 | -7.34 ± 9.59 | -1.19 ± 10.27 | 13.2 ± 14.39 |
| Toms River (NJ) | 1 | 587,588 | 70.58 ± 23.9 | 108.91 ± 19.92 | 118.86 ± 18.97 | 177.78 ± 21.54 |
| Monroe (LA) | 4 | 153,883 | 10.14 ± 3.73 | 20.46 ± 4.63 | 23.85 ± 6.44 | 36.64 ± 6.55 |
| Montgomery (AL) | 6 | 228,548 | 7.16 ± 6.96 | 14.96 ± 3.77 | 17.77 ± 4.17 | 31.99 ± 4.43 |
| Muncie (IN) | 3 | 114,470 | -2.48 ± 3.83 | 3.1 ± 2.97 | 4.3 ± 2.77 | 11.53 ± 3.77 |
| Muskegon (MI) | 2 | 177,808 | 3.07 ± 4.92 | 12.06 ± 5.7 | 15.24 ± 5.41 | 30.81 ± 6.6 |
| Myrtle Beach (SC) | 4 | 254,638 | 10.59 ± 5.43 | 16.85 ± 4.39 | 20.23 ± 4.83 | 36.23 ± 4.86 |
| Nampa (ID) | 9 | 185,267 | 3.63 ± 2.88 | 7.94 ± 2.9 | 9.73 ± 2.95 | 15.3 ± 4.54 |
| Nashua (NH) | 2 | 437,603 | 35.74 ± 13.51 | 53.95 ± 10.26 | 60.53 ± 11.44 | 90.51 ± 8.42 |
| Nashville (TN) | 4 | 652,834 | 3.76 ± 10.79 | 23.83 ± 9.63 | 28.83 ± 8.42 | 57.15 ± 11.11 |
| Melville (NY) | 1 | 3,000,255 | 97.04 ± 100.82 | 207.35 ± 72.03 | 239.06 ± 69.84 | 440.94 ± 83.71 |
| Newark (NJ) | 1 | 1,365,331 | 121.21 ± 31.34 | 185.97 ± 34.22 | 202.89 ± 32.46 | 304.26 ± 37.21 |
| Newburgh (NY) | 1 | 419,658 | 8.91 ± 8.23 | 21.88 ± 8.52 | 26.28 ± 8.73 | 52.59 ± 9.51 |
| New Haven (CT) | 1 | 872,769 | 40.48 ± 23.03 | 81.13 ± 22.85 | 93.9 ± 23.97 | 166.31 ± 21.95 |
| New London (CT) | 1 | 269,894 | 1.94 ± 8.37 | 11.6 ± 5.53 | 14.77 ± 5.87 | 32.98 ± 5.75 |
| Niles (MI) | 2 | 162,189 | 12.18 ± 6.3 | 22.97 ± 6.65 | 25.7 ± 5.69 | 41.37 ± 7.38 |
| Norfolk (VA) | 4 | 1,514,213 | 6.58 ± 18.14 | 35.96 ± 17.58 | 46.02 ± 16.35 | 111.27 ± 26.25 |
| New Orleans (LA) | 6 | 945,703 | 38.78 ± 32.91 | 93.58 ± 18.51 | 102.61 ± 26.88 | 175.53 ± 25.12 |
| New York (NY) | 1 | 10,396,046 | -198.35 ± 206.98 | 51.28 ± 180.59 | 131.02 ± 176.25 | 648.39 ± 223.72 |
| Oakland (CA) | 5 | 2,651,188 | -77.33 ± 39.17 | -25.04 ± 30.38 | -8.19 ± 38.33 | 77.96 ± 60.64 |
| Ocala (FL) | 6 | 327,455 | -2.76 ± 6.16 | 4.79 ± 5.3 | 7.76 ± 5.97 | 38.9 ± 6.24 |
| Oklahoma City (OK) | 4 | 732,376 | 30.34 ± 21.5 | 64.11 ± 21.34 | 81.03 ± 25.08 | 120.24 ± 22.67 |
| Omaha (NE) | 2 | 525,046 | 52.22 ± 14.03 | 84.81 ± 12.97 | 88.37 ± 9.61 | 128.55 ± 15.79 |
| Orlando (FL) | 7 | 1,617,589 | -19.85 ± 15.39 | 8.98 ± 13.82 | 17.8 ± 12.82 | 80.12 ± 9.94 |
| Ottawa (IL) | 2 | 110,661 | 8.33 ± 3.85 | 14.88 ± 3.18 | 15.95 ± 2.77 | 24.81 ± 3.52 |
| Port Arthur (TX) | 6 | 262,282 | 4.24 ± 4.44 | 14.58 ± 4.1 | 20.23 ± 6.23 | 34.7 ± 6.62 |
| Palm Beach (FL) | 7 | 1,363,061 | -1.22 ± 25.16 | 29.67 ± 19.6 | 48.64 ± 17.63 | 96.53 ± 12.71 |
| Pensacola (FL) | 6 | 311,251 | 4.05 ± 5.05 | 12.26 ± 4.3 | 14.44 ± 5.3 | 35.32 ± 7.33 |
| Philadelphia (PA) | 1 | 4,875,946 | 445.6 ± 154.37 | 690.13 ± 130.1 | 758.76 ± 121.06 | 1178.53 ± 155.62 |
| Phoenix (AZ) | 8 | 3,828,993 | 430.3 ± 64.69 | 563.31 ± 78.23 | 626.71 ± 58.71 | 751.47 ± 81.53 |
| Pittsburgh (PA) | 3 | 1,237,627 | -20.31 ± 32.85 | 30.29 ± 29.91 | 46.96 ± 28.55 | 132.18 ± 34.29 |
| Plymouth (MA) | 1 | 508,851 | 3.65 ± 14.14 | 14.12 ± 11.62 | 19.99 ± 12.3 | 52.26 ± 12.91 |
| Portage (IN) | 2 | 169,889 | 17.47 ± 5.24 | 27.42 ± 5.43 | 29.42 ± 4.61 | 42.5 ± 5.67 |
| Portland (OR) | 5 | 1,627,866 | 19.84 ± 31.45 | 79.14 ± 52.59 | 100.65 ± 49.77 | 173.27 ± 42.25 |
| Portland (ME) | 2 | 283,360 | -4.78 ± 4.3 | 3.79 ± 5.49 | 8.5 ± 7.16 | 26.8 ± 6.05 |
| Providence (RI) | 1 | 837,540 | 15.64 ± 17.21 | 39.52 ± 21.9 | 50.57 ± 23.1 | 111.55 ± 23.61 |
| Provo (UT) | 9 | 509,040 | 5.33 ± 4.94 | 15.09 ± 5.04 | 19.67 ± 4.54 | 32.72 ± 9.34 |
| Raleigh (NC) | 4 | 874,071 | -0.5 ± 7.75 | 14.62 ± 6.98 | 18.96 ± 7.15 | 49.91 ± 8.99 |
| Reading (PA) | 1 | 424,666 | 36.47 ± 14.68 | 60.38 ± 12.91 | 67.09 ± 11.81 | 106 ± 13.71 |
| Reno (NV) | 9 | 470,501 | 6.91 ± 11.14 | 18.42 ± 8.2 | 24.99 ± 8.74 | 42.16 ± 14.18 |
| Richmond (VA) | 1 | 863,737 | 112.23 ± 23.4 | 160.69 ± 21.03 | 170.25 ± 18.36 | 240.64 ± 23.27 |
| Riverside (CA) | 5 | 4,249,734 | 637.28 ± 130.5 | 803.14 ± 125.23 | 896.84 ± 103.75 | 1083.3 ± 133.39 |
| Rochester (NY) | 2 | 785,278 | 21.4 ± 18.45 | 52.63 ± 23.17 | 65.24 ± 24.36 | 123.79 ± 27.27 |
| Rockville (MD) | 1 | 993,900 | 103.06 ± 33.65 | 143.93 ± 17.17 | 152.01 ± 15.46 | 206.76 ± 18.34 |
| Sacramento (CA) | 5 | 1,443,068 | 195.6 ± 44.42 | 270.72 ± 57.07 | 305.92 ± 54.48 | 383.56 ± 75.72 |
| Essex (MA) | 1 | 756,210 | 17.09 ± 24.75 | 38.09 ± 16.33 | 49.36 ± 18.86 | 99.8 ± 15.86 |
| Salt Lake City (UT) | 9 | 1,063,702 | 33.18 ± 15.33 | 59.65 ± 13.16 | 72.62 ± 10.23 | 102.07 ± 21.62 |
| San Antonio (TX) | 6 | 1,685,188 | 70.04 ± 47.13 | 115.17 ± 25.93 | 153.83 ± 39.22 | 218.33 ± 36.85 |
| Sarasota (FL) | 7 | 705,040 | 5.05 ± 16.47 | 24.71 ± 7.72 | 33.29 ± 8.19 | 64.17 ± 6.33 |
| Santa Barbara (CA) | 5 | 425,999 | -0.29 ± 10.96 | 12.53 ± 8.19 | 20.46 ± 9.36 | 38.5 ± 13.74 |
| South Bend (IN) | 2 | 282,297 | 17.81 ± 9.07 | 33.36 ± 9.55 | 37.38 ± 8.17 | 60.21 ± 10.59 |
| St. Charles (MO) | 3 | 377,645 | 22.94 ± 7.55 | 41.4 ± 8.71 | 42.88 ± 5.87 | 63.75 ± 10.67 |
| East St. Louis (IL) | 3 | 266,433 | 22.81 ± 12.27 | 40.41 ± 7.98 | 41.82 ± 5.45 | 61.04 ± 9.81 |
| State College (PA) | 2 | 165,012 | 5.29 ± 6.3 | 12.64 ± 4.11 | 14.61 ± 3.68 | 26.52 ± 4.32 |
| Scranton (PA) | 2 | 503,640 | 24.81 ± 17.28 | 53.53 ± 16.96 | 62.15 ± 16.58 | 111.89 ± 18.17 |
| San Diego (CA) | 5 | 3,076,373 | 253.53 ± 136.63 | 357.35 ± 67.27 | 411.9 ± 52.79 | 525.11 ± 71.1 |
| Seattle (WA) | 5 | 2,095,098 | -104.25 ± 57.3 | -66.17 ± 36.54 | -51.45 ± 32.38 | 5.43 ± 30.35 |
| San Francisco (CA) | 5 | 1,474,044 | -26.22 ± 24.4 | -0.97 ± 15.73 | 6.74 ± 20.4 | 55.1 ± 34.89 |
| Sioux City (IA) | 2 | 104,608 | 10.47 ± 3.54 | 18.68 ± 3.32 | 20.15 ± 2.74 | 31.48 ± 4.5 |
| San Jose (CA) | 5 | 1,773,974 | 163.32 ± 64.33 | 237.41 ± 50.89 | 263.67 ± 49.32 | 329.23 ± 63.03 |
| Spartanburg (SC) | 4 | 290,717 | 1.47 ± 8.4 | 9.88 ± 5.35 | 13.63 ± 5.63 | 29.31 ± 5.53 |
| St. Petersburg (FL) | 7 | 947,997 | 40.12 ± 26.71 | 69.12 ± 11.91 | 82.82 ± 12.97 | 134.9 ± 9.56 |
| Spokane (WA) | 9 | 455,457 | -8.36 ± 8.65 | 5.14 ± 12.74 | 7.92 ± 9.9 | 20.88 ± 8.33 |
| Springfield (MA) | 1 | 470,398 | 20.8 ± 16.8 | 42.34 ± 12.61 | 50.38 ± 14.36 | 88.45 ± 12.06 |
| Springfield (MO) | 3 | 262,664 | 11.35 ± 6.33 | 26.62 ± 8.1 | 28.54 ± 6 | 45.97 ± 13.01 |
| Stamford (CT) | 1 | 929,629 | 15.78 ± 16.68 | 46.64 ± 17.61 | 57.04 ± 18.75 | 118.06 ± 18.2 |
| Steubenville (OH) | 3 | 108,793 | -0.08 ± 4.07 | 4.62 ± 2.68 | 6.16 ± 2.64 | 13.96 ± 3.4 |
| St. Louis (MO) | 3 | 1,620,561 | 151.73 ± 70.34 | 258.34 ± 47.4 | 268.04 ± 33.07 | 383.84 ± 59.49 |
| Stockton (CA) | 5 | 726,034 | 121.47 ± 19.32 | 152.86 ± 24.81 | 166.17 ± 23.56 | 197.96 ± 30.39 |
| Tacoma (WA) | 5 | 801,618 | -19.48 ± 28.32 | -0.33 ± 19.16 | 7.21 ± 16.78 | 36.84 ± 16.72 |
| Tallahassee (FL) | 6 | 286,424 | -0.73 ± 2.83 | 4.48 ± 3.36 | 5.68 ± 3.31 | 18.48 ± 4.13 |
| Tampa (FL) | 7 | 1,322,129 | -2.09 ± 12.97 | 21.39 ± 10.5 | 34.11 ± 11.3 | 81.4 ± 8.71 |
| Terre Haute (IN) | 3 | 101,706 | 1.51 ± 1.81 | 7.79 ± 2.74 | 8.46 ± 2.38 | 16.05 ± 3.33 |
| Toledo (OH) | 2 | 464,712 | 37.54 ± 21.62 | 68.72 ± 16.73 | 76.49 ± 14.73 | 112.44 ± 16.17 |
| Topeka (KS) | 3 | 177,127 | 8.57 ± 4.09 | 19.37 ± 4.84 | 20.14 ± 3.8 | 31.03 ± 7.14 |
| Trenton (NJ) | 1 | 392,619 | 28.19 ± 10.68 | 44.48 ± 9.38 | 49.15 ± 8.97 | 77.71 ± 10.66 |
| Tucson (AZ) | 8 | 980,969 | 7.18 ± 17.16 | 33.64 ± 18.2 | 54.15 ± 12.72 | 80.18 ± 12.63 |
| Tulsa (OK) | 4 | 603,879 | 37.01 ± 19.3 | 71.01 ± 20.09 | 83.1 ± 21.07 | 123.23 ± 28.29 |
| Vancouver (WA) | 5 | 489,556 | 3.84 ± 8.73 | 21.65 ± 15.5 | 28.03 ± 14.82 | 49.61 ± 12.37 |
| Ventura (CA) | 5 | 905,979 | 10.4 ± 31.61 | 36.31 ± 16.26 | 51.52 ± 18.44 | 85.4 ± 25.63 |
| Visalia (CA) | 8 | 478,651 | -16.98 ± 9.68 | -5.87 ± 8.01 | 0.38 ± 8.17 | 14.47 ± 12.76 |
| Washington (PA) | 3 | 196,082 | -5 ± 7.57 | 2.25 ± 4.33 | 4.82 ± 4.34 | 17.67 ± 5.58 |
| Washington (DC) | 1 | 810,015 | 112.2 ± 22.81 | 159.05 ± 20.06 | 168.63 ± 18.01 | 233.29 ± 21.5 |
| Ogden (UT) | 9 | 224,311 | 12.77 ± 5.18 | 20.33 ± 3.69 | 23.71 ± 3.33 | 31.98 ± 5.65 |
| Wichita (KS) | 3 | 506,519 | 41.5 ± 13.73 | 68.7 ± 15.46 | 77.45 ± 16.9 | 107.63 ± 21.17 |
| Wilmington (DE) | 1 | 556,014 | 43.83 ± 11.13 | 70.25 ± 13.17 | 77.17 ± 12.24 | 119.71 ± 15.74 |
| Winston-Salem (NC) | 4 | 340,276 | 1.26 ± 6.87 | 12.31 ± 5.21 | 15.33 ± 5.08 | 34.41 ± 6.35 |
| Worcester (MA) | 2 | 804,868 | 0.03 ± 14.27 | 23.97 ± 16.07 | 34.84 ± 18.83 | 85.29 ± 15.7 |
| York (PA) | 1 | 419,797 | 27.11 ± 11.08 | 47.14 ± 10.75 | 52.75 ± 9.8 | 85.56 ± 11.61 |
| Youngstown (OH) | 2 | 453,322 | 15.18 ± 12.99 | 38.57 ± 14.56 | 46.25 ± 14.27 | 85.5 ± 16.38 |
